# Identifying Genes Involved in Alkaloid Biosynthesis in *Vinca minor* through Transcriptomics and Gene Co-Expression Analysis

**DOI:** 10.3390/biom10121595

**Published:** 2020-11-24

**Authors:** Emily Amor Stander, Liuda Johana Sepúlveda, Thomas Dugé de Bernonville, Inês Carqueijeiro, Konstantinos Koudounas, Pamela Lemos Cruz, Sébastien Besseau, Arnaud Lanoue, Nicolas Papon, Nathalie Giglioli-Guivarc’h, Ron Dirks, Sarah Ellen O’Connor, Lucia Atehortùa, Audrey Oudin, Vincent Courdavault

**Affiliations:** 1 EA2106 “Biomolécules et Biotechnologies Végétales”, Université de Tours, 37200 Tours, France; emily.stander@univ-tours.fr (E.A.S.); liudayohana@gmail.com (L.J.S.); thomas.duge@univ-tours.fr (T.D.d.B.); ines.carqueijeiro@univ-tours.fr (I.C.); koudounas@univ-tours.fr (K.K.); pamela.lemos@univ-tours.fr (P.L.C.); sebastien.besseau@univ-tours.fr (S.B.); arnaud.lanoue@univ-tours.fr (A.L.); nathalie.guivarch@univ-tours.fr (N.G.-G.); 2 Laboratorio de Biotecnología, Sede de Investigación Universitaria, Universidad de Antioquia, Antioquia Medellin 050021, Colombia; latehor@gmail.com; 3 Host-Pathogen Interaction Study Group (GEIHP, EA 3142), UNIV Angers, UNIV Brest, 49933 Angers, France; nicolas.papon@univ-angers.fr; 4 Future Genomics Technologies, 2333 BE Leiden, The Netherlands; dirks@futuregenomics.tech; 5 Department of Natural Product Biosynthesis, Max Planck Institute for Chemical Ecology, 07745 Jena, Germany; oconnor@ice.mpg.de

**Keywords:** alkaloids, *Vinca minor*, RNA-seq, transcriptomics, differential gene expression, *O*-methyltransferase

## Abstract

The lesser periwinkle *Vinca minor* accumulates numerous monoterpene indole alkaloids (MIAs) including the vasodilator vincamine. While the biosynthetic pathway of MIAs has been largely elucidated in other Apocynaceae such as *Catharanthus roseus*, the counterpart in *V. minor* remains mostly unknown, especially for reactions leading to MIAs specific to this plant. As a consequence, we generated a comprehensive *V. minor* transcriptome elaborated from eight distinct samples including roots, old and young leaves exposed to low or high light exposure conditions. This optimized resource exhibits an improved completeness compared to already published ones. Through homology-based searches using *C. roseus* genes as bait, we predicted candidate genes for all common steps of the MIA pathway as illustrated by the cloning of a tabersonine/vincadifformine 16-*O*-methyltransferase (Vm16OMT) isoform. The functional validation of this enzyme revealed its capacity of methylating 16-hydroxylated derivatives of tabersonine, vincadifformine and lochnericine with a Km 0.94 ± 0.06 µM for 16-hydroxytabersonine. Furthermore, by combining expression of fusions with yellow fluorescent proteins and interaction assays, we established that Vm16OMT is located in the cytosol and forms homodimers. Finally, a gene co-expression network was performed to identify candidate genes of the missing *V. minor* biosynthetic steps to guide MIA pathway elucidation.

## 1. Introduction

Monoterpene indole alkaloids (MIAs) are a class of specialized plant metabolites found in Gentianale order families; Apocynaceae, Rubiaceae and Loganiaceae. MIAs have important roles in plant defense [1,2,3] and constitute a large chemical diversity of pharmacophores with a variety of potent biological activities. These activities make MIAs attractive pharmaceutical drugs with high commercial value, such as the anticancer drugs vinblastine and vincristine from *Catharanthus roseus*, the antiarrhythmic agent ajmaline from *Rauvolfia serpentina* and the vasodilator vincamine, from *Vinca minor*, used to treat cerebrovascular disorders. MIA producing plants vary a lot in biomass production and alkaloid content, prompting research efforts towards biosynthetic pathway elucidation. This knowledge has the potential to stimulate alternative engineering approaches, including synthetic biology and metabolic engineering, for the upscaled commercial production of these valuable natural products [4,5,6].

*V. minor*, also known as the lesser periwinkle, is an evergreen herbaceous shrub belonging to the Apocynaceae family. In addition to the above mentioned pharmaceutically important MIA vincamine, this plant produces more than 50 indole alkaloids, including t less known interesting molecules such as lochnericine, akuammicine, vincadifformine, minovincinine [7,8] and picrinine [9]. To date, publicly available *V. minor* genomic resources are restricted to the 454 based- (V_minor_v1) and illumina based transcriptomes (V_minor_v3) from the PhytoMetaSyn database (https://bioinformatics.tugraz.at/phytometasyn/). Furthermore, knowledge on MIA biosynthetic pathways in *V. minor* is lacking and mainly results from homology-based identification of genes with its relative *C. roseus* sharing high genetic and metabolic similarities [8,10]. Recently, Verma et al. (2020) [10] identified 12 putative MIA biosynthetic genes in *V. minor* originating from either the *Rauwolfia* raucaffricine-type alkaloid pathway, or the vindoline pathway in *C. roseus*. Furthermore, a *V. minor* counterpart of the *C. roseus* TPT2 transporter, involved in the secretion of MIAs to the leaf surface, has also been characterized [8] together with a picrinine *N*-methyltransferase involved in ervincine biosynthesis [11]. At the metabolic level, while the intracellular distribution of MIAs is still a matter of debate in leaves of *V. minor* [8,12], experimental evidence tends to confirm that MIA metabolism is higher in younger tissues and notably young leaves [13,14,15]. In addition, vincamine has been described as the main alkaloid found in the leaves of *V. minor* (0.13%) whereas 1,2-dehydroaspidospermidine is the major alkaloid detected in the roots (0.28% [16]). Finally, some alkaloids are also specific to *V. minor* leaves, including vincarubine, picrinine, eburnamine, eburnamonine, minovincinine, minovine, vincadifformine and strictamine [7,8,12,16]. 

**Figure 1:**
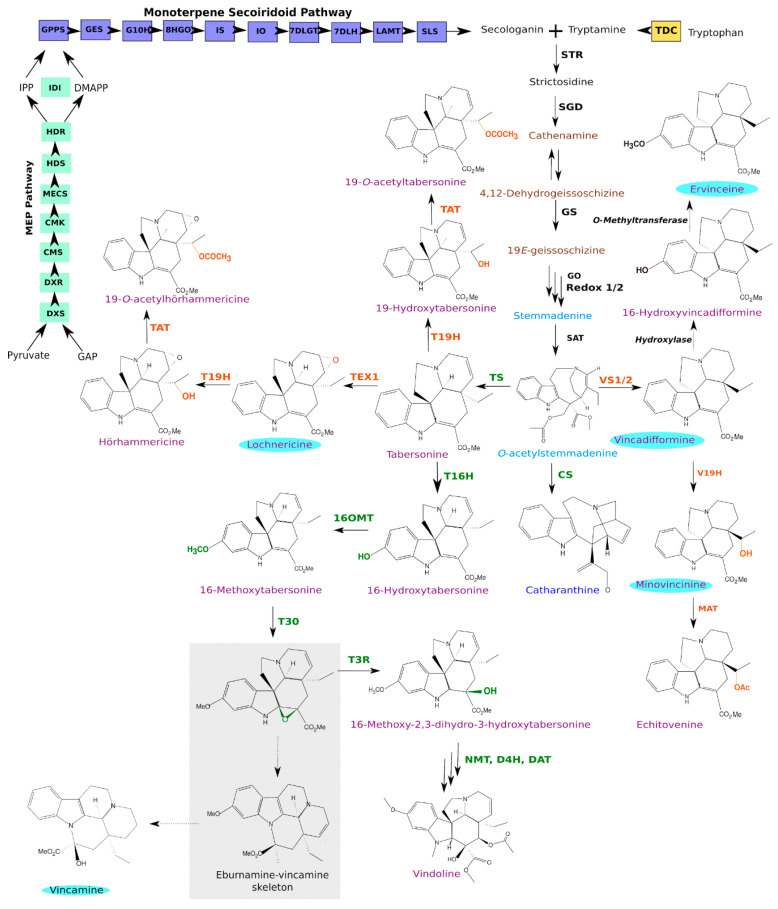
The elucidated MIA biosynthetic pathway in *Catharanthus roseus.* Enzymes catalyzing pathways in the aerial parts of the plant are depicted in green, and enzymes in the roots are depicted in orange. MIA names highlighted in light blue circles have also been identified in *Vinca minor*. Depicted in the grey box is the rearrangement of the T30 epoxide product, forming an eburnamine-vincamine like skeleton, in cases where T3R is silenced as described in [28]. The different MIA scaffold classes are depicted by MIA names written in dark blue (iboga-type), light blue (strychnos-type), brown (corynanthe) and purple (aspidosperma). Uncharacterized reactions are depicted with dashed arrows. MEP, methyl-D-erythritol phosphate; GAP, glyceraldehyde 3-phosphate; DXS, 1-deoxy-D-xylulose 5-phosphate synthase; DXR, 1-deoxy-D-xylulose 5-phosphate reductoisomerase; CMS, 2-C-methyl-D-erythritol 4-phosphate synthase; CMK, 4-(cytidine 5′diphospho)-2-C-methyl-D-erythritol kinase; MECS, 2-C-methyl-D-erythritol 2,4-cyclodiphosphate synthase; HDS, (E)-4-hydroxy-3-methylbut-2-enyl diphosphate synthase; HDR, (E)-4-hydroxy-3-methylbut-2-enyl diphosphate reductase; IPP, isopentenyl diphosphate; DMAPP, dimethylallyl diphosphate; IDI, isopentenyl diphosphate isomerase; GPPS, geranyl diphosphate synthase; GES, geraniol synthase; G10H, geraniol 10-hydroxylase; 10HGO, 10-hydroxygeraniol oxidoreductase; IO, iridoid oxidase; IS, iridoid synthase; 7DLGT, 7-deoxyloganetic acid glucosyltransferase; 7DLH, 7-deoxyloganic acid 7-hydroxylase; LAMT, loganic acid O-methyltransferase; SLS, secologanin synthase; TDC, tryptophan decarboxylase; STR, strictosidine synthase; SGD, strictosidine β-glucosidase; GS, geissoschizine synthase; GO, geissoschizine oxidase; SAT, Stemmadenine O-acetyltransferase; CS, catharanthine synthase; VS1/2, vincadifformine synthase 1/2; V19H, vincadifformine 19-hydroxylase; MAT, minovincine 19-O-acetyltransferase; TS, tabersonine synthase; TEX, tabersonine 6,7-epoxidase; T19H, tabersonine 19-hydroxylase; TAT, tabersonine derivative 19-O-acetyltransferase; T16H, tabersonine 16-hydroxylase; 16OMT, 16-hydroxytabersonine O-methyltransferase; T3O, tabersonine 3-oxidase; T3R, tabersonine 3-reductase; NMT, 16-methoxy-2,3-dihydrotabersonine N-methyltransferase; D4H, desacetoxyvindoline 4-hydroxylase; DAT, deacetylvindoline 4-O-acetyltransferase.

Far from the poorly described biosynthetic routes of *V. minor*, a combination of pioneering works on *C. roseus* has culminated in the near-complete elucidation of MIA biosynthesis in the leaves of this “model” non-model plant [17,18,19,20,21]. By contrast, the MIA biosynthetic pathway in the roots of *C. roseus* still remains elusive to a certain extent [22,23,24,25]. Regardless of the MIA accumulating organs, almost all MIAs originate from a central unique skeleton, strictosidine, which is the product of a condensation reaction between tryptamine (the indole ring donor) and secologanin (the terpenoid donor) by strictosidine synthase (STR; Figure 1) [26,27].

While tryptamine results from a single-step decarboxylation of tryptophan by tryptophan decarboxylase (TDC) [29,30], the synthesis of secologanin successively involves the methyl-erythritol phosphate (MEP) pathway and the monoterpene secoiridoid pathway producing secologanin. The MEP pathway successively involves 1-deoxy-D-xylulose 5-phosphate (DXP) synthase (DXS), DXP reductoisomerase (DXR), 4-(cytidine 5’diphospho)-2-C-methyl-D-erythritol synthase (CDP-ME) synthase (CMS), CDP-ME kinase (CMK), 2-C-methyl-D-erythritol 2,4-cyclodiphosphate synthase (MECS), (E)-4-hydroxy-3-methylbut-2-enyl diphosphate (HMBPP) synthase (HDS) and HMBPP reductase (HDR). In association with IDI isomerase, it produces a controlled ratio of isopentenyl diphosphate (IPP) and dimethylallyl diphosphate (DMAPP) that are further condensed by geranyl diphosphate (GPP) synthase (GPPS) to form GPP [31,32,33,34,35]. The monoterpene secoiridoid pathway then converts GPP through the concerted action of geraniol synthase (GES) [36], geraniol 10-hydroxylase (G10H) [37], 10-hydroxygeraniol oxidoreductase (10HGO) [38], iridoid synthase (IS) [39], iridoid oxidase (IO) [40], 7-deoxyloganetic acid glucosyltransferase (7DLGT) [41], 7-deoxyloganic acid (7DLH) [42], loganic acid-O-methyltransferase (LAMT) [14] and secologanin synthase (SLS) [19,43]. The resulting strictosidine is then deglycosylated by strictosidine β-D-glucosidase (SGD) to form reactive intermediates, including 4,21-dehydrogeissoschizine and cathenamine, which initiate distinct MIA biosynthetic branches [44,45,46,47]. The gateway step in iboga- and aspidosperma-type MIA biosynthesis involves geissoschizine synthase (GS) that converts 4,21-dehydrogeissoschizine to 19*E*-geissoschizine [48]. This is followed by five additional enzymes that catalyze the biosynthesis of the common intermediate, *O*-acetylstemmadenine, which lies at the branching point between three different pathways [21,49]. In both above and underground parts of the plant, catharanthine synthase (CS) catalyzes the formation of the iboga MIA, catharanthine that is one of the structural monomers that joins with the aspidosperma MIA, vindoline to form precursors of the important anticancer drugs vinblastine and vincristine. Additionally, tabersonine synthase (TS) catalyzes the conversion of *O*-acetylstemmadenine towards the vindoline pathway via the important intermediate tabersonine [49]. Finally, in the roots of *C. roseus*, vincadifformine synthase 1 or 2 (VS1/2) catalyzes the formation of vincadifformine, which is converted into minovincinine by vincadifformine 19-hydroxylase (V19H) that in turn undergoes acetylation by minovincinine 19-hydroxy-O-acetyltransferase (MAT) to form echitovenine [22,25].

The metabolism of tabersonine, and to a lesser extent vincadifformine, is of primary importance in many Apocynaceae, notably in *C. roseus* and *V. minor*. For instance, tabersonine is converted into vindoline in leaves of *C. roseus* through a seven-step biosynthetic route [50]. These decorations start with an hydroxylation at the C16 position catalyzed by tabersonine-16-hydroxylase (T16H) [51,52,53], followed by a methylation at the same position by tabersonine 16-*O*-methyltransferase (16OMT), forming 16-methoxytabersonine [15,54]. A subsequent epoxidation of 16-methoxytabersonine at the C2/C3 position by 16-methoxytabersonine 3-oxygenase (T3O), results in an unstable epoxide [28], which in turn is converted to 16-methoxy-2,3-dihydro-3-hydroxy-tabersonine by tabersonine-3-reductase (T3R) [50]. This compound is then subjected to *N*-methylation, hydroxylation and acetylation respectively catalyzed by 16-methoxy-2,3-dihydro-3-hydroxytabersonine *N*-methyltransferase (NMT) [55], desacetoxyvindoline-4-hydroxylase (D4H) [56] and deacetylvindoline-4-*O*-acetyltransferase (DAT) [57] to form vindoline. Light was reported to be an important stimulator of the vindoline biosynthetic pathway [54,58] by activating the last two steps of the pathway involving D4H and DAT [56,58,59].

By contrast, in *C. roseus* roots, tabersonine is epoxidized at the C6, C7 position by tabersonine 6,7-epoxidase 1 (TEX1) to yield lochnericine [23], which is subsequently hydroxylated by tabersonine 19-hydroxylase (T19H) [60] and acetylated by tabersonine-derivatives acetyltransferase (TAT) [24] to form 19-*O*-acetylhörhammericine. Interestingly, a mirroring biosynthetic route allows converting vincadifformine into echitovenine through the concerted action of vincadifformine 19-hydroxylase (V19H) [25] and minovincinine acetyltransferase (MAT) [22]. While the presence of tabersonine and derivatives in *V. minor* has been reported in a few works only [8,61], vincadifformine and vincamine are accumulated at high levels [12]. Importantly, epoxide formation on a vincadifformine-like skeleton has been proposed to be part of the vincamine synthesis process leading to postulate that vincadifformine is a precursor of vincamine [62]. While orthologues of *C. roseus* VS1/2 can be easily identified, no enzyme involved in the vincadifformine/vincamine conversion has been identified to date. However, during T3O characterization, Kellner et al. (2015) [28] established that in the absence of T3R, the rearrangement of the epoxide product resulted in the accumulation of an eburnamine-vincamine skeleton product, which *C. roseus* does not naturally accumulate [63]. This thus suggested that a T3O like enzyme from *V. minor* could be involved in vincamine biosynthesis. Above all, it further highlights the necessity to pursue identification of MIA biosynthetic genes in *V. minor*.

While the elucidation of alkaloid biosynthetic routes has always been a laborious task, emerging omics-based strategies now facilitate the identification of genes from these complex pathways, notably through transcriptomics and gene co-expression analyses combined to functional genomic approaches [6,64]. Over the last five years, the efficiency of such a strategy has been clearly established by elucidating many missing steps of the MIA biosynthetic pathway in *C. roseus.* In this respect, the value of a high-quality reference transcriptome in discovering novel isoforms was also demonstrated, by identifying a second secologanin synthase (SLS2) catalyzing the last step of the synthesis of the monoterpene precursor of MIAs [19]. Therefore, the identification of missing MIA enzymes and isoforms appears to be crucial to elucidate the complexity of MIA metabolism as illustrated for cytochrome P450 reductases whose isoforms are associated to primary or specialized metabolism [65], the organ-specific distribution of T16H1 and T16H2 [53] and the enantiospecificity of T19H and V19H [25]. Furthermore, identification of novel isoforms across species is an ongoing endeavor that may allow for the discovery of more efficient enzymes to be applied in synthetic biology approaches for increased biosynthetic rates in newly developed cell factories [66,67].

In our continuing effort to elucidate and engineer MIA biosynthesis, we present here a comprehensive consensus transcriptome resource for *V. minor* comprising eight different tissue types and experimental conditions. This new *V. minor* reference transcriptome displays improved BUSCO scores to the previously available 454 based (V_minor_v1) and illumina based assemblies (V_minor_v3) from the PhytoMetaSyn database (https://bioinformatics.tugraz.at/phytometasyn/). This improved resource was thus used to rationally predict candidate genes for all known steps of the *V. minor* MIA pathway as illustrated by the functional validation of a tabersonine/vincadifformine 16-*O*-methyltransferase (16OMT) isoform. Finally, a gene co-expression analysis was performed to predict new MIA biosynthetic candidate genes to deepen our knowledge on the MIA metabolism in *V. minor*.

## 2. Materials and Methods

### 2.1. Chemicals

Vincadifformine was prepared by the hydrogenation of tabersonine as described in [68]. Lochnericine, 19-hydroxytabersonine and 16-hydroxytabersonine were produced by the 6,7-epoxidation of tabersonine, the 19-hydroxylation or 16-hydroxylation of tabersonine by yeast expressing TEX1, T19H or T16H2 from *C. roseus*, respectively [23,53]. S-adenosyl-L-methionine (SAM) was purchased from Sigma-Aldrich (St-Quentin-Fallavier, France).

### 2.2. Plant Growth Conditions and Sample Collection

Wild growing *V. minor* plants were collected from the center of France (47°21′11.4″ N 0°42′08.2″ E) and transferred to greenhouse (24 °C; 16 h light/8 h dark cycle). Stems were all removed to allow new shoots to develop during two months. In these conditions, two batches of ten plants were exposed to high (3300 lux) or low (250 lux) illumination conditions to generate the light and shadow conditions. Young leaves (YL) and old leaves (OL) stand for the first and fourth pair of leaves of these plants, respectively. Young leaves were collected from plants exposed to the two light exposure conditions to generate the light and shadow YL and YS, respectively. Flower buds (FB) were collected on plants exposed to high illumination conditions. Adventitious roots (AR) were induced as described in [24] using two-month old stems and were generated over one month. Two YL, YS and OL were collected per plant and each pair was considered as one biological replicate. Ten AR were gathered to generate one biological replicate (5 in total). 

### 2.3. RNA-Sequencing

RNA was extracted from two biological replicates for AR, YL, OL and YS, using TRIzol (Thermo Fisher Scientific, Illkirch-Graffenstaden, France) as previously described [3]. The RNA was subsequently used to prepare libraries with the Illumina TruSeq RNA Library Prep Kit v2. Libraries were sequenced in paired-end mode (2 × 100 bp) by Eurofins Genomics (Les Ulis, France) using the Illumina HiSeq2500 technology. Sequencing data was deposited as project accession number PRJEB40906. EdgeR R package was used to process RNA-seq data. Replicate homogeneity was checked using a multidimensional scaling analysis (Figure S1). It clearly indicated that biological replicates were consistent. The biological coefficient of variation was 0.4 (common dispersion = 0.168531), in the range of typical variation for human data. Spearman rho between similar tissue types was >0.9 (Figure S2). We therefore assumed that in our case, only two biological replicates were sufficient for the purpose of this study.

### 2.4. Generation of a Consensus Transcriptome

Reads were trimmed using FastP [69] version 0.20 with default settings and used to de novo assemble individual transcriptomes using Bridger V2014-12-01 [70]. In order to construct a high quality reference transcriptome suitable for gene expression analysis and biosynthesis gene discovery, all assemblies were merged. Sequencing reads were pseudo aligned onto the merged assembly and counted with Salmon v0.14.1 [71] with bias correction (-biasCorrect) in the variational Bayesian optimized (-vbo) mode to establish the abundance estimates as transcripts per million (TPM). In our compaction procedure we discarded weakly expressed transcripts (>TPM5) and applied a clustering algorithm MMSeqs2 [72] using the following parameters: -mode 1, -c 0.8. Assemblies were evaluated with BUSCO [73] version 3.0.2 compared to existing *V. minor* transcriptomes (Vinca_minor_v1 and Vinca_minor_v3) obtained from the Phytometasyn database (https://bioinformatics.tugraz.at/phytometasyn/). Annotation of the consensus transcriptome was achieved with the Trinotate pipeline [74] version 3.0.1 that uses Blastx and BlastP of TransDecoder [75] V5.3.0 predicted ORFs against Uniprot, and hmmscan [76] against the PFAM database (https://pfam.xfam.org/).

### 2.5. Functional Analysis of the Consensus Transcriptome

Differentially expressed genes (DEGs) were identified by fitting a generalized linear model to each transcript using the edgeR R package [77]. Genes were considered to be differentially expressed if the *p*-value of the exact test on the binomial fit was below 0.01. In this analysis, genes with a log fold change above 2 were considered to be upregulated and genes with a log fold change below -2 were considered to be downregulated. Overlap in DEG sets between different comparisons were visualized by the UpSetR [78] package v1.4.0. Uniprot description keywords were retrieved from the transcriptome annotation and used in the enrichment tests by comparing effectives to a hypergeometric distribution with the phyper function in R [79].

MIA gene conservation was determined through blastn [80] analysis of the *V. minor* consensus transcriptome against known MIA genes from *C. roseus*. Transcripts with the highest bit scores were selected as representatives for each MIA gene equivalent. Graphics were generated with the ggplot2 R package [81].

### 2.6. Heterologous Expression of Tabersonine 16-O-Methyltransferase Candidates in Yeast

Dedicated primers (Table S3) were used to amplify the full coding sequences of T16H, YS1_comp1836_seq0, YS2_comp61_seq0 (Vm16OMT) and Cr16OMT. Primers were designed to include appropriate restriction sites at the extremities of each ORF for subsequent ligation into pESC-His plasmids. Each OMT expressing recombinant vector was cotransformed with the T16H2 expression vector into *S. cerevisiae* strain WAT11 as described in [53]. Transformed yeast strains were grown until the stationary phase in 4 mL of CSM medium (0.67% (*w*/*v*) yeast nitrogen base, 2% (*w*/*v*) dextrose and 0.05% (*w*/*v*) dropout mix without histidine) and harvested by centrifugation. Induction of protein expression was achieved by further culturing the harvested yeasts for 6 h in 10 mL of YPGal medium (2% bacto peptone, 1% yeast extract and 2% Gal) as described by [65].

### 2.7. Recombinant Vm16OMT Production in E. coli, Substrate Specificity Assays and Kinetic Parameters Determination

The Vm16OMT coding sequence was amplified with primers vm16OMTlike-for/vm16OMTlike-rev and cloned into the *BamHI* site of the pRSET-A vector (Thermo Fisher Scientific, Illkirch-Graffenstaden, France) before transformation of *E. coli* BL21(DE3) cells. Transformed cells were cultured until exponential growth (Abs595 nm = 0.5) and protein expression was induced by adding 1 mM IPTG for 4 h at 28 °C. The resulting recombinant protein was purified with a Co2+ matrix according to the manufacturer’s instructions (Talon Resin metal ion, Clontech), concentrated and dialyzed against a phosphate buffer (50 mM pH 7.5) with Amicon® Ultra-4 (30 kDa cut-off, Millipore, Molsheim, France). The purified protein was aliquoted and 10% glycerol added before being flash frozen in liquid nitrogen. Protein concentration and purity were determined by the Bradford assay and SDS-PAGE, respectively.

Enzymatic activity assays were conducted at 30 °C by coincubating 1 µg of each recombinant protein in a final volume of 50 µL potassium phosphate buffer (50 mM pH 7 supplemented with 25 mM of ascorbic acid) with 1 mM S-adenosyl-L-methionine (SAM) and 1 µM of each respective substrate: tabersonine, lochnericine, vincadifformine and 19-hydroxytabersonine. The reactions were stopped with 1 (*v*/*v*) of 100% methanol, centrifuged at 13,000× *g* for 10 min and analyzed by LC–MS. The kinetic parameters were determined under the same conditions by varying substrate concentrations. For 16-hydroxytabersonine kinetics, the alkaloid concentration varied from 0.2 to 100 µM in the presence of a done quantity of SAM. For the SAM kinetics, the concentration of 16-hydroxytabersonine was set and the concentration of SAM varied between 1 and 200 µM. All reactions were performed in four biological replicates. The kinetic parameters were calculated from the enzyme initial activity measured using a non-linear regression following the Michaelis–Menten equation (SigmaPlot12).

### 2.8. UPLC-MS Analyses

UPLC-MS analyses were performed according to [65]. The selected ion monitoring mode was used to collect data for the following compounds: tabersonine (*m*/*z* 337), retention time (RT) = 12.36 min (RT) = 11.3 min; 16-hydroxytabersonine (*m*/*z* 353), RT = 7.9 min; 16-methoxytabersonine (*m*/*z* 367), RT = 12.04 min; 19-hydroxytabersonine (*m*/*z* 353), RT = 6.8 min; vincadifformine (*m*/*z* 339), RT = 11.16 min; lochnericine (*m*/*z* 353), RT = 11.2 min; 16-methoxylochnericine (*m*/*z* 383), RT = 11.4; Ervinceine (*m*/*z* 369) and RT = 11.5 min in Figure 7. Ervinceine was quantified as vincadifformine equivalent.

### 2.9. Gene Expression Measurements Using Real-Time RT-PCR

Expression levels of Vm16OMT were determined by quantitative reverse transcription-PCR according to [65] using dedicated primers compiled in Table S3, ACTIN was chosen as endogenous reference gene, and reverse transcribed RNAs from different *V. minor* organs, including young leaves (YL), old leaves (OL), flower buds (FB) and roots (R). qPCR were performed in triplicate using three biological replicates.

### 2.10. Subcellular Localization Studies

The full length coding sequence of Vm16OMT was amplified using a dedicated primer pair (Table S3) designed to introduce *SpeI* restriction sites to both extremities of the amplicon. The PCR product was subsequently cloned into either *SpeI*- or compatible *NheI* restriction sites of the pSCA-cassette YFPi [82] producing Vm16OMT-YFP and YFP-Vm16OMT fusion proteins, respectively.

For the bimolecular fluorescence complementation (BiFC) assays, the Vm16OMT cDNA was cloned into pSPYCE (MR), pSPYNE173 and pSPYCE (M) plasmids, at the *SpeI* restriction sites of either 5’ or 3’ ends of the coding sequence of the *N*-terminal (YFP^N^, residues 1–173) and *C*-terminal (YFP^C^, residues 156–239) fragments of YFP [83]. This allows expressing the fusion proteins Vm16OMT-YFP^N^, Vm16OMT-YFP^C^ and YFP^C^-Vm16OMT that were used to study protein interactions.

The resulting plasmids were transiently cotransformed with a plasmid expressing the nucleocytoplasm CFP marker [84] into *C. roseus* cells by particle bombardment followed by fluorescence imaging as previously described by [85]. In brief, the *C. roseus* cells were bombarded with DNA-coated gold particles (1 μm) and a 1100-p.s.i. rupture disc at a stopping-screen-to-target distance of 6 cm, using the Bio-Rad PDS1000/He delivery system. After the bombardment, the cells were allowed to recover for 16 - 30 h prior to harvesting and observation. The resulting subcellular localization imaging was performed with an Olympus BX-51 epifluorescence microscope equipped with an Olympus DP-71 digital camera and a combination of YFP and CFP filters. The localization patterns of ± 50 observed cells were finally presented in this study.

### 2.11. Co-Expression Network

For each possible gene pair in the consensus transcriptome, the pairwise Pearson’s correlation coefficient was calculated and subsequently ranked to determine the highest reciprocal ranks (HRR) as described previously [86,87]. The best co-expressed genes were captured for each known MIA pathway gene if their HRR < 100. The resulting associations were visualized using the igraph package in R [88] and communities of the closest co-expressed genes were determined using a fast greedy algorithm [89].

## 3. Results and Discussion

### 3.1. A Consensus and Refined Transcriptome Resource for V. minor

**Figure 2:**
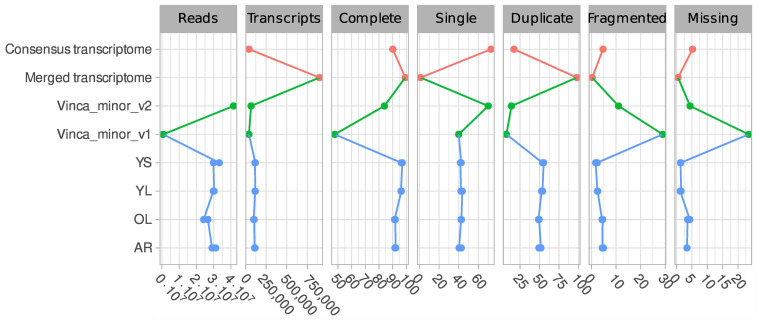
Comparison of *V. minor* transcriptomes in terms of number of generated reads, number of assembled transcripts and BUSCO evaluation scores (% complete, single, duplicated, fragmented and missing). Data sources are depicted as different colors with blue generated in this study, green obtained from previously published data and orange derived from our generated combined transcriptomes.

**Figure 3:**
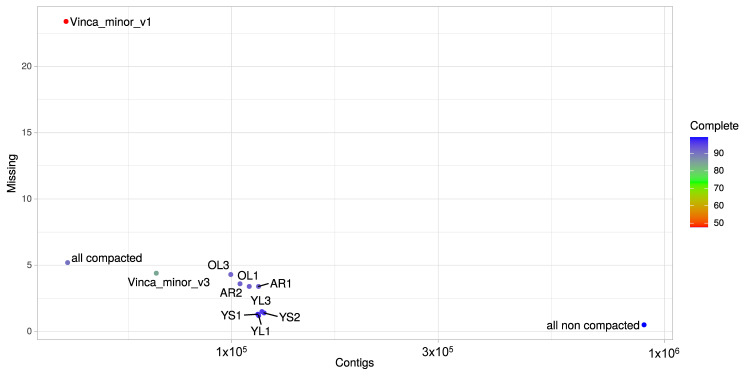
BUSCO evaluation of transcriptome assembly quality. The number of missing BUSCOs is plotted against the total number of contigs. Colors correspond to the percentages of completed BUSCOs. BUSCO analysis was conducted for each individual sample transcriptome for old leaves light (OL), young leaves light (YL), young leaves shadow (YS) and adventitious roots (AR), and their merged transcriptome without (all non compacted) and with compaction (all compacted). BUSCO metrics were compared with available *V. minor* transcriptome resources including the 454 based assembly (Vinca_minor_v1) and the Illumina based assembly (Vinca_minor_v3).

A total of eight libraries (Figure 2) were prepared from RNA extracted from four different tissue types including, mature leaves exposed to light (OL), young leaves exposed to light (YL), young leaves grown under low illumination conditions (YS) and adventitious roots (AR) developing from cut-stems immersed in water. Since the accumulation of many MIAs is stimulated by light, including vindoline in *C. roseus* [54,58], *N*,*β*-d-glucopyranosyl vincosamide in *Psychotria leiocarpa* [90] and camptothecin in *Camptotheca acuminata* [91], we thus exposed *Vinca minor* plants to low and high light exposure conditions (namely shadow and light respectively), in order to explore whether a similar transcriptional regulation could be detected in *V. minor*. Adventitious roots were preferred to native roots as these are more prone to yield high-quality RNA and relevant sequencing results [24]. Illumina sequencing yielded between 24 and 33 million paired-end and processed reads that were used to de novo assemble each sample transcriptome individually using Bridger (Figure 2). All transcriptomes yielded complete BUSCO scores > 91% and missing rates < 5%. BUSCO scores for already published *V. minor* transcriptomes depended on the data type [92]. The 454 based assembly (Vinca_minor_v1, Figure 2 and Figure 3) displayed a high proportion of missing BUSCOs (23.4%) while the Illumina based assembly (Vinca_minor_v3, Figure 2) had a complete BUSCO score of 84% as well as a relatively high fragmentation rate (11%) in contrast to our assemblies (<4% on average; Figure 2).

Next, we aimed at constructing a high-quality reference transcriptome suitable for gene expression analysis and discovery of MIA biosynthetic genes. We thus combined the individual transcriptomes resulting in a merged resource of 896,265 transcripts and a complete BUSCO score of 99.2% (all non compacted, Figure 2). However, this large number of transcripts is far from the closely related species *C. roseus* whose genome was predicted to contain 34,363 genes [93]. Therefore, we aimed to decrease the final merged assembly size by removing redundant sequences without losing biologically relevant transcripts. In our downstream compaction procedures, we discarded weakly expressed genes (TPM < 5) and applied a sequence clustering algorithm (MMSeqs2) [72].

Finally, this procedure allowed a reduction of the merged transcriptome (896,265 transcripts) to a final consensus transcriptome of 41,823 transcripts (all compacted) and an acceptable complete BUSCO score of 90.1% (Figure 2 and Figure 3). This is the first description of a consensus transcriptome resource for *V. minor*. For comparison purposes, genome predictions for *Camptotheca* had a BUSCO score of 93.6% [94] and for *C. roseus* v2 93.3% [93]. Consequently, this *V. minor* consensus transcriptome was used for further analyses.

### 3.2. Functional Annotation of the V. minor Consensus Transcriptome and Differentially Expressed Gene Analysis

**Figure 4:**
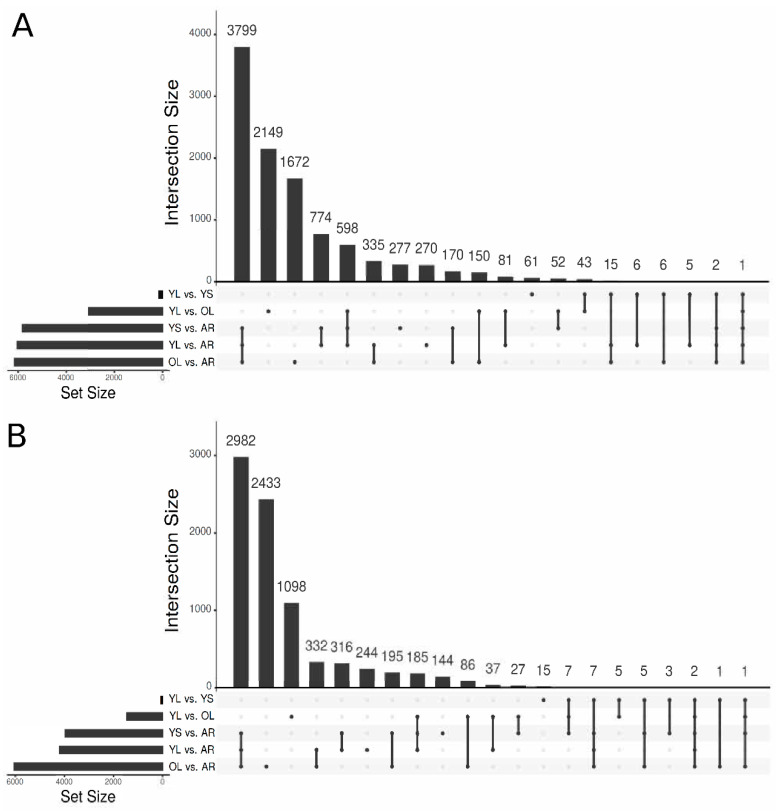
Differential gene expression analysis. UpSetR [78] plots highlighting the intersections resulting from the differentially expressed gene (DGE) analysis for the different comparisons as (**A**) log fold change > 2 indicating increased expression and (**B**) log fold change < −2 indicating decreased expression. Set sizes indicate the total number of transcripts differentially expressed for each comparison.

In Apocynaceae, MIA biosynthetic pathways exhibit complex distributions in different organs and cell-types, and was described in *C. roseus* [95] and predicted in *V. minor* based on MIA distribution [16]. A generalized linear model was thus constructed to detect differentially expressed genes (DEGs) in distinct organs by focusing on five comparisons: YL vs. AR, YS vs. AR, OL vs. AR, YL vs. YS and YL vs. OL (Figure 4). Logically, the most contrasting comparisons opposed aerial organs (YL, YS and OL) to adventitious roots (AR). We found 3799 transcripts significantly more expressed in aerial parts (YS + YL + OL vs. AR; Figure 4A), representing expected Gene Ontology (GO) biological processes, including photosynthesis and responses to light stimulus (Figure S3). In AR, the 2982 specific DEG (Figure 4B) were particularly found to be related to ion transport and homeostasis, oxidative stress, and lignin metabolism (Figure S2). Additionally, we found 2149 transcripts that were exclusively expressed in YL compared to OL (Figure 4A), implying specific metabolic activities (primary or specialized). In YL, a large portion (>100) of transcripts were identified that were related to cell division, mitotic cell cycle phase transition and mitotic cell cycle (Figure S5), revealing intense mitotic activity of YL in contrast to OL. These processes were also found to be increased in AR compared to OL (Figure S6), further emphasizing the active growth of these younger tissues, compared to OL.

The YL vs. YS sample comparison provided insights into the effects of light on young leaves of *V. minor*. Even though this comparison only contained 185 DEG, YL samples did contain genes associated with GO biological pathways (*p*-value < 0.05). Interestingly, light increased the expression of genes related to environmental stresses such as salt stress, heat stress, insects and reactive oxygen species (Figure S7). Furthermore, there was an enrichment of genes related to primary metabolisms, such as the starch catabolic process and lipid transport in YL compared to YS.

Finally, GO keyword enrichment analysis identified 32 transcripts related to alkaloid metabolism that were significantly enriched in YL compared to AR, and 19 alkaloid metabolism related transcripts significantly enriched (*p*-value < 0.05) in AR compared to YL (Figure S8). Furthermore, >20 transcripts with GO enrichment terms related to the alkaloid metabolism were identified in AR, compared to OL (Figure S9), and YL had more than double the number of transcripts enriched in alkaloid metabolism (20) compared to OL (Figure S10), indicating that alkaloid metabolism may be favored in these younger tissues. Such differences in alkaloid metabolism gene expression thus confirm previous results indicating that activities of MIA biosynthetic enzymes are higher in young tissues [13,14,15]. This contrasted expression also suggests that gene expression networks could result in new MIA gene identification.

### 3.3. Identification of Candidate Genes from the MIA Pathway through Homology-Based Predictions

**Figure 5:**
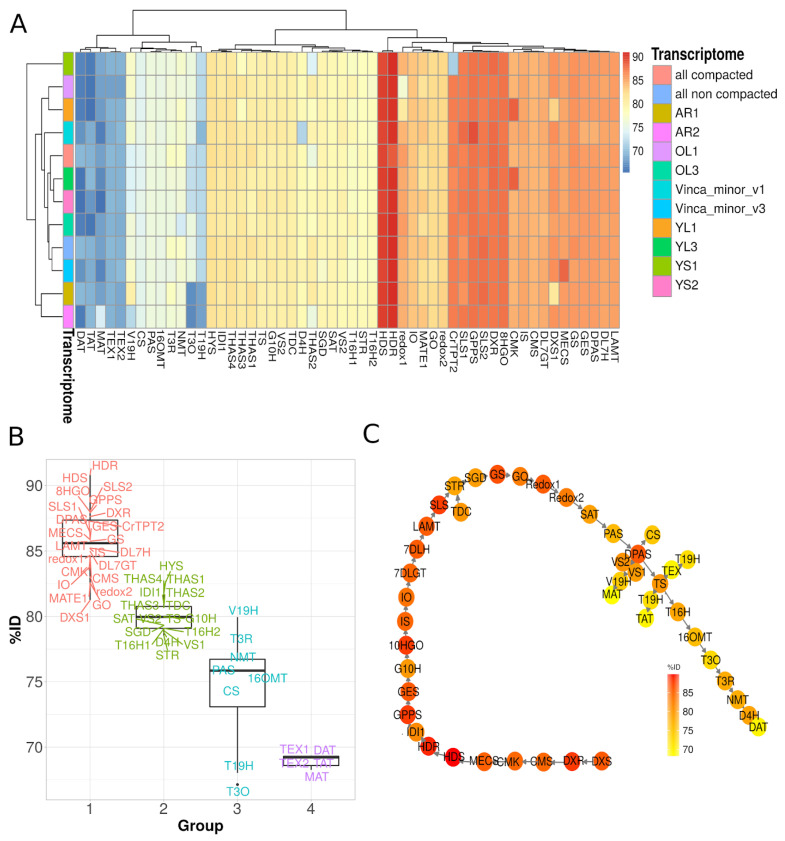
Comparison of *V. minor* transcripts to *C. roseus* reference sequences from the MIA pathway. (**A**) Percent of identity was calculated with blastn for each *V. minor* assembly against *C. roseus* MIA nucleotide sequences. (**B**) Average %ID per group of genes in the *V. minor* consensus assembly. (**C**) Comparison of *V. minor* consensus assembly homologs to the elucidated *C. roseus* vinblastine pathway.

The MIA pathway in *C. roseus* is now well described and shares high similarity with that of *V. minor* for all common steps up to vincadifformine or tabersonine, for instance. We thus conducted a homology-based identification of MIA genes by analyzing *C. roseus* gene conservation in our *V. minor* assemblies through blastn analysis followed by selecting the best Bit scoring transcripts for each hit. For all known MIA genes from *C. roseus* encoding biosynthetic enzymes, transporters and transcription factors, the best hits from our *V. minor* assembly were identified and listed in Table S1. Overall, MIA gene reconstruction was similar in each assembly but in some cases % identity was found to be better in the compacted *V. minor* resources (Figure 5A). Most single transcriptomes had at least one MIA hit with lower sequence identity than the transcript equivalent captured in the consensus assembly, as for example, TPT2 (YS1 samples), redox1 (OL1 samples), deoxyxylulose-5-phosphate synthase 1 (DXS1) from the MEP pathway (YL1 and AR1) and T3O (YL3, YS2, AR1 and AR2 samples). By combining different transcriptomes from different tissue types, we can anticipate that the best transcripts from each set will be captured. Interestingly, CMK in YL1 and YL3 had higher % amino acid sequence identity than the transcript captured in the consensus transcriptome (Figure 5A). However, upon further investigation it was found that although both transcripts were captured in the all non compacted transcriptome, they did not display the highest bit-scoring transcripts in this assembly and were thus discarded. Furthermore, these transcripts had a lower alignment length (<100 bp) to the best hit transcript (>1400 bp). This case study further demonstrated the potential of combining different transcriptomes in order to rationally increase the chances of identifying true gene homologs.

Upon further exploration of our consensus transcriptome, we found four groups of gene sequence conservation compared to *C. roseus* (Figure 5B,C). Each group corresponds to a specific subpart of the pathway, thus highlighting a modular conservation of this pathway in *V. minor*. The highest gene conservation (>85% in average) was observed for genes from early biosynthetic steps including MEP and monoterpene secoiridoid branches, such as GES, IS, IO, DL7GT, DL7H, LAMT up to SLS. Interestingly, genes related to Strychnos-type MIAs (GS and GO) together with redox1 and redox2 (Figure 1) were also part of this group, suggesting a low sequence evolution permissiveness towards the encoded enzymes. Surprisingly, while their functions are well-conserved, we noted that TDC, STR and SGD belong to the second group of sequence conservation (<80% in average), which could result in slightly different catalytic properties. This group also includes genes encoding alcohol dehydrogenases involved in tetrahydroalstonine and other heteroyohimbine MIA synthesis (Figure 5B). Such lower conservation of tetrahydroalstonine synthase (THAS) was quite expected since tetrahydroalstonine has not been detected to date in *V. minor*. However, these genes may encode enzymes involved in the synthesis of other non-described MIAs from *V. minor*. In addition, genes of the four last steps of tabersonine synthesis (SAT, PAS, DPAS and TS) also display lower identities, similarly to those observed for TDC, STR and SGD. This second group also includes vincadifformine synthase (VS1 and VS2) and orthologs of T16H1 and T16H2 that may catalyze the hydroxylation of tabersonine and/or vincadifformine. It thus gathers many orthologs of genes involved in the central steps of MIA biosynthesis, mainly synthesis and first decorations of strictosidine. The third group of gene conservation (around 75% identity) contains less orthologs, mostly related to putative 19-hydroxylation of tabersonine (V19H and T19H), vindoline (16OMT, T3R and NMT) and catharanthine (CS) synthases. Once again, these results were expected since no similar reactions have been described to date in *V. minor* but these genes likely correspond to candidates of interest for the synthesis of other and/or related MIAs. However, orthologs of T3O also belong to this group and may constitute potential candidates for the cyclization reaction of vincamine synthesis [28]. The fourth and last group of identity (below 70% of identity) mainly encompasses orthologues of lochnericine synthases (TEX1 and TEX2) that were unexpectedly quite distant from the *C. roseus* counterparts, and orthologues of the acyltransferases DAT, TAT and MAT. While no acetylated version of tabersonine has been described to date, these acyltransferases may be engaged in the synthesis of other acetylated MIA such as acetylated vincarubine [96]. Finally, for six of the candidate genes, we validated their expression levels quantified by RNA-seq by quantitative PCR performed on three different biological samples (correlation 0.785, *p*-value = 5.701e^−06^, Pearson CC), thus suggesting that gene co-expression analysis could be conducted to guide prediction/identification of MIA biosynthetic genes (Figure S11). In conclusion, analyzing our consensus transcriptome through homology-based searching resulted in an exhaustive identification of potential MIA biosynthetic genes in *V. minor*. Furthermore, it highlighted differential degrees of gene sequence conservation that might result from the intrinsic capacities of enzymes to evolve without losing initial activity and to acquire new or altered catalytic properties [97,98].

### 3.4. Identification and Functional Validation of a V. minor Vincadifformine/Tabersonine 16-O-Methyltransferase

**Figure 6:**
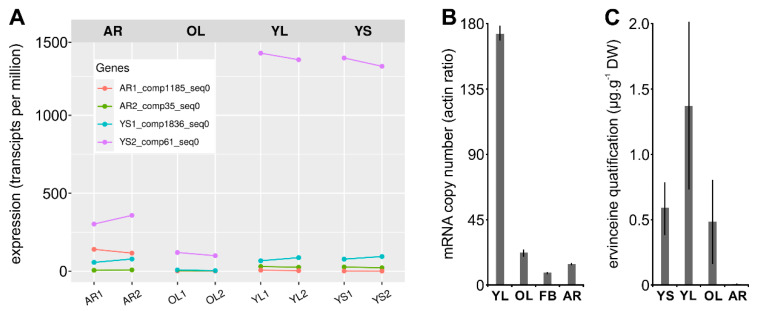
Relative transcript quantification of candidate tabersonine 16-*O*-methyltransferases and ervinceine amount measurement. (**A**) Relative expression was quantified as transcripts per million (TPM) after mapping RNA-seq reads from young leaves light (YL), young leaves shadow (YS), old leaves light (OL) and adventitious roots (AR) to the consensus transcriptome. (**B**) Relative YS2_comp61_seq0 quantification by reverse transcription quantitative PCR (qPCR) using RNA extracted from young leaves (YL), old leaves (OL), flower bud (FB) and roots (R). Data represents means ± SD of replicates performed on three distinct biological samples of each organ type. (**C**) Ervinceine quantification as µg/mg (mean ± SD, *n* = 3) in adventitious roots (AR), old leaves light (OL), young leaves light (YL) and young leaves shadow (YS) performed by UPLC–MS analysis.

To determine the reliability of our annotated consensus transcriptome in identifying candidate MIA biosynthetic pathway genes, we performed the functional validation of one of the predicted candidates. We thus chose one from the third group of gene conservation to characterize a candidate with a relatively low identity (75% in average) to strengthen the approach. More specifically, we focused on identifying an ortholog of 16OMT that catalyzes the methoxylation of 16-hydroxytabersonine in *C. roseus* leaves while it is notably proposed to methylate 16-hydroxyvincadifformine to produce ervinceine in *V. minor* (Figure 1). By mining our *V. minor* consensus transcriptome, we identified four tabersonine 16-*O*-methyltransferase-like candidates (Table S1) namely AR2_comp35_seq0, YS2_comp61_seq0, AR1_comp1185_seq0 and YS1_comp1836_seq0, by decreasing identity. All candidates display nucleotide sequence similarity (>70%) to the *C. roseus* 16OMT. The deduced protein sequences of all four candidates maintained *O*-methyltransferase domains (PF00891.15, Figure S12), as determined by hidden-Markov comparison against the PFAM database. Interestingly, the relative expression levels (as transcripts per million; TPM) of each candidate, obtained by mapping the reads from each tissue type to the consensus transcriptomes (Figure 6A), revealed that YS2_comp61_seq0 display the highest expression in all the organs tested with a maximum in the young aerial parts of the plant (YL and YS, >1300 TPM). This high level of expression was confirmed by quantitative RT-PCR providing additional evidence of high expression in YL (Figure 6B). By contrast, the three remaining candidates were barely detectable in old leaves and roots. Such differences in gene expression were of interest to prioritize candidate analysis given the specificity of MIA accumulation, notably ervinceine, in YL (Figure 6C).

**Figure 7:**
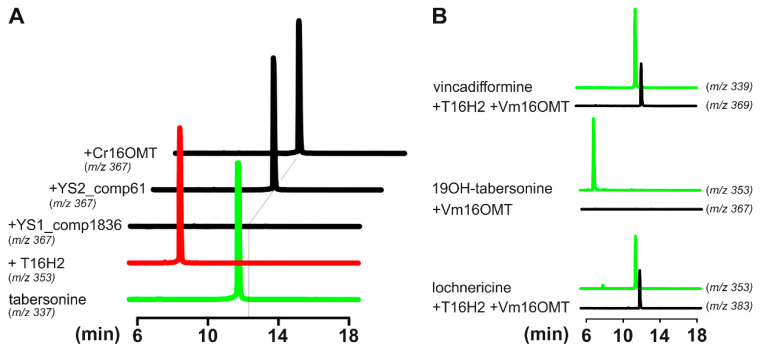
Screening selected candidates for tabersonine 16-*O*-methyltransferase activity. (**A**) Galactose induced yeast cell cultures co-expressing T16H2 (red line) with selected 16OMT-like candidates (YS1_comp1836_seq0 and YS2_comp61_seq0) and positive control Cr16OMT (black lines), incubated with tabersonine (green line, *m*/*z* 337). Selected ion monitoring and comparisons to authentic standards were used to detect the resulting hydroxylated (*m*/*z* 353) and methylated (*m*/*z* 367) tabersonine products by LC–MS analysis. The grey line highlights 16-methoxytabersonine retention time. (**B**) Substrate specificity of recombinant Vm16OMT in combination with recombinant T16H2 was determined by coincubation with 19OH-tabersonine, lochnericine and vincadifformine (green lines), forming methylated products that were detected by UPLC–MS (black lines). No methylated product was observed after incubation of recombinant Vm16OMT with 19-hydroxytabersonine.

Based on ervinceine accumulation and gene expression profiles (e.g., high expression in young leaves), we thus selected YS1_comp1836_seq0 and YS2_comp61_seq0 for further functional validation. Given 16OMT substrate promiscuity and its capacity to methylate distinct tabersonine derivatives [15,24], both candidates were first tested for tabersonine 16-*O*-methyltransferase activity by co-expression in yeast with T16H2. After cloning each coding sequence in pESC-HIS, yeasts were cotransformed with the corresponding plasmid together with pESC-LEU T16H2 [53]. As a control, yeast strains expressing either T16H2 or T16H2 and 16OMT from *C. roseus* were also included in the assay as previously described [24]. The resulting yeasts were then cultured, induced with galactose and fed tabersonine (125 µM) for 24 h, before analysis of the resulting products using ultra-performance liquid chromatography–mass spectrometry (UPLC–MS; Figure 7A). Comparisons of selected ions (*m*/*z* 337 for tabersonine, *m*/*z* 353 for 16-hydroxytabersonine and *m*/*z* 367 for 16-methoxytabersonine) to the retention times of authentic standards allowed for the identification of the enzymatic reaction products. While yeasts expressing T16H2, T16H2 and 16OMT converted tabersonine into 16-hydroxytabersonine and 16-methoxytabersonine, respectively, we observed that only the YS2_comp61_seq0 candidate co-expressed with T16H2 consumed tabersonine and produced a compound with *m*/*z*, retention time and UV spectrum similar to 16-methoxytabersonine (Figure S13). This result thus confirms that YS2_comp61_seq0 displays tabersonine 16-*O*-methyltransferase activity and was subsequently named *V. minor* tabersonine 16-*O*-methyltransferase (Vm16OMT, GenBank accession no. MH010798). Above all, it confirmed the reliability of our annotated consensus transcriptome and predictions of MIA candidate genes in *V. minor*.

### 3.5. Estimation of Vm16OMT Substrate Specificity

To determine the enzymatic substrate specificity of Vm16OMT in vitro, yeast strains expressing T16H2 and Vm16OMT (YS2_comp61_seq0) or Vm16OMT alone were fed with different substrates including vincadifformine/lochnericine and 19-hydroxytabersonine, respectively (Figure 7B). The resulting products were analyzed by UPLC–MS, which detected methylated products eluting at *m*/*z* 367 following tabersonine incubation, *m*/*z* 383 following lochnericine incubation and *m*/*z* 369 following vincadifformine incubation. As previously observed for *C. roseus* 16OMT, we concluded that Vm16OMT methylated the 16-hydroxylated tabersonine related compounds including 16-hydroxyvincadifformine and 16-hydroxylochnericine besides 16-hydroxytabersonine (Figure 7A,B). By contrast, no methylation of 19-hydroxytabersonine was observed. These results thus indicated that Vm16OMT can accept a variety of aspidosperma substrates but is regioselective towards the 16-position hydroxyl group as a methyl acceptor. The promiscuity of Vm16OMT towards a variety of 16-hydroxylated aspidosperma compounds further suggests that this enzyme may catalyze additional biosynthetic reactions in *V. minor*.

**Figure 8:**
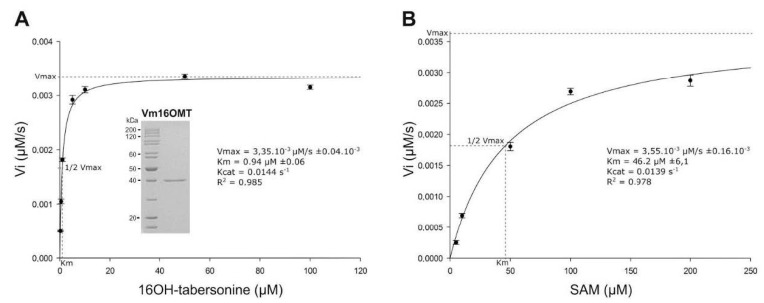
Determination of Vm16OMT kinetic parameters for 16-hydroxytabersonine (**A**) and SAM (**B**). Vm16OMT initial velocities in 16-methoxytabersonine synthesis were measured using fixed SAM concentration and from 0.2 to 100 µM 16-hydroxytabersonine (**A**) or fixed 16-hydroxytabersonine concentration and from 1 to 200 µM SAM (**B**). Nonlinear regressions based on the Michaelis–Menten equation were applied to estimate kinetic parameters. Purity of the recombinant Vm16OMT used was controlled on acrylamide gel stained with Coomassie blue (**A**) Error bars correspond to standard deviation of biological replicates (*n* = 4).

In order to gain insight into Vm16OMT enzymatic properties, its biochemical parameters were subsequently evaluated following heterologous expression in *Escherichia coli* and purification (Figure 8A; inset). The substrate specificity was determined by coincubating the recombinant Vm16OMT with 16-hydroxytabersonine as a methyl acceptor and S-adenosyl-L-methionine (SAM) as a methyl donor. Under these conditions, substrate saturation kinetics revealed that the recombinant Vm16OMT exhibited a high affinity for 16-hydroxytabersonine (Km 0.94 ± 0.06 µM; Vmax 3.35 × 10^−3^ ± 0.04 × 10^−3^ µM·s^−1^; kcat 0.0144 s^−1^) and SAM (Km 46.2 ± 6.1 µM; Vmax 3.55 × 10^−3^ ± 0.16 × 10^−3^ µM·s^−1^; kcat 0.039 s^−1^; Figure 8A,B), which remains in the same order of magnitude as Cr16OMT parameters [15]. This suggests that both OMTs share similar catalytic properties to ensure the synthesis of related MIAs in *C. roseus* and *V. minor*.

### 3.6. Subcellular Localization of Vm16OMT

**Figure 9:**
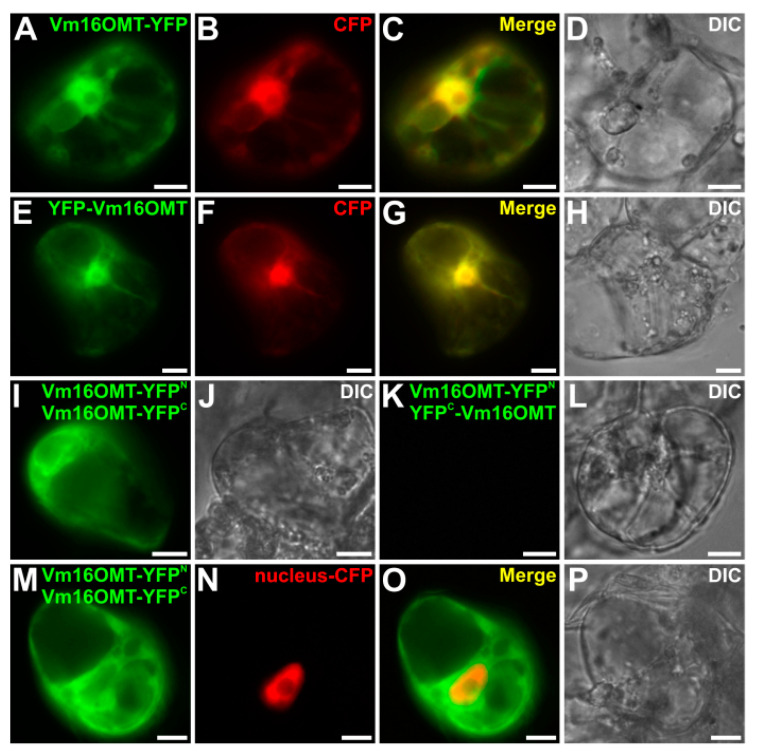
Subcellular localization of Vm16OMT in *C. roseus* cells. Vectors containing Vm16OMT-YFP (**A**–**D**) and YFP-Vm16OMT (**E**–**H**) were transiently co-expressed in *C. roseus* cells with a nucleocytoplasmic (CFP) marker. Bimolecular fluorescence complementation (BiFC) analyses (**I**–**P**) were performed by transiently co-expressing the corresponding constructs described in the upper right of each panel. A nuclear-CFP marker was included (**N**). Colocalization of the YFP and CFP fluorescence signals appear in the merged image. Cell morphology was observed using differential interference contrast (DIC). Bar 10 µm.

While the cellular and subcellular organization of the MIA pathway has been widely characterized in *C. roseus* [19,95], only little is known about the corresponding architecture in *V. minor*. To gain insight into this organization and to complete the characterization of Vm16OMT, we studied the subcellular localization of Vm16OMT through YFP imaging analysis. The full-length Vm16OMT ORF was cloned at either 5’-end or 3’-end of YFP coding sequence in the pSCA-YFP plasmid to generate the Vm16OMT-YFP or YFP-Vm16OMT fusion proteins, respectively. Both constructs were transiently expressed in *C. roseus* cells in combination with a cyan fluorescent protein (CFP) nucleocytosolic marker (Figure 9A–H). For both orientations of fusion, a similar signal of fluorescence was observed, perfectly superimposed with that of the CFP marker. This suggests that Vm16OMT is mainly located in the cytosol and may undergo a passive diffusion into the nucleus due to its small size since no nuclear localization signal could be detected in Vm16OMT primary sequence. In addition, the quaternary structure of Vm16OMT was further analyzed through bimolecular fluorescence complementation (BiFC) assays. In this case, the Vm16OMT ORF was cloned at the 5’-end of the coding sequence of the split-YFP^N^ fragment (Vm16OMT-YFP^N^) and either at the 5’- or at the 3’-end of the coding sequence of the split-YFP^C^ fragment to generate Vm16OMT-YFP^C^ and YFP^C^-Vm16OMT, respectively. The two different combinations of constructs (Vm16OMT-YFP^N^ and Vm16OMT-YFP^C^; Vm16OMT-YFP^N^ and YFP^C^-Vm16OMT) were transiently expressed in *C. roseus* cells to analyze the reconstitution of the BiFC complex through the apparition resulting of a YFP fluorescent signal (Figure 9I–L). Such formation was observed for the Vm16OMT-YFP^N^/Vm16OMT-YFP^C^ combination while no BiFC complexes were reformed with the Vm16OMT-YFP^N^/YFP^C^–Vm16OMT combination. These results thus suggest that Vm16OMT is engaged in homodimer or homomultimer formation through a head-to-head association. This was in agreement with the presence of a dimerization domain (PF08100.8, Figure S12) located at the *N*-terminal end of plant *O*-methyltransferases [99]. Interestingly, we also noticed that Vm16OMT–BiFC complexes were localized exclusively in the cytosol and not in the nucleus as revealed by the lack of superimposition of the BiFC YFP signal with that of the nucleus CFP marker (Figure 9M–P). This nuclear exclusion probably results from the increase of Vm16OMT size due to homodimer formation (around 80 kDa) that may exceed nuclear pore exclusion size that is around 60 kDa [100]. Such restricted distribution to the cytosol has been already observed for *C. roseus* 16OMT and may favor captation of 16-hydroxytabersonine released by the endoplasmic reticulum-anchored T16H2 to increase MIA biosynthetic flux [53,84]. A similar hypothesis can be made for Vm16OMT cytosolic localization.

### 3.7. Gene Co-Expression Analysis

**Figure 10:**
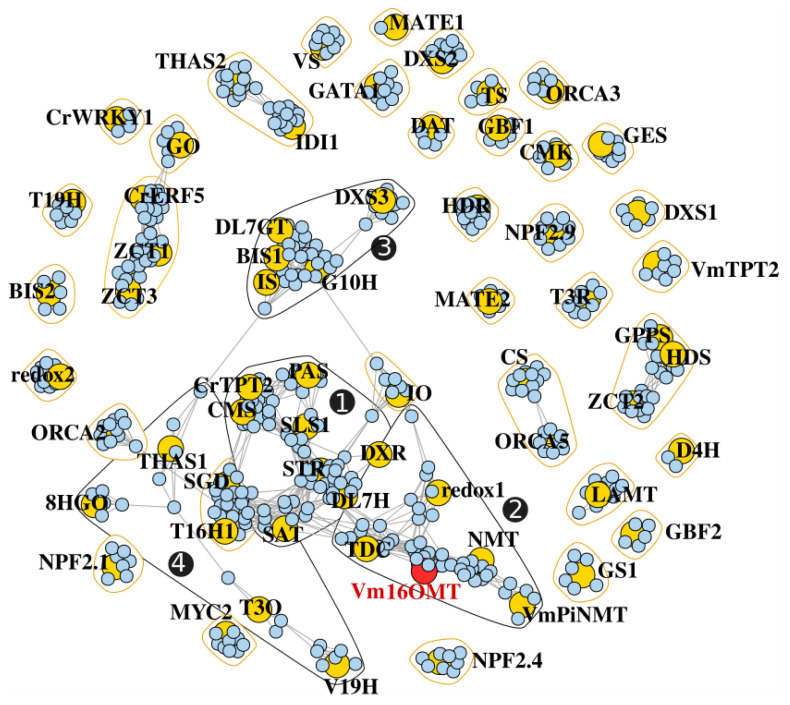
Co-expression network depicting the transcriptional relationship between annotated MIA genes (yellow) and *V. minor* transcripts (blue). Connected genes are indicated by grey edges for the retained HRR. Polygons correspond to communities detected using a fast greedy modularity optimization algorithm. The four main communities in the largest network are numbered with black circles and black polygons.

Finally, after validating our *V. minor* consensus transcriptome for homology-based identification of MIA genes, we took advantage of the multiple samples from the dataset to initiate the prediction of candidate genes catalyzing unknown MIA biosynthetic steps. Usually, genes sharing similar functions or involved in similar pathways display similar expression profiles [64]. Such similarities can be exploited in a “Guilt-By-Association” analysis to guide the identification of additional MIA pathway genes in the dataset [101]. For example, the downstream tabersonine pathway genes TEX and TAT were identified in *C. roseus* by searching candidates that were highly co-expressed with T16H [23,24]. Additionally, a co-expression analysis was used to short list candidates for subsequent *O*-methyltransferase activity assays towards identifying the 10-hydroxycamptothecin *O*-methyltransferase (Ca10OMT) in *C. acuminata* [102]. Therefore, we conducted a co-expression analysis with the sequencing data generated from the four different tissue types (YL, YS, OL and AR). The predicted *V. minor* orthologs of MIA pathway genes from *C. roseus*, including the newly characterized Vm16OMT sequence, were used in the pathway level correlation (PLC) using pairwise Pearson’s correlation coefficients (PCCs), ranked according to the highest reciprocal ranking (HRR) [87]. This analysis identified 39 co-expressed communities (Table S2). The four largest communities were found to be connected within a single large main network (Figure 10, communities 1, 2, 3 and 4).

The largest group (community 1) revealed 63 transcripts upregulated with genes related to the seco-iridoid branch (CMS, DL7H, DXR and SLS) and STR, which catalyzes strictosidine biosynthesis in the first committed step towards MIA metabolism. This group also contained the downstream iboga- and aspidosperma-type MIA pathway genes responsible for the sequential conversion of stemmadenine into the central biosynthetic precursor, *O*-stemmadenine acetate (SAT and PAS). One of the co-expressed transcripts was predicted to encode a putative alcohol dehydrogenase and four were predicted to encode putative cytochrome P450s (Table S2).

The Vm16OMT was positioned within the second largest group (community 2) containing homologs to genes from the tabersonine/vincadifformine (redox1) and downstream vindoline pathways (NMT), co-expressed with a DXS homolog from the MEP pathway. Interestingly, an additional *O*-methyltransferase-like transcript was identified within this community, and two alcohol dehydrogenase-like transcripts (Table S2). Alcohol dehydrogenases and cytochrome P450’s have long been implicated in MIA biosynthetic pathways where they catalyze numerous oxidation and reduction rearrangements of central precursor molecules, forming new types of scaffolds and decorations [46,47,48,103]. *V. minor* produces an assortment of MIA’s containing such decorations and rearrangements, potentially resulting from these identified candidates. For example, vincorine/norvincorine displays a methoxy on C15 [104], which probably involves a cytochrome P450 for hydroxylation, together with vincamine and ervinceine that both contain C17 and C16 hydroxyl groups, respectively [7,105]. Some of the associated P450 may thus constitute possible candidates for these unknown reactions.

The third group (community 3) contained genes related to the iridoid pathway (DL7GT, G10H and IS) co-expressed with DXR from the upstream MEP pathway, and the basic helix-loop-helix (bHLH) transcription factor, BIS2. BIS2 specifically regulates the iridoid branch of the pathway [106,107]. Capturing BIS2 tightly co-expressed with iridoid pathway genes thus added confidence to the network topology. However, no putative candidates can be identified from this third community and from the fourth one. This may illustrate that genes encoding enzymes from other branches of the pathway show a lower level of gene correlation. In agreement with this hypothesis, we identified orthologues of T16H and 2-hydroxyisoflavanone dehydratase in the fifth community that contains only nine correlated genes and no already known genes from the MIA pathway. In conclusion, our “Guilt-By-Association” analysis resulted in the identification of new candidate genes that can guide the future discovery of enzymes catalyzing hitherto unknown steps of MIA biosynthesis in *V. minor.*

## 4. Conclusions

In this study, we present a comprehensive and optimized *V. minor* consensus transcriptome compared to previous versions. This resource was used to predict MIA biosynthetic genes through a homology-based identification validated by the characterization of Vm16OMT. Gene correlation studies also resulted in the prediction of candidate genes putatively involved in unknown reactions of the pathway. As such, our work illustrates how gene discovery might be accelerated by using guilt-by-association studies together with the traditional homology-based approach. This also provides additional layers of evidence to aid in prioritizing MIA candidate genes before testing, and to guide detection of candidates that could have been missed due to low identity to known genes. In fact, it has already been shown that the closest gene homologs do not necessarily always correspond to true active candidates [93], highlighting the necessity for additional transcriptomic mining approaches for identification and prioritization of candidate genes. Based on this new resource, we can anticipate that the biosynthetic routes of vincamine and other less valuable MIAs will be elucidated in the near future. It will thus open new perspectives towards the development of microbial cell factories expressing the corresponding biosynthetic pathways for implementing an alternative supply of valuable MIAs.

## Supplementary Materials

The following are available online at https://www.mdpi.com/2218-273X/10/12/1595/s1, Figure S1. Multidimensional scaling analysis of the RNA-seq experiments. Samples are projected on the two first dimensions according to their gene expression profiles. Closeness between samples on the grid indicates a strong similarity. Figure S2. Similar tissue types are strongly correlated. Spearman’s rho correlation was measured to check biological replicate homogeneity. Higher rho values indicate stronger similarities between pairs of samples. Figure S3: DEG with logFC > 2 mapped to GO biological pathways that are significantly enriched (*p* < 0.05). Figure S4: DEG with logFC < 2 mapped GO biological pathways that are significantly enriched (*p* < 0.05). Figure S5: Significantly enriched GO biological pathways (*p* < 0.05) in the upregulated (log fold change > 2) and downregulated (log fold change < 2) YL vs. OL DEG comparison. Figure S6: Significantly enriched GO biological pathways (*p* < 0.05) in the upregulated (log fold change > 2) and downregulated (log fold change < 2) OL vs. AR DGE comparison. Figure S7: Significantly enriched GO biological pathways (*p* < 0.05) in the upregulated (log fold change > 2) and downregulated (log fold change < 2) YL vs. YS DEG comparison. Figure S8: Significantly enriched GO biological pathways (*p* < 0.05) in the upregulated (log fold change > 2) and downregulated (log fold change < 2) YL vs. AR DEG. Figure S9: Significantly enriched GO keywords (*p* < 0.05) in the upregulated (log fold change > 2) and downregulated (log fold change > 2) OL vs. AR DEG comparison. Figure S10: Significantly enriched GO keywords (*p* < 0.05) in the upregulated (log fold change > 2) and downregulated (log fold change < 2) YL vs. OL DEG comparison. Figure S11. Comparison of expression levels (log2) obtained by qPCR and RNA-seq (transcripts per million) for candidate genes. A linear regression was used to model RNA-seq expression levels (expressed in transcripts per million) from qPCR values (normalized to actin raw expression). The blue line represents the regression line with a 95% confidence interval shown by the grey area. Figure S12: Alignment of the amino acid sequences of 16-*O*-tabersonine methyltransferase candidates. Sequence identity and similarity are highlighted by black and grey shading, respectively. The dimerization domain is highlighted in red and the *O*-methyltransferase domain is highlighted in blue. Figure S13: UPLC-DAD-MS analysis of 16-methoxytabersonine. (A) MS spectrum and (B) UV spectrum. Table S1: Blastn analysis of *V. minor* consensus transcriptome against elucidated *C. roseus* MIA genes, MIA transporters and MIA transcription factors. GenBank accession numbers of *C. roseus* genes are displayed in brackets. Table S2: Transcripts that were co-expressed with MIA genes listed and arranged in communities that were determined by a fast greedy algorithm. Transcripts were annotated using blastx against the UniProt database. Table S3: Primers used in this study.
