# Supplementary material for: Identifying Genes Involved in Alkaloid Biosynthesis in Vinca minor through Transcriptomics and Gene Co-Expression Analysis
Source: Biomolecules. 2020 Nov 24;10(12):1595. doi: 10.3390/biom10121595 (PMC7761029; doi:10.3390/biom10121595)
Supplement: Supplementary file 1 [file biomolecules-10-01595-s001.pdf]

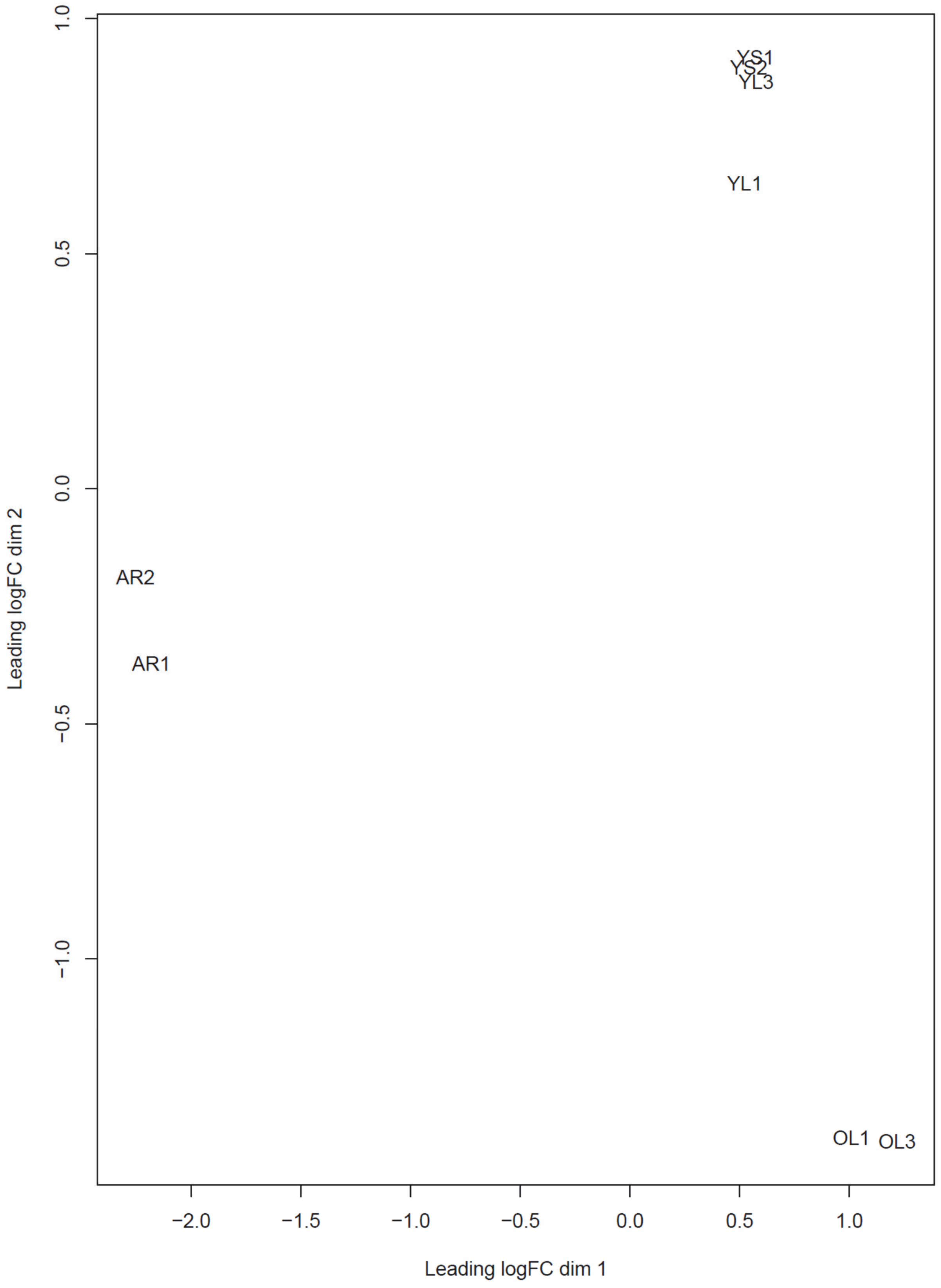

Figure S1. Multidimensional scaling analysis of the RNA-seq experiments. Samples are projected on the two first dimensions according to their gene expression profiles. Closeness between samples on the grid indicates a strong similarity.

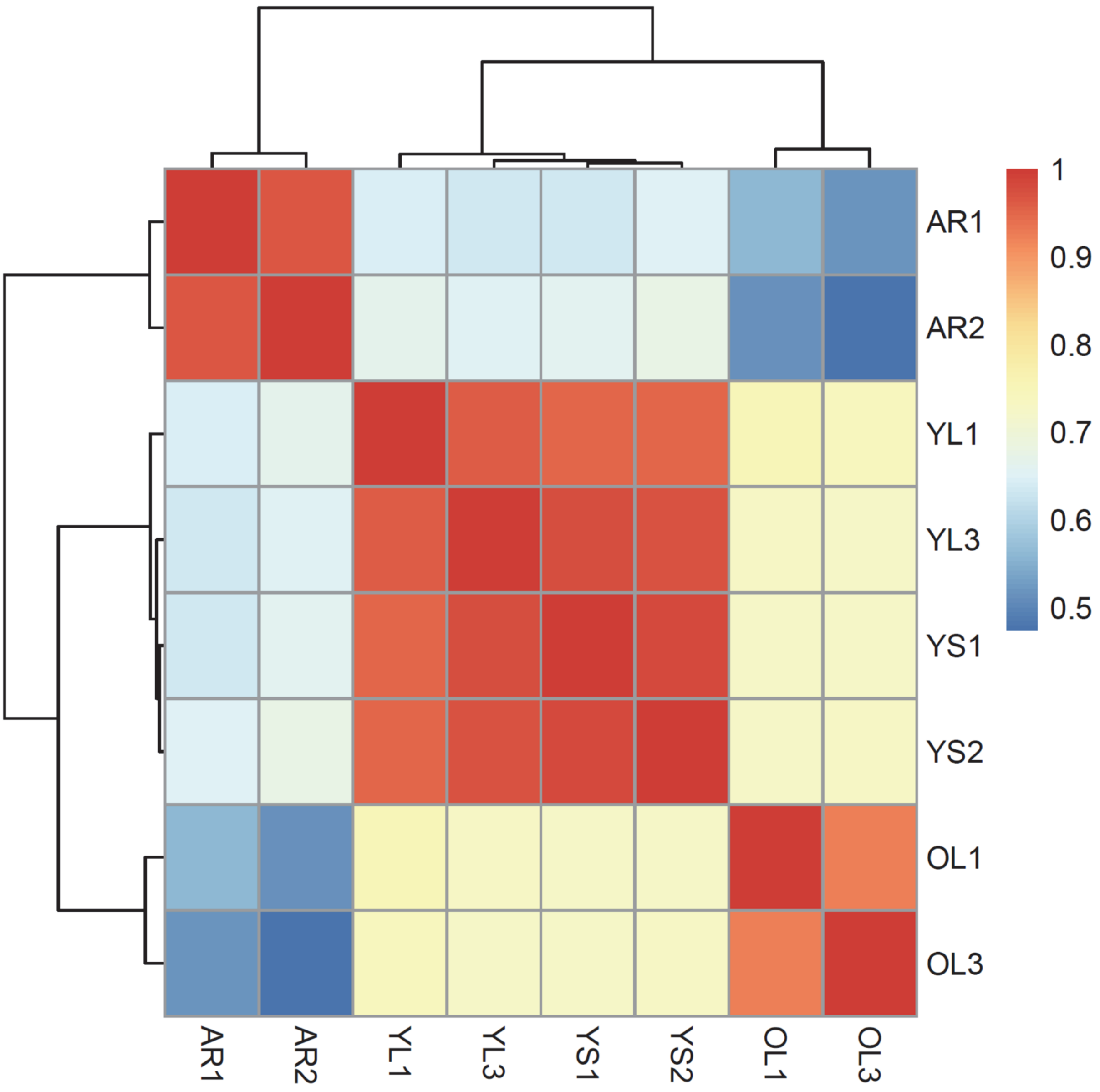

Figure S2. Similar tissue type are strongly correlated. Spearman's rho correlation was measured to check biological replicate homogeneity. Higher rho values indicate stronger similarities between pairs of samples.

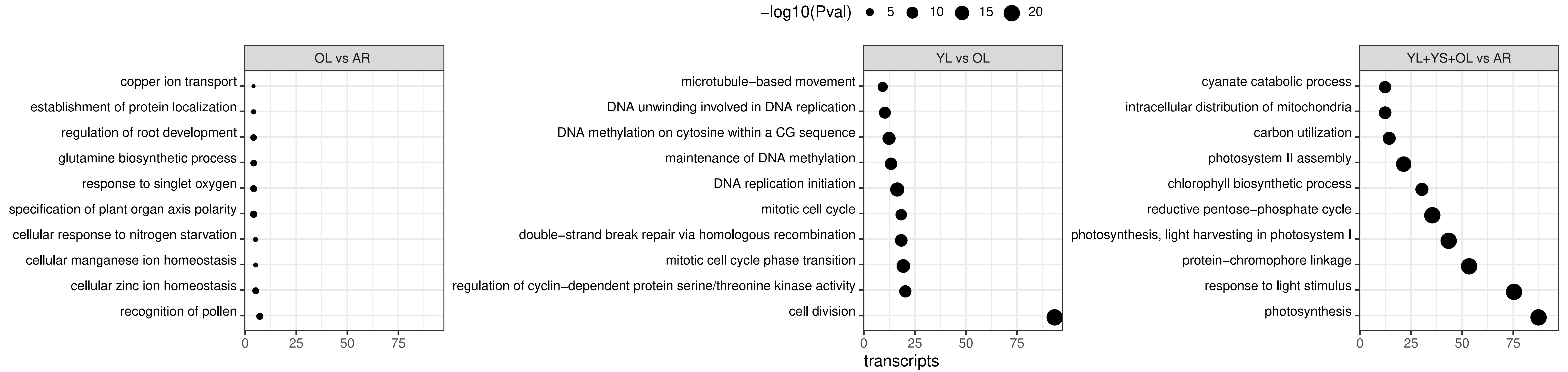

Figure S3. DEG with logFC > 2 mapped to GO biological pathways that are significantly enriched ($p<0.05$)

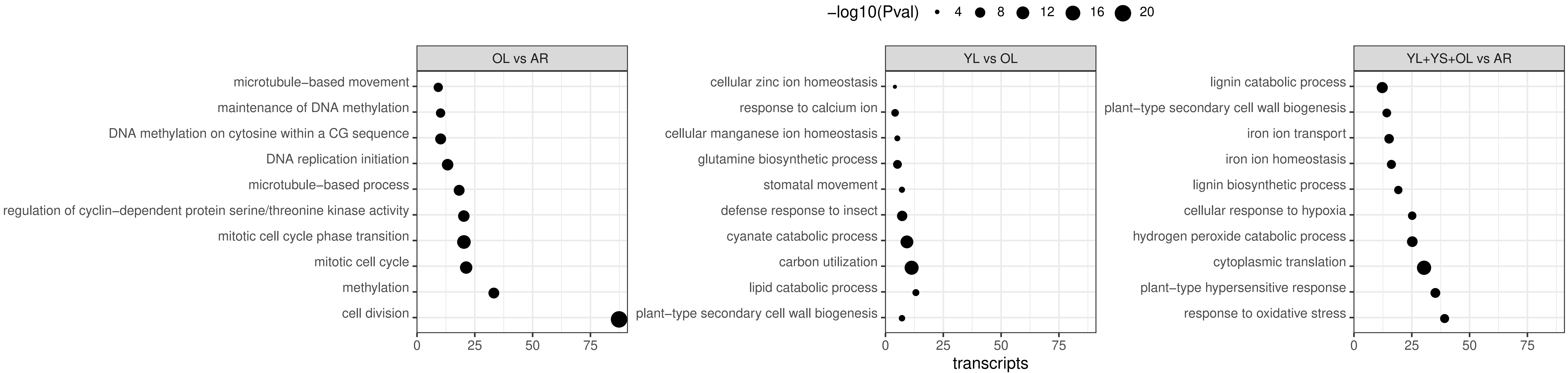

Figure S4. DEG with logFC < 2 mapped GO biological pathways that are significantly enriched (p<0.05).

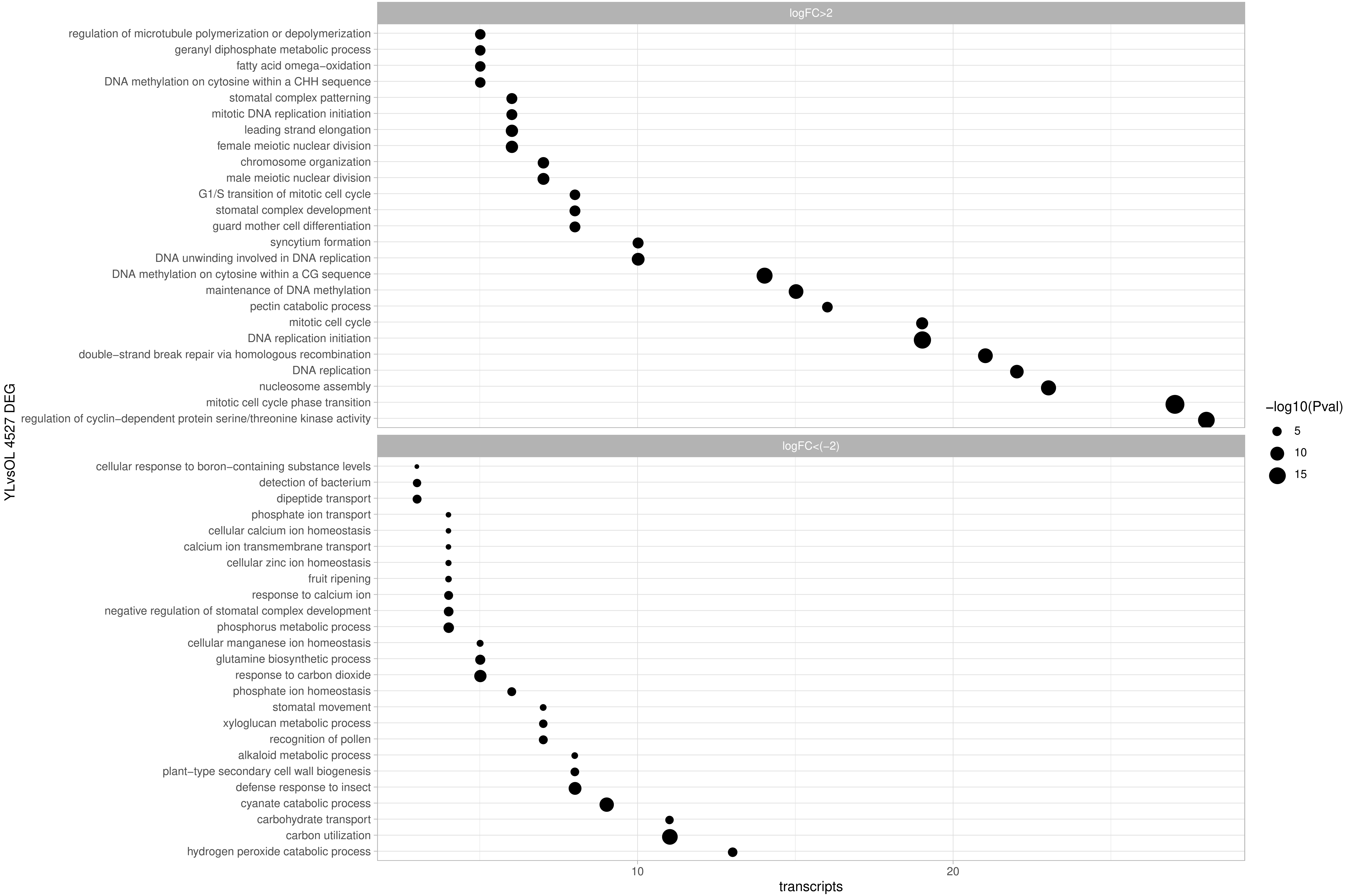

Figure S5. Significantly enriched GO biological pathways (p<0.05) in the upregulated (log fold change > 2) and downregulated (log fold change <2) YLvsOL DEG comparison.

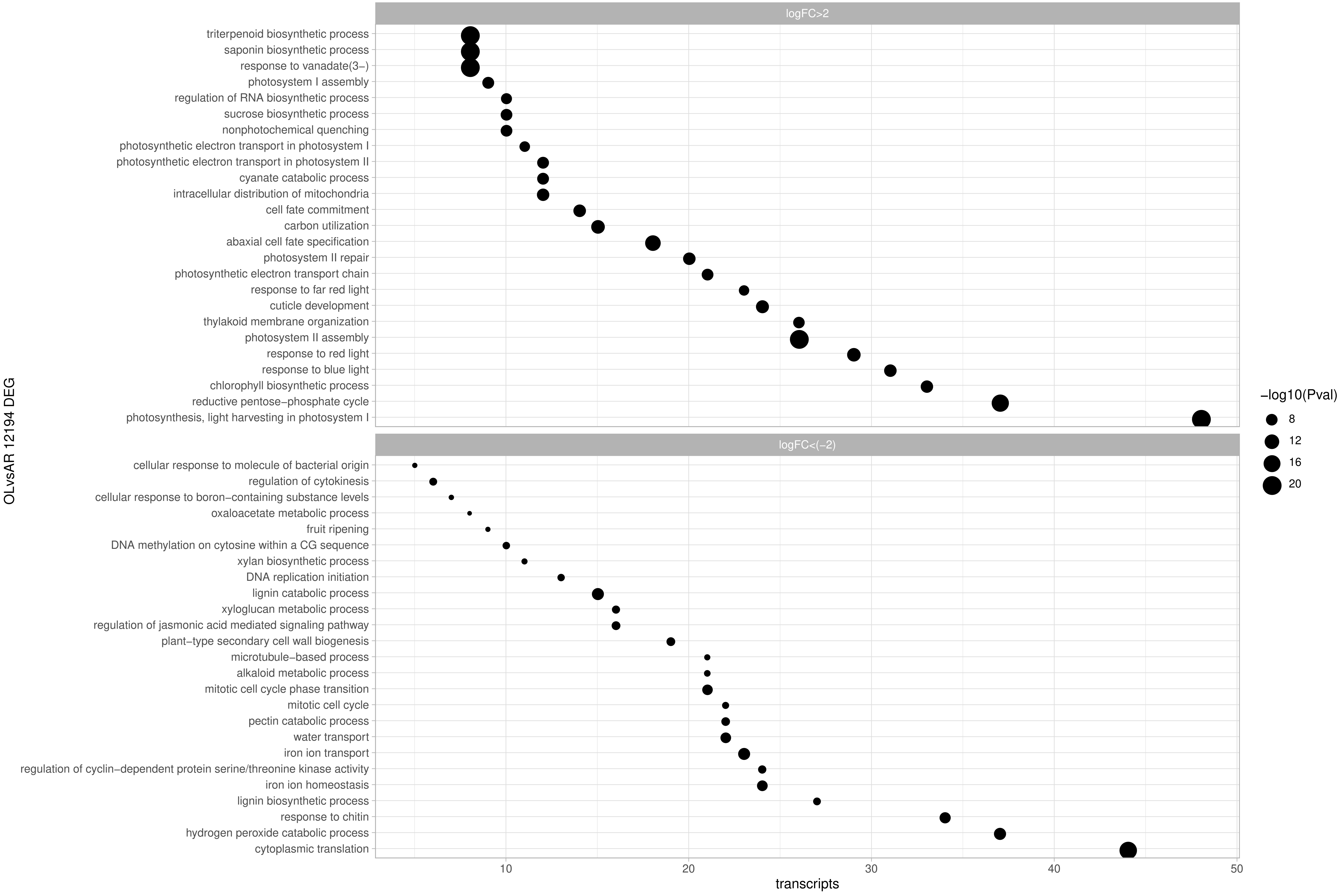

Figure S6. Significantly enriched GO biological pathways (p<0.05) in the upregulated (log fold change > 2) and downregulated (log fold change <2) OLvsAR DGE comparison.

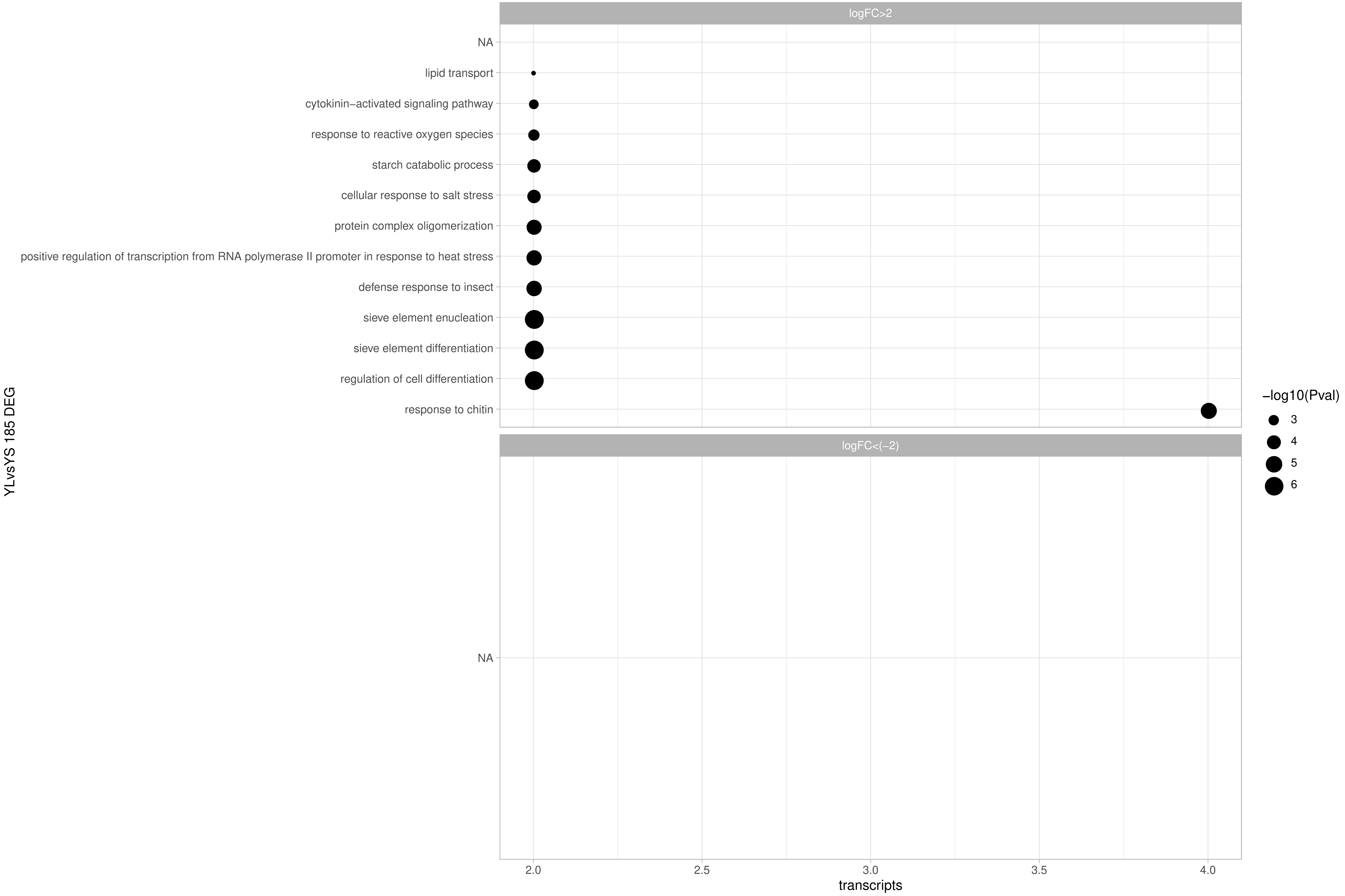

Figure S7. Significantly enriched GO biological pathways (p<0.05) in the upregulated (log fold change > 2) and downregulated (log fold change <2) YLvsYS DEG comparison.

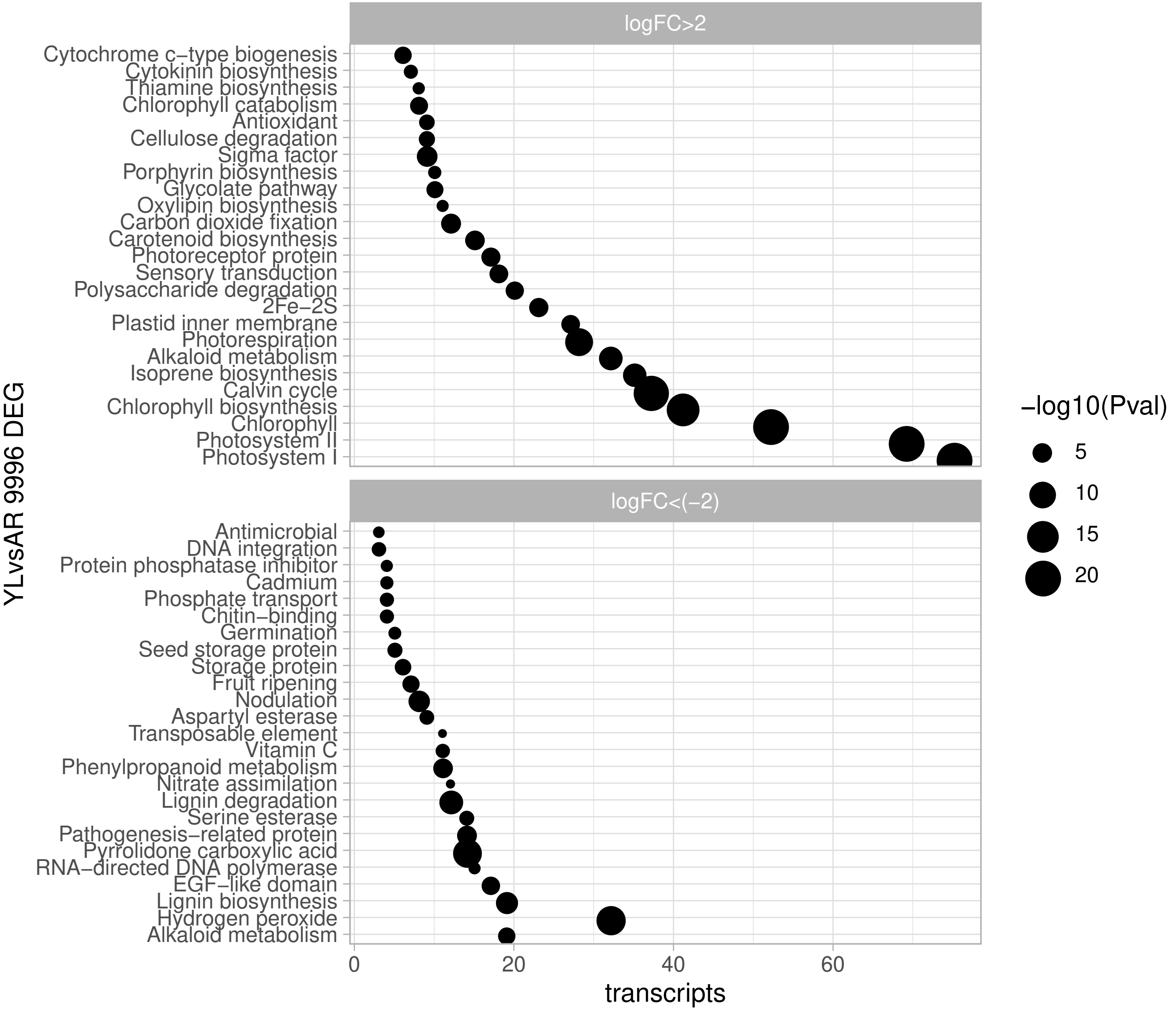

Figure S8. Significantly enriched GO biological pathways ($p<0.05$) in the upregulated (log fold change > 2) and downregulated (log fold change <2) YLvsAR

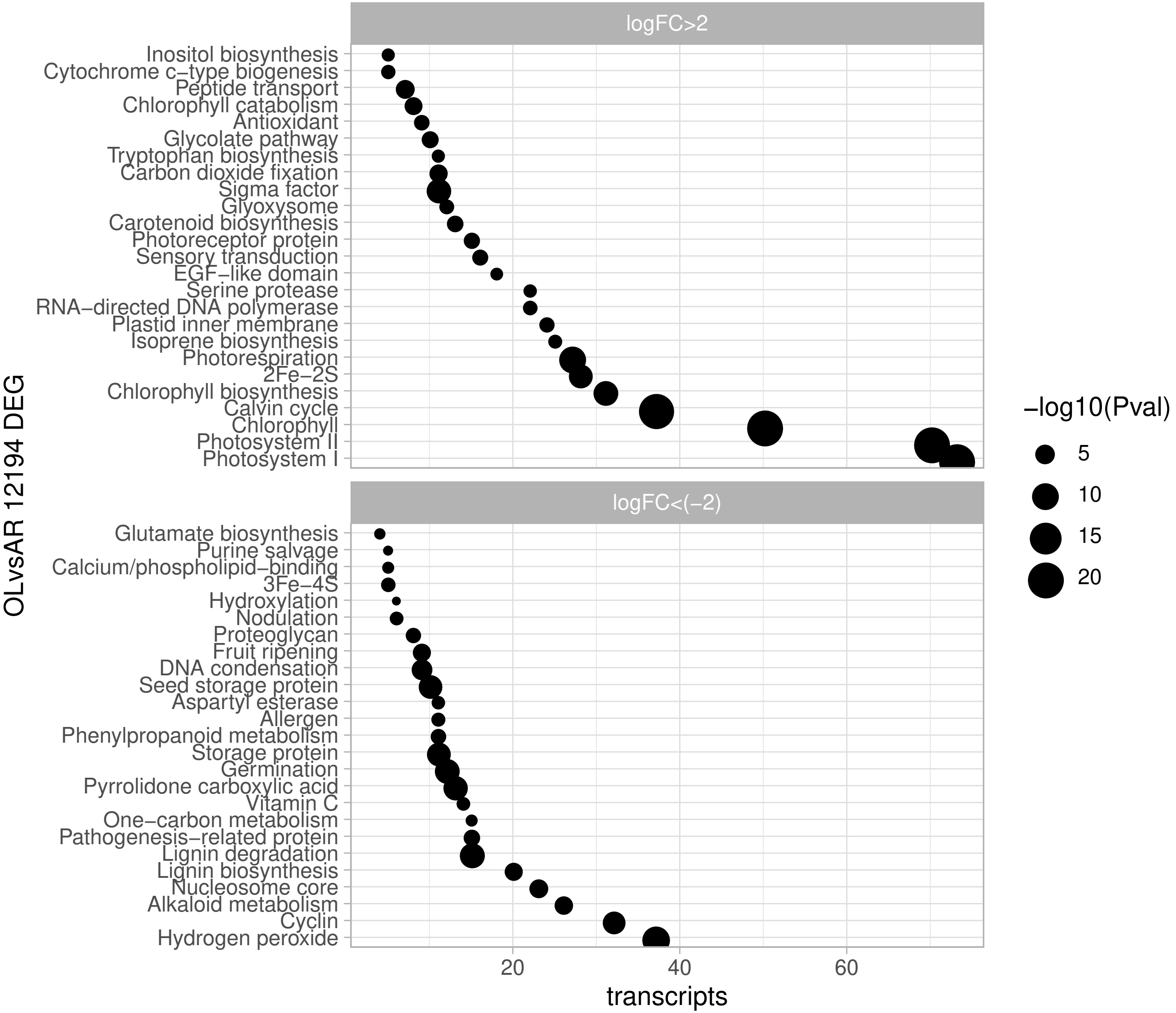

Figure S9. Significantly enriched GO Keywords (p<0.05) in the upregulated (log fold change > 2) and downregulated (log fold change >2) OLvsAR DEG comparison.

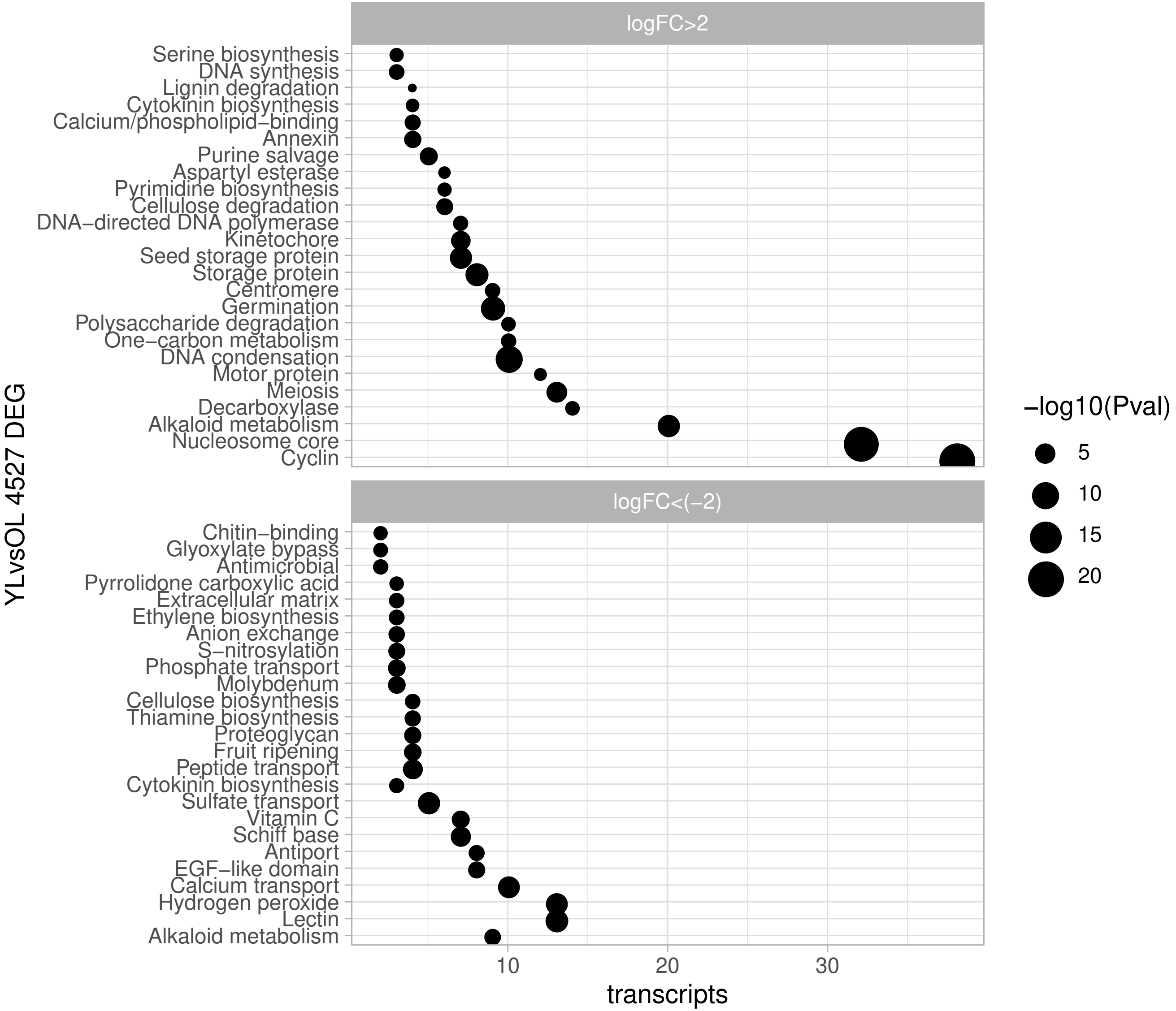

Figure S10. Significantly enriched GO Keywords (p<0.05) in the upregulated (log fold change > 2) and downregulated (log fold change <2) YLvsOL DEG comparison.

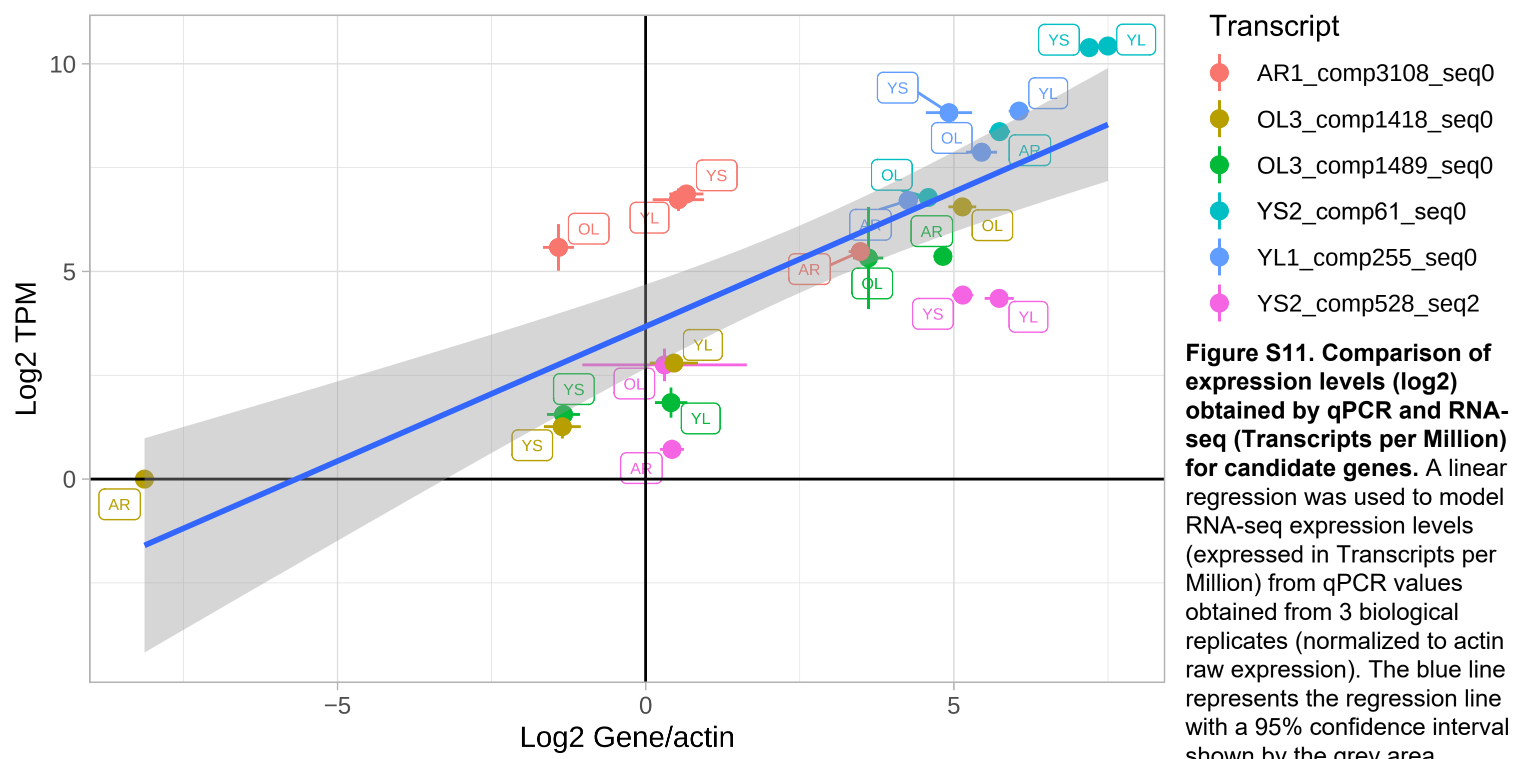

Figure S11. Comparison of expression levels (log2) obtained by qPCR and RNA-seq (Transcripts per Million) for candidate genes. A linear regression was used to model RNA-seq expression levels (expressed in Transcripts per Million) from qPCR values obtained from 3 biological replicates (normalized to actin raw expression). The blue line represents the regression line with a 95% confidence interval shown by the grey area.

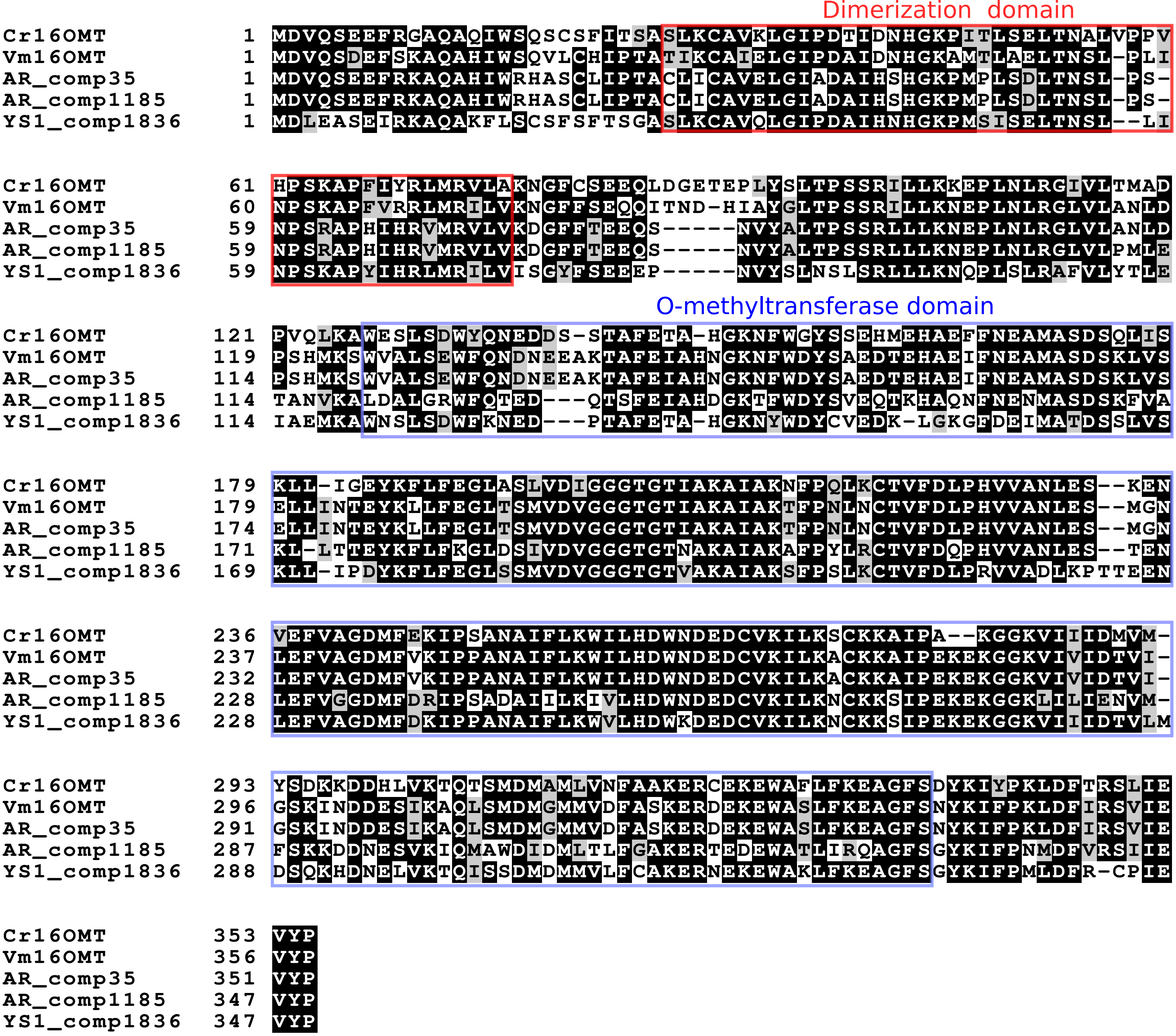

Figure S12. Alignment of the amino-acid sequences of 16-O-tabersonine methyltransferase candidates. Sequence identity and similarity are highlighted by black and grey shading, respectively. The dimerization domain is highlighted in red and the O-methyltransferase domain is highlighted in blue.

A

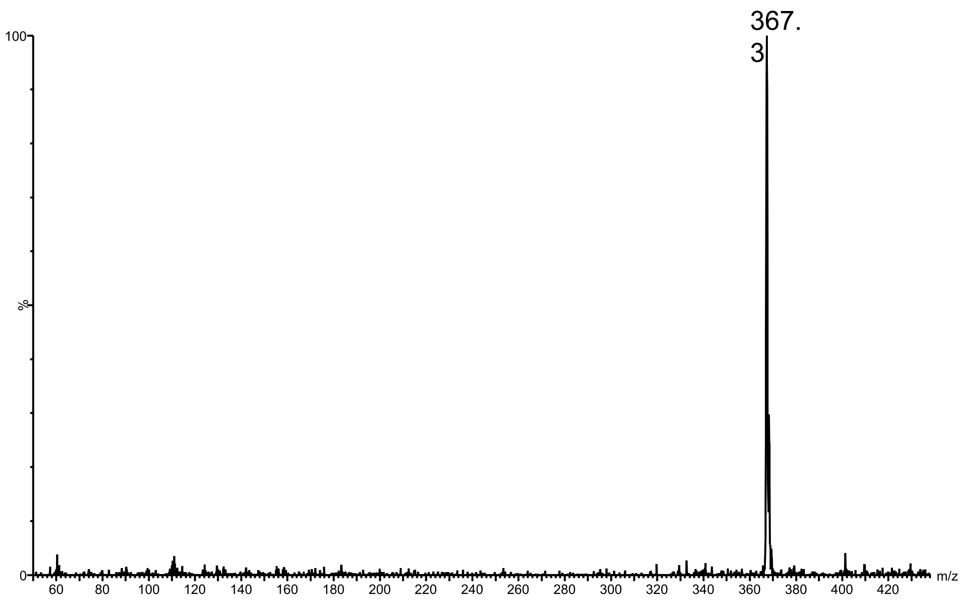

B

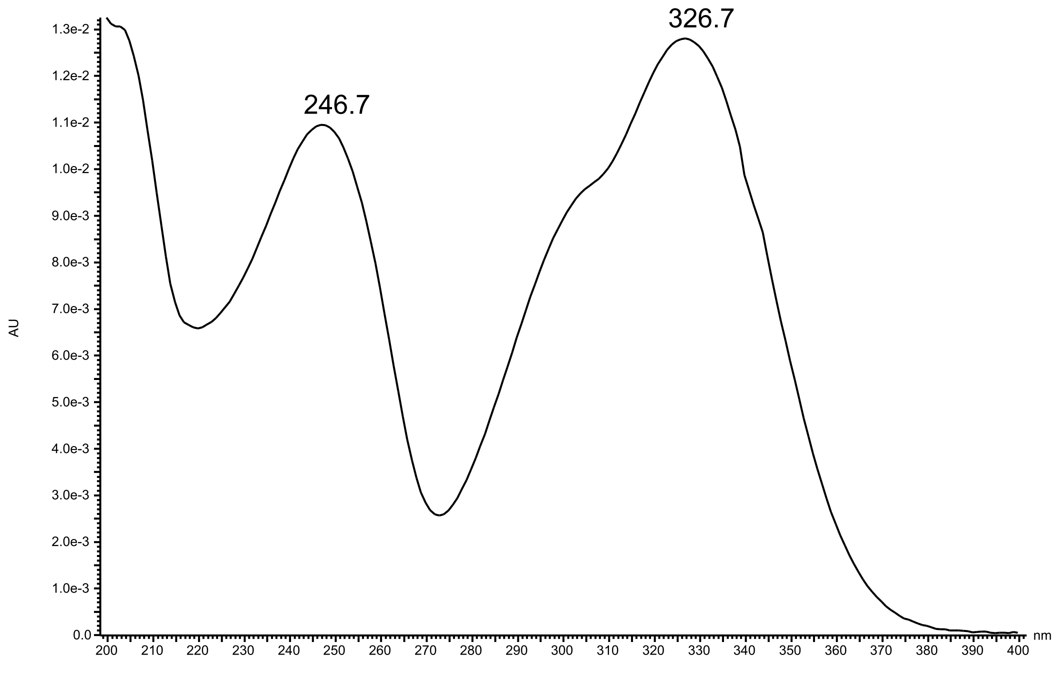

Figure S13. UPLC-DAD-MS analysis of 16-methoxytabersonine. (A) MS spectrum and (B) UV spectrum

TableS1

MIA	Transcript	% ID	Alignment length	Mismatch	Gapopen	MIA start	MIA end	Transcript start	Transcript end	E-value	Bitscore
	YS2_GAGATTCC-ATAGAGGC_comp2127_seq1	85.856	2326	280	9	132	2453	273	2553	0	2715
	YL3_CGCTCATT-ATAGAGGC_comp1779_seq8	86.735	2141	244	7	132	2268	247	2351	0	2585
	YL3_CGCTCATT-ATAGAGGC_comp1779_seq8	81.771	192	26	2	2262	2453	2431	2613	2.94E-46	188
	YL3_CGCTCATT-ATAGAGGC_comp1779_seq5	86.933	1852	199	9	138	1985	398	2210	0	2248
	YL3_CGCTCATT-ATAGAGGC_comp1779_seq5	85.614	285	41	0	1984	2268	2294	2578	1.33E-88	329
	YL3_CGCTCATT-ATAGAGGC_comp1779_seq5	81.771	192	26	2	2262	2453	2658	2840	2.94E-46	188
	AR1_GAGATTCC-CCTATCCT_comp8926_seq1	81.237	1956	367	0	468	2423	1115	3070	0	1873
	OL3_CGCTCATT-GGCTCTGA_comp1793_seq1	85.478	1267	144	7	132	1394	226	1456	0	1460
	OL3_CGCTCATT-GGCTCTGA_comp1793_seq1	87.184	593	74	2	1393	1985	1593	2183	0	720
DXS1 (KC625536.1)	OL3_CGCTCATT-GGCTCTGA_comp1793_seq1	84.12	466	65	2	1984	2449	2267	2723	8.38E-142	506
	YL1_CGCTCATT-TATAGCCT_comp1491_seq4	73.777	1922	496	4	444	2358	295	2215	0	1184
	AR1_GAGATTCC-CCTATCCT_comp8926_seq1	72.282	1959	536	6	444	2397	1099	3055	0	1065
	YS2_GAGATTCC-ATAGAGGC_comp2127_seq1	71.252	1941	541	14	467	2397	584	2517	0	937
	YS1_GAGATTCC-TATAGCCT_comp16391_seq0	74.211	1489	371	7	878	2358	1717	3200	0	935
	YL1_CGCTCATT-TATAGCCT_comp3571_seq0	84.672	822	126	0	871	1692	1	822	0	915
	YL1_CGCTCATT-TATAGCCT_comp3571_seq0	87.847	288	35	0	1970	2257	2335	2622	2.21E-98	361
	YL1_CGCTCATT-TATAGCCT_comp3571_seq0	87.943	282	34	0	1684	1965	1564	1845	9.42E-97	356
DXS2 (DQ848672.1)	YL1_CGCTCATT-TATAGCCT_comp3571_seq0	86.792	159	21	0	2256	2414	3195	3353	2.37E-47	192
	YL1_CGCTCATT-TATAGCCT_comp1491_seq4	87.967	2169	243	5	32	2185	94	2259	0	2729
	YS1_GAGATTCC-TATAGCCT_comp16391_seq0	88.066	1525	182	0	661	2185	1720	3244	0	1930
	AR1_GAGATTCC-CCTATCCT_comp8926_seq1	77.923	1993	430	4	127	2114	1002	2989	0	1602
	YS2_GAGATTCC-ATAGAGGC_comp2127_seq1	70.629	1859	540	4	259	2114	596	2451	0	879
DXS3 (AJ011840.2)	YS2_GAGATTCC-ATAGAGGC_comp28794_seq0	85.752	751	92	4	34	772	50	797	0	868
	YS1_GAGATTCC-TATAGCCT_comp445_seq2	88.005	1634	157	14	1	1613	352	1967	0	2037
	YL3_CGCTCATT-ATAGAGGC_comp492_seq0	87.636	1561	155	14	96	1629	728	2277	0	1918
	YL3_CGCTCATT-ATAGAGGC_comp492_seq0	90.816	98	9	0	1	98	300	397	1.00E-30	136
	YL1_CGCTCATT-TATAGCCT_comp16369_seq2	87.627	1382	133	14	275	1629	849	2219	0	1694
	YL3_CGCTCATT-ATAGAGGC_comp434_seq2	92.004	1113	86	1	1	1110	213	1325	0	1606
	YL3_CGCTCATT-ATAGAGGC_comp434_seq2	79.89	547	72	14	1110	1629	1472	2007	5.51E-129	462
	YS1_GAGATTCC-TATAGCCT_comp445_seq3	91.982	1110	89	0	1	1110	352	1461	0	1602
	YS1_GAGATTCC-TATAGCCT_comp445_seq3	75.75	400	59	14	1257	1629	1892	2280	1.48E-66	255
DXR (AF250235.1)	YS1_GAGATTCC-TATAGCCT_comp445_seq3	91.156	147	13	0	1110	1256	1620	1766	6.74E-52	206
	YS2_GAGATTCC-ATAGAGGC_comp2915_seq1	84.587	1077	120	13	38	1075	1351	2420	0	1177
	YL1_CGCTCATT-TATAGCCT_comp25159_seq0	74.118	85	10	2	949	1024	1	82	6.35E-07	57.2
	YS2_GAGATTCC-ATAGAGGC_comp22987_seq0	95.652	23	1	0	384	406	1	23	0.6	37.4
	OL1_CGCTCATT-CCTATCCT_comp6333_seq2	89.286	28	3	0	662	689	477	450	0.6	37.4
CMS (FJ177510.1)	AR2_GAGATTCC-GGCTCTGA_comp22244_seq0	92.593	27	0	1	1336	1362	25	1	0.6	37.4
	YS1_GAGATTCC-TATAGCCT_comp1496_seq2	84.572	1426	186	12	90	1504	245	1647	0	1557
	AR1_GAGATTCC-CCTATCCT_comp2159_seq1	84.561	1425	187	12	90	1504	281	1682	0	1553
	YL3_CGCTCATT-ATAGAGGC_comp1768_seq0	88.545	969	111	0	90	1058	234	1202	0	1247
	YL3_CGCTCATT-ATAGAGGC_comp1768_seq0	88.136	59	7	0	1227	1285	1719	1777	2.99E-12	75.2

TableS1

	YL3_CGCTCATT-ATAGAGGC_comp1768_seq0	85.417	48	7	0	1169	1216	1199	1246	2.80E-06	55.4
	AR1_GAGATTCC-CCTATCCT_comp2159_seq2	92.323	495	38	0	638	1132	941	1435	0	722
	AR1_GAGATTCC-CCTATCCT_comp2159_seq2	85.278	557	82	0	90	646	281	837	0	636
	AR1_GAGATTCC-CCTATCCT_comp2159_seq2	73.438	384	69	12	1131	1504	1675	2035	2.01E-52	208
CMK (DQ848671.1)	YL3_CGCTCATT-ATAGAGGC_comp18_seq9	76.907	485	78	12	1031	1504	1869	2330	3.18E-94	347
	YS1_GAGATTCC-TATAGCCT_comp2048_seq1	85.405	740	78	7	1	719	179	909	0	843
	AR2_GAGATTCC-GGCTCTGA_comp29267_seq0	92.722	371	27	0	346	716	1002	1372	9.27E-155	547
	YL3_CGCTCATT-ATAGAGGC_comp2559_seq2	82.318	509	60	7	1	488	87	586	8.13E-143	508
	YL3_CGCTCATT-ATAGAGGC_comp2559_seq2	90.431	209	20	0	478	686	1498	1706	1.38E-76	288
	AR2_GAGATTCC-GGCTCTGA_comp5554_seq2	85.676	377	42	3	124	488	197	573	1.47E-120	434
	AR2_GAGATTCC-GGCTCTGA_comp5554_seq2	92.975	242	17	0	478	719	1539	1780	2.66E-98	360
	AR2_GAGATTCC-GGCTCTGA_comp5554_seq2	87.805	41	5	0	1	41	89	129	1.80E-05	51.8
MECS (AF250236.1)	AR1_GAGATTCC-CCTATCCT_comp4429_seq6	81.579	38	7	0	845	882	202	165	0.4	37.4
	YS2_GAGATTCC-ATAGAGGC_comp287_seq1	89.876	2331	220	6	42	2364	139	2461	0	3128
	AR1_GAGATTCC-CCTATCCT_comp972_seq1	89.79	2331	222	6	42	2364	135	2457	0	3119
	AR1_GAGATTCC-CCTATCCT_comp972_seq2	89.79	2331	222	6	42	2364	135	2457	0	3119
	AR1_GAGATTCC-CCTATCCT_comp972_seq6	89.79	2331	222	6	42	2364	135	2457	0	3119
	OL1_CGCTCATT-CCTATCCT_comp301_seq2	90.707	2249	209	0	116	2364	398	2646	0	3113
HDS (AY184810.2)	OL1_CGCTCATT-CCTATCCT_comp301_seq2	92.308	26	2	0	52	77	206	231	0.3	39.2
	YS1_GAGATTCC-TATAGCCT_comp439_seq2	88.865	1419	122	3	1	1392	147	1556	0	1865
	AR2_GAGATTCC-GGCTCTGA_comp1917_seq2	89.177	1349	113	2	1	1325	88	1427	0	1795
	AR2_GAGATTCC-GGCTCTGA_comp1917_seq2	86.486	74	7	1	1322	1392	1580	1653	3.82E-16	87.8
	YS1_GAGATTCC-TATAGCCT_comp439_seq0	92.056	1133	87	1	263	1392	882	2014	0	1636
	YS1_GAGATTCC-TATAGCCT_comp439_seq0	79.508	244	33	5	40	267	3	245	1.62E-52	208
	OL3_CGCTCATT-GGCTCTGA_comp26094_seq0	91.56	936	76	1	460	1392	934	1869	0	1330
	OL3_CGCTCATT-GGCTCTGA_comp26094_seq0	93.939	198	12	0	265	462	3	200	2.74E-81	304
	YL3_CGCTCATT-ATAGAGGC_comp21618_seq1	91.799	756	59	1	640	1392	410	1165	0	1083
HDR (DQ848676.1)	YL3_CGCTCATT-ATAGAGGC_comp21618_seq1	93.145	248	17	0	397	644	1	248	2.25E-101	370
	AR1_GAGATTCC-CCTATCCT_comp1158_seq0	81.466	1187	145	25	36	1206	120	1247	0	1104
	YS2_GAGATTCC-ATAGAGGC_comp566_seq2	84.258	775	89	10	388	1156	1384	2131	0	832
	YS2_GAGATTCC-ATAGAGGC_comp566_seq2	77.344	256	28	7	137	389	19	247	9.33E-49	196
	AR2_GAGATTCC-GGCTCTGA_comp16125_seq3	83.484	775	95	10	388	1156	3012	3759	0	805
	AR2_GAGATTCC-GGCTCTGA_comp16125_seq3	79.31	58	1	1	332	389	1829	1875	5.90E-07	57.2
	YS2_GAGATTCC-ATAGAGGC_comp29331_seq0	92.727	165	12	0	315	479	1	165	2.05E-63	244
	YS2_GAGATTCC-ATAGAGGC_comp29331_seq0	92.391	92	7	0	477	568	1003	1094	2.68E-30	134
IDI1 (EU135981.1)	YS1_GAGATTCC-TATAGCCT_comp15918_seq0	96.552	58	2	0	332	389	1203	1260	6.72E-19	96.9
	AR1_GAGATTCC-CCTATCCT_comp8039_seq0	87.905	1265	147	2	2	1263	301	1562	0	1588
	AR1_GAGATTCC-CCTATCCT_comp30038_seq0	89.655	29	3	0	1196	1224	30	2	0.16	39.2
	YL3_CGCTCATT-ATAGAGGC_comp1244_seq0	95.455	22	1	0	39	60	3610	3589	1.9	35.6
	AR2_GAGATTCC-GGCTCTGA_comp5097_seq2	91.667	24	2	0	691	714	2295	2318	1.9	35.6
GPPS (KF561462.1)	YL3_CGCTCATT-ATAGAGGC_comp1244_seq6	95.455	22	1	0	39	60	3498	3477	1.9	35.6

	YL1_CGCTCATT-TATAGCCT_comp695_seq0	85.882	1615	215	6	153	1762	323	1929	0	1869
	YL1_CGCTCATT-TATAGCCT_comp695_seq0	89.189	37	4	0	91	127	285	321	1.23E-04	50
	YS1_GAGATTCC-TATAGCCT_comp841_seq0	87.888	677	77	2	1090	1762	1649	2324	0	847
	OL3_CGCTCATT-GGCTCTGA_comp6736_seq2	80.46	783	121	5	93	874	185	936	0	729
	YL3_CGCTCATT-ATAGAGGC_comp13409_seq0	86.965	537	65	2	1230	1762	874	1409	0	648
	YL3_CGCTCATT-ATAGAGGC_comp13409_seq0	95.833	24	1	0	1090	1113	390	413	0.22	39.2
	YS1_GAGATTCC-TATAGCCT_comp19336_seq1	87.847	288	35	0	943	1230	2	289	1.49E-98	361
	YS1_GAGATTCC-TATAGCCT_comp19336_seq1	88.764	89	10	0	1481	1569	1169	1257	1.01E-24	116
GES (JN882024.1)	YS1_GAGATTCC-TATAGCCT_comp19336_seq1	95.833	24	1	0	1230	1253	726	749	0.22	39.2
	AR1_GAGATTCC-CCTATCCT_comp589_seq7	79.961	1537	300	2	23	1551	1306	2842	0	1382
	AR1_GAGATTCC-CCTATCCT_comp2452_seq0	81.882	861	156	0	316	1176	1	861	0	850
	AR1_GAGATTCC-CCTATCCT_comp2452_seq0	79.191	173	26	2	1385	1555	866	1030	4.58E-35	150
	YL3_CGCTCATT-ATAGAGGC_comp1775_seq0	80.248	805	149	2	753	1555	2	798	0	735
	YS1_GAGATTCC-TATAGCCT_comp8154_seq1	80.863	371	61	2	1187	1555	5	367	8.82E-95	349
G10H (AJ251269.1)	AR1_GAGATTCC-CCTATCCT_comp7341_seq0	73.913	184	48	0	1164	1347	1434	1617	9.45E-25	116
	YL3_CGCTCATT-ATAGAGGC_comp70_seq0	87.871	1113	135	0	129	1241	146	1258	0	1398
	OL3_CGCTCATT-GGCTCTGA_comp191_seq2	86.667	600	80	0	642	1241	1622	2221	0	722
	AR1_GAGATTCC-CCTATCCT_comp635_seq0	68.704	949	280	9	298	1233	2061	3005	2.28E-94	347
	AR1_GAGATTCC-CCTATCCT_comp635_seq0	72.185	151	36	2	136	283	1792	1939	5.04E-14	80.6
	YS1_GAGATTCC-TATAGCCT_comp6458_seq0	82.158	241	43	0	732	972	1	241	2.44E-62	241
10HGO (KF302069.1)	YS2_GAGATTCC-ATAGAGGC_comp2835_seq1	82.553	235	41	0	738	972	1	235	8.52E-62	239
	YL3_CGCTCATT-ATAGAGGC_comp477_seq1	84.9	1053	159	0	115	1167	409	1461	0	1182
	YL3_CGCTCATT-ATAGAGGC_comp477_seq1	86.667	120	16	0	1	120	199	318	1.36E-33	145
	YS1_GAGATTCC-TATAGCCT_comp296_seq0	69.685	1049	292	11	78	1106	302	1344	6.55E-120	432
	OL1_CGCTCATT-CCTATCCT_comp26_seq0	65.448	1094	340	15	82	1153	195	1272	9.75E-61	235
	OL1_CGCTCATT-CCTATCCT_comp11291_seq2	66.289	353	97	9	758	1099	1363	1704	4.74E-14	80.6
	OL1_CGCTCATT-CCTATCCT_comp11291_seq2	95.652	23	0	1	871	893	1552	1573	6.2	33.7
IS (JX974564.1)	AR2_GAGATTCC-GGCTCTGA_comp6919_seq0	84.211	38	5	1	37	74	234	270	0.15	39.2
	OL1_CGCTCATT-CCTATCCT_comp520_seq1	83.735	1537	246	2	14	1548	261	1795	0	1640
	AR2_GAGATTCC-GGCTCTGA_comp3144_seq0	85.755	351	50	0	1198	1548	431	781	9.97E-113	408
	OL3_CGCTCATT-GGCTCTGA_comp13994_seq0	89.416	274	29	0	661	934	3	276	3.72E-99	363
	YS1_GAGATTCC-TATAGCCT_comp11762_seq0	89.286	252	27	0	683	934	1	252	6.30E-90	333
	YS1_GAGATTCC-TATAGCCT_comp11762_seq0	80.682	88	17	0	1206	1293	1686	1773	1.81E-14	82.4
	YS1_GAGATTCC-TATAGCCT_comp17871_seq1	90.244	205	20	0	730	934	1	205	3.49E-74	280
IO (KF302066.1)	YS1_GAGATTCC-TATAGCCT_comp17871_seq1	86.869	99	13	0	933	1031	1040	1138	7.21E-26	120
	YS2_GAGATTCC-ATAGAGGC_comp558_seq3	84.855	1446	219	0	75	1520	832	2277	0	1620
	OL3_CGCTCATT-GGCTCTGA_comp2126_seq0	84.165	1162	184	0	70	1231	72	1233	0	1267
	YS2_GAGATTCC-ATAGAGGC_comp8577_seq0	67.845	1415	415	20	98	1492	162	1556	4.50E-124	446
	YL3_CGCTCATT-ATAGAGGC_comp1412_seq1	83.74	369	60	0	1160	1528	1388	1756	7.13E-109	396
7DLGT (AB733667.1)	YS1_GAGATTCC-TATAGCCT_comp607_seq1	84.928	345	52	0	1184	1528	1063	1407	1.06E-106	389
	YL1_CGCTCATT-TATAGCCT_comp444_seq0	85.597	1222	156	7	662	1874	835	2045	0	1397

TableS1

	YL1_CGCTCATT-TATAGCCT_comp444_seq0	86.364	506	66	1	165	667	236	741	3.58E-170	600
	OL1_CGCTCATT-CCTATCCT_comp2662_seq1	68.421	1121	329	13	614	1726	783	1886	3.36E-107	390
	OL1_CGCTCATT-CCTATCCT_comp2662_seq1	73.469	98	26	0	233	330	408	505	7.44E-08	60.8
	AR2_GAGATTCC-GGCTCTGA_comp35_seq9	66.021	1498	477	14	233	1717	742	2220	9.02E-102	372
	OL3_CGCTCATT-GGCTCTGA_comp1360_seq3	68.489	1006	280	16	735	1726	1112	2094	3.59E-94	347
7DLH (KF415115.1)	AR2_GAGATTCC-GGCTCTGA_comp17345_seq0	67.033	1183	349	16	569	1735	494	1651	5.33E-92	340
	YS2_GAGATTCC-ATAGAGGC_comp88_seq3	85.714	1078	151	1	39	1116	385	1459	0	1249
	YS2_GAGATTCC-ATAGAGGC_comp1328_seq0	86.269	1005	135	1	112	1116	5289	6290	0	1189
	YL1_CGCTCATT-TATAGCCT_comp5423_seq0	84.903	828	122	1	39	866	2873	3697	0	928
	YL1_CGCTCATT-TATAGCCT_comp5423_seq0	89.535	344	36	0	773	1116	2120	2463	4.50E-128	459
	YL1_CGCTCATT-TATAGCCT_comp5423_seq0	83.871	31	5	0	551	581	878	908	5.9	33.7
	OL3_CGCTCATT-GGCTCTGA_comp1728_seq2	83.862	756	116	2	24	779	238	987	0	810
	OL3_CGCTCATT-GGCTCTGA_comp1728_seq2	88.663	344	39	0	773	1116	2169	2512	9.91E-124	444
LAMT (EU057974.1)	YS1_GAGATTCC-TATAGCCT_comp18495_seq0	88.53	558	64	0	559	1116	2842	3399	0	719
	AR2_GAGATTCC-GGCTCTGA_comp35_seq9	86.951	1617	203	2	15	1630	631	2240	0	1963
	YS1_GAGATTCC-TATAGCCT_comp24972_seq0	87.259	1295	165	0	342	1636	1436	2730	0	1591
	YS1_GAGATTCC-TATAGCCT_comp24972_seq0	86.722	241	32	0	105	345	3	243	2.32E-77	291
	YS1_GAGATTCC-TATAGCCT_comp24972_seq0	72.973	74	5	3	1661	1732	2785	2845	0.005	44.6
	YL1_CGCTCATT-TATAGCCT_comp7664_seq0	86.38	1138	155	0	499	1636	317	1454	0	1353
	YL1_CGCTCATT-TATAGCCT_comp7664_seq0	83.929	168	27	0	63	230	150	317	3.02E-44	181
	YL1_CGCTCATT-TATAGCCT_comp7664_seq0	72.973	74	5	3	1661	1732	1509	1569	0.005	44.6
	YL1_CGCTCATT-TATAGCCT_comp14816_seq0	86.883	831	109	0	806	1636	424	1254	0	1007
	YL1_CGCTCATT-TATAGCCT_comp14816_seq0	89.6	250	26	0	565	814	1	250	2.17E-90	334
	YL1_CGCTCATT-TATAGCCT_comp14816_seq0	72.973	74	5	3	1661	1732	1309	1369	0.005	44.6
	YS2_GAGATTCC-ATAGAGGC_comp2723_seq0	86.845	821	108	0	816	1636	478	1298	0	994
	YS2_GAGATTCC-ATAGAGGC_comp2723_seq0	86.264	182	25	0	52	233	101	282	1.46E-54	215
SLS1 (L10081.1)	YS2_GAGATTCC-ATAGAGGC_comp2723_seq0	72.973	74	5	3	1661	1732	1353	1413	0.005	44.6
	AR2_GAGATTCC-GGCTCTGA_comp35_seq9	87.626	1584	196	0	1	1584	667	2250	0	1974
	YS1_GAGATTCC-TATAGCCT_comp24972_seq0	87.792	1286	157	0	286	1571	1436	2721	0	1611
	YS1_GAGATTCC-TATAGCCT_comp24972_seq0	86.307	241	33	0	49	289	3	243	8.40E-76	286
	YL1_CGCTCATT-TATAGCCT_comp7664_seq0	86.714	1129	150	0	443	1571	317	1445	0	1361
	YL1_CGCTCATT-TATAGCCT_comp7664_seq0	82.738	168	29	0	7	174	150	317	1.33E-41	172
	YL1_CGCTCATT-TATAGCCT_comp14816_seq0	87.698	821	101	0	751	1571	425	1245	0	1025
	YL1_CGCTCATT-TATAGCCT_comp14816_seq0	86.8	250	33	0	509	758	1	250	1.09E-80	302
	YS2_GAGATTCC-ATAGAGGC_comp2723_seq0	87.562	812	101	0	760	1571	478	1289	0	1009
	YS2_GAGATTCC-ATAGAGGC_comp2723_seq0	84.746	177	27	0	1	177	106	282	3.35E-49	197
SLS2 (KF309242.1)	YS2_GAGATTCC-ATAGAGGC_comp2723_seq0	90	30	3	0	503	532	454	483	0.057	41
	known MIA genes from C. roseu	78.534	955	195	4	44	991	102	1053	0	789
	YS2_GAGATTCC-ATAGAGGC_comp2161_seq0	82.051	39	7	0	815	853	8327	8365	0.15	39.2
	YL3_CGCTCATT-ATAGAGGC_comp4991_seq1	82.051	39	7	0	815	853	2791	2829	0.15	39.2
	OL1_CGCTCATT-CCTATCCT_comp1693_seq1	92.308	26	2	0	904	929	1163	1188	0.15	39.2

TableS1

STR (X53602.1)	YL3_CGCTCATT-ATAGAGGC_comp10885_seq1	95.652	23	1	0	1045	1067	1410	1388	0.51	37.4
	YS2_GAGATTCC-ATAGAGGC_comp324_seq0	80.227	1057	201	4	1	1050	661	1716	0	953
	YS2_GAGATTCC-ATAGAGGC_comp324_seq0	75.844	385	88	2	1050	1433	1786	2166	1.67E-71	271
	YL3_CGCTCATT-ATAGAGGC_comp4131_seq0	70.44	159	45	2	824	981	1727	1884	3.69E-10	68
	YS1_GAGATTCC-TATAGCCT_comp9626_seq1	84.211	38	5	1	831	868	668	632	0.18	39.2
	AR1_GAGATTCC-CCTATCCT_comp8861_seq1	100	21	0	0	437	457	1687	1667	0.18	39.2
TDC (MG748691.1)	YL3_CGCTCATT-ATAGAGGC_comp9172_seq1	84.211	38	5	1	831	868	659	623	0.18	39.2
	YL1_CGCTCATT-TATAGCCT_comp255_seq0	79.31	1537	265	11	133	1662	1595	3085	0	1337
	OL1_CGCTCATT-CCTATCCT_comp1264_seq0	77.739	867	184	5	417	1277	391	1254	0	679
	OL1_CGCTCATT-CCTATCCT_comp1264_seq0	85.256	156	23	0	1430	1585	1251	1406	3.30E-43	178
	OL1_CGCTCATT-CCTATCCT_comp1264_seq0	86.429	140	19	0	263	402	76	215	5.97E-40	167
	OL3_CGCTCATT-GGCTCTGA_comp686_seq1	83.443	610	98	2	478	1084	958	1567	0	639
	OL3_CGCTCATT-GGCTCTGA_comp686_seq1	87.931	348	40	2	133	479	517	863	9.43E-120	432
	OL3_CGCTCATT-GGCTCTGA_comp686_seq1	73.469	490	100	3	1173	1662	1838	2297	2.22E-83	311
	YL3_CGCTCATT-ATAGAGGC_comp1113_seq0	71.298	655	152	8	931	1580	2	625	2.37E-89	331
	AR2_GAGATTCC-GGCTCTGA_comp5833_seq1	67.018	949	282	8	142	1082	378	1303	5.96E-78	293
SGD (EU072423.1)	AR2_GAGATTCC-GGCTCTGA_comp5833_seq1	67.974	153	46	1	1417	1566	1608	1760	9.48E-06	53.6
	OL1_CGCTCATT-CCTATCCT_comp374_seq0	86.575	1095	147	0	1	1095	176	1270	0	1312
	AR2_GAGATTCC-GGCTCTGA_comp706_seq1	86.066	1098	150	1	1	1095	61	1158	0	1288
	AR2_GAGATTCC-GGCTCTGA_comp706_seq3	86.066	1098	150	1	1	1095	61	1158	0	1288
	AR2_GAGATTCC-GGCTCTGA_comp706_seq4	86.066	1098	150	1	1	1095	61	1158	0	1288
GS1 (KF302079.1)	YL3_CGCTCATT-ATAGAGGC_comp123_seq0	85.845	1095	155	0	1	1095	2080	3174	0	1276
	OL1_CGCTCATT-CCTATCCT_comp374_seq0	84.081	1093	168	1	1	1087	176	1268	0	1187
	YL3_CGCTCATT-ATAGAGGC_comp123_seq0	83.715	1093	172	1	1	1087	2080	3172	0	1169
	AR2_GAGATTCC-GGCTCTGA_comp706_seq1	83.212	1096	175	2	1	1087	61	1156	0	1146
	AR2_GAGATTCC-GGCTCTGA_comp706_seq3	83.212	1096	175	2	1	1087	61	1156	0	1146
GS2 (KF302078.1)	AR2_GAGATTCC-GGCTCTGA_comp706_seq4	83.212	1096	175	2	1	1087	61	1156	0	1146
	AR1_GAGATTCC-CCTATCCT_comp3108_seq0	81.699	1071	181	2	6	1064	157	1224	0	1052
	OL1_CGCTCATT-CCTATCCT_comp14114_seq0	74.637	757	160	7	325	1062	501	1244	1.72E-139	497
	YL3_CGCTCATT-ATAGAGGC_comp42_seq0	69.436	1047	276	11	40	1052	177	1213	4.92E-121	435
	AR1_GAGATTCC-CCTATCCT_comp80_seq0	67.739	1057	305	10	38	1064	287	1337	3.79E-97	356
THAS1 (KM524258.1)	AR1_GAGATTCC-CCTATCCT_comp3050_seq0	67.739	1057	305	10	38	1064	281	1331	3.79E-97	356
	OL1_CGCTCATT-CCTATCCT_comp3640_seq0	75.909	1100	260	3	18	1116	210	1305	0	780
	YS2_GAGATTCC-ATAGAGGC_comp697_seq4	78.672	783	162	3	11	792	98	876	0	650
	YS2_GAGATTCC-ATAGAGGC_comp697_seq4	72.566	113	31	0	949	1061	1033	1145	3.48E-09	64.4
	OL3_CGCTCATT-GGCTCTGA_comp1793_seq1	73.4	1094	284	5	25	1116	2866	3954	0	645
	YS2_GAGATTCC-ATAGAGGC_comp4098_seq3	73.4	1094	284	5	25	1116	4818	5906	0	645
THAS2 (KU865323.1)	YL1_CGCTCATT-TATAGCCT_comp13612_seq0	75.938	906	213	3	206	1110	847	1748	0	643
	OL1_CGCTCATT-CCTATCCT_comp3640_seq0	81.088	1121	192	6	18	1134	201	1305	0	1058
	OL3_CGCTCATT-GGCTCTGA_comp1793_seq1	80.454	926	161	6	213	1134	3045	3954	0	845
	YS2_GAGATTCC-ATAGAGGC_comp4098_seq3	80.454	926	161	6	213	1134	4997	5906	0	845

TableS1

	YL1_CGCTCATT-TATAGCCT_comp13612_seq0	79.563	915	173	4	215	1128	847	1748	0	801
THAS3 (KU865322.1)	YS2_GAGATTCC-ATAGAGGC_comp697_seq4	70.31	1098	302	9	18	1109	96	1175	7.74E-138	491
	AR1_GAGATTCC-CCTATCCT_comp3108_seq0	81.648	1068	194	2	1	1067	158	1224	0	1036
	OL1_CGCTCATT-CCTATCCT_comp14114_seq0	76.923	767	159	7	305	1065	490	1244	9.57E-162	571
	AR1_GAGATTCC-CCTATCCT_comp80_seq0	68.587	1076	320	9	7	1067	265	1337	5.31E-108	392
	OL1_CGCTCATT-CCTATCCT_comp1134_seq1	68.03	1076	326	9	7	1067	141	1213	7.38E-100	365
THAS4 (KU865324.1)	AR1_GAGATTCC-CCTATCCT_comp3050_seq0	67.844	1076	328	9	7	1067	259	1331	3.82E-97	356
	AR1_GAGATTCC-CCTATCCT_comp3108_seq0	82.303	1068	187	2	1	1067	158	1224	0	1067
	OL1_CGCTCATT-CCTATCCT_comp14114_seq0	77.004	761	159	6	310	1065	495	1244	9.57E-162	571
	AR1_GAGATTCC-CCTATCCT_comp80_seq0	69.274	1074	316	6	7	1067	265	1337	4.96E-121	435
	OL1_CGCTCATT-CCTATCCT_comp1134_seq1	68.715	1074	322	6	7	1067	141	1213	6.90E-113	408
HYS (KU865325.1)	AR1_GAGATTCC-CCTATCCT_comp3050_seq0	68.063	1074	329	6	7	1067	259	1331	4.08E-103	376
	YL1_CGCTCATT-TATAGCCT_comp32_seq1	82.626	1508	256	2	1	1505	244	1748	0	1535
	YL1_CGCTCATT-TATAGCCT_comp32_seq4	82.626	1508	256	2	1	1505	244	1748	0	1535
	YL3_CGCTCATT-ATAGAGGC_comp58_seq1	82.56	1508	257	2	1	1505	201	1705	0	1530
	YS2_GAGATTCC-ATAGAGGC_comp3760_seq0	84.491	619	96	0	887	1505	992	1610	0	684
GO (MF770508.1)	AR2_GAGATTCC-GGCTCTGA_comp139_seq0	67.253	1478	432	21	7	1461	173	1621	6.98E-121	435
	OL3_CGCTCATT-GGCTCTGA_comp6787_seq2	85.747	863	123	0	1	863	37	899	0	1002
	YL1_CGCTCATT-TATAGCCT_comp16622_seq1	85.315	858	126	0	194	1051	1429	2286	0	980
	YS2_GAGATTCC-ATAGAGGC_comp697_seq1	85.315	858	126	0	194	1051	1531	2388	0	980
	YL3_CGCTCATT-ATAGAGGC_comp42_seq0	81.526	996	184	0	56	1051	223	1218	0	967
redox1 (MF770509.1)	AR1_GAGATTCC-CCTATCCT_comp530_seq0	77.333	975	210	4	84	1051	371	1341	0	755
	YL3_CGCTCATT-ATAGAGGC_comp101_seq3	82.613	972	169	0	1	972	133	1104	0	991
	AR2_GAGATTCC-GGCTCTGA_comp687_seq0	80.844	569	109	0	1	569	98	666	6.19E-151	535
	AR2_GAGATTCC-GGCTCTGA_comp687_seq0	85.217	230	34	0	743	972	1832	2061	5.82E-69	262
	AR2_GAGATTCC-GGCTCTGA_comp687_seq0	87.634	186	23	0	564	749	1009	1194	9.85E-60	232
	YL3_CGCTCATT-ATAGAGGC_comp101_seq2	80.316	569	112	0	1	569	133	701	3.91E-147	522
	YL3_CGCTCATT-ATAGAGGC_comp101_seq2	86.308	409	56	0	564	972	1044	1452	2.81E-136	486
	YS1_GAGATTCC-TATAGCCT_comp65_seq1	80.316	569	112	0	1	569	94	662	3.91E-147	522
	YS1_GAGATTCC-TATAGCCT_comp65_seq1	86.308	409	56	0	564	972	1005	1413	2.81E-136	486
	AR2_GAGATTCC-GGCTCTGA_comp12013_seq0	73.893	858	210	7	37	887	199	1049	4.76E-146	518
redox2 (MF770510.1)	AR2_GAGATTCC-GGCTCTGA_comp12013_seq0	91.667	24	2	0	949	972	1078	1101	1.5	35.6
	YL3_CGCTCATT-ATAGAGGC_comp1258_seq0	79.013	1277	242	7	1	1261	163	1429	0	1086
	AR2_GAGATTCC-GGCTCTGA_comp10632_seq0	71.654	254	61	5	122	368	178	427	1.68E-26	122
	AR2_GAGATTCC-GGCTCTGA_comp10632_seq0	70.588	119	33	2	740	857	835	952	8.72E-05	50
	YL1_CGCTCATT-TATAGCCT_comp10318_seq0	63.83	799	238	18	11	782	67	841	4.51E-21	104
	YL1_CGCTCATT-TATAGCCT_comp10318_seq0	71.895	153	43	0	1089	1241	1184	1336	1.48E-14	82.4
	YS2_GAGATTCC-ATAGAGGC_comp4073_seq1	81.034	58	11	0	1048	1105	1174	1231	2.05E-06	55.4
SAT (MF770511.1)	YS1_GAGATTCC-TATAGCCT_comp9325_seq0	93.75	32	2	0	1073	1104	1188	1219	8.72E-05	50
	YS2_GAGATTCC-ATAGAGGC_comp776_seq0	76.106	1582	344	7	16	1579	126	1691	0	1148
	AR2_GAGATTCC-GGCTCTGA_comp14833_seq0	67.846	1583	453	21	11	1568	4	1555	2.75E-145	517

TableS1

	AR2_GAGATTCC-GGCTCTGA_comp21526_seq0	71.014	414	120	0	397	810	539	952	6.49E-52	206
	AR2_GAGATTCC-GGCTCTGA_comp21526_seq0	71.823	181	45	3	143	317	217	397	1.25E-16	89.7
	AR2_GAGATTCC-GGCTCTGA_comp21526_seq0	72.794	136	36	1	1445	1579	1569	1704	2.76E-12	75.2
	AR1_GAGATTCC-CCTATCCT_comp1722_seq0	70.36	361	107	0	432	792	500	860	1.63E-40	168
PAS	YL1_CGCTCATT-TATAGCCT_comp1884_seq0	67.778	450	145	0	252	701	597	1046	2.95E-37	158
	YL1_CGCTCATT-TATAGCCT_comp695_seq0	85.882	1615	215	6	153	1762	323	1929	0	1869
	YL1_CGCTCATT-TATAGCCT_comp695_seq0	89.189	37	4	0	91	127	285	321	0.000123	50
	YS1_GAGATTCC-TATAGCCT_comp841_seq0	87.888	677	77	2	1090	1762	1649	2324	0	847
	OL3_CGCTCATT-GGCTCTGA_comp6736_seq2	80.46	783	121	5	93	874	185	936	0	729
	YL3_CGCTCATT-ATAGAGGC_comp13409_seq0	86.965	537	65	2	1230	1762	874	1409	0	648
	YL3_CGCTCATT-ATAGAGGC_comp13409_seq0	95.833	24	1	0	1090	1113	390	413	0.22	39.2
	YS1_GAGATTCC-TATAGCCT_comp19336_seq1	87.847	288	35	0	943	1230	2	289	1.49E-98	361
	YS1_GAGATTCC-TATAGCCT_comp19336_seq1	88.764	89	10	0	1481	1569	1169	1257	1.01E-24	116
DPAS (KU865331)	YS1_GAGATTCC-TATAGCCT_comp19336_seq1	95.833	24	1	0	1230	1253	726	749	0.22	39.2
	YL3_CGCTCATT-ATAGAGGC_comp166_seq2	74.669	983	216	9	10	983	284	1242	0	639
	AR1_GAGATTCC-CCTATCCT_comp606_seq1	73.333	510	116	4	475	983	381	871	1.59E-82	307
	AR2_GAGATTCC-GGCTCTGA_comp17902_seq0	72.757	301	64	2	684	984	207	489	1.60E-44	181
	YL1_CGCTCATT-TATAGCCT_comp661_seq0	66.612	614	174	7	25	621	149	748	5.58E-44	179
CS (MF770512.1)	AR1_GAGATTCC-CCTATCCT_comp5172_seq0	78.417	139	30	0	845	983	13	151	5.58E-25	116
	YL3_CGCTCATT-ATAGAGGC_comp166_seq2	80.234	941	177	3	25	962	311	1245	0	854
	AR1_GAGATTCC-CCTATCCT_comp606_seq1	80	485	94	1	478	962	393	874	4.42E-121	435
	YL1_CGCTCATT-TATAGCCT_comp661_seq0	67.885	903	238	16	8	881	147	1026	2.79E-79	297
	AR2_GAGATTCC-GGCTCTGA_comp17902_seq0	80.351	285	56	0	678	962	207	491	5.76E-69	262
TS (MF770513.1)	YS1_GAGATTCC-TATAGCCT_comp13751_seq2	66.332	597	145	17	28	604	1364	1924	1.11E-33	145
	YL3_CGCTCATT-ATAGAGGC_comp166_seq2	79.277	941	189	3	25	965	311	1245	0	810
	AR1_GAGATTCC-CCTATCCT_comp606_seq1	78.745	494	105	0	472	965	381	874	1.19E-115	417
	YL1_CGCTCATT-TATAGCCT_comp661_seq0	67.037	901	256	12	8	886	147	1028	3.90E-71	269
	AR2_GAGATTCC-GGCTCTGA_comp17902_seq0	79.649	285	58	0	681	965	207	491	3.00E-66	253
VS1 (MF770514.1)	OL1_CGCTCATT-CCTATCCT_comp1318_seq1	66.534	502	135	7	28	523	84	558	3.20E-34	147
	YL3_CGCTCATT-ATAGAGGC_comp166_seq2	80.293	888	171	2	49	935	352	1236	0	807
	AR1_GAGATTCC-CCTATCCT_comp606_seq1	80.206	485	96	0	451	935	381	865	2.92E-123	443
	AR2_GAGATTCC-GGCTCTGA_comp17902_seq0	80.42	286	56	0	660	945	207	492	1.62E-69	264
	YL1_CGCTCATT-TATAGCCT_comp661_seq0	66.469	844	250	8	71	891	227	1060	1.85E-62	241
	YL1_CGCTCATT-TATAGCCT_comp661_seq0	79.487	39	8	0	8	46	147	185	1.4	35.6
VS2 (MF770515.1)	AR1_GAGATTCC-CCTATCCT_comp5172_seq0	81.119	143	27	0	793	935	3	145	5.66E-31	136
	YS2_GAGATTCC-ATAGAGGC_comp528_seq2	77.958	1538	304	6	1	1513	2107	3634	0	1251
	YL1_CGCTCATT-TATAGCCT_comp104_seq6	77.893	1538	305	6	1	1513	2274	3801	0	1245
	YS1_GAGATTCC-TATAGCCT_comp15820_seq1	74.339	1438	334	8	107	1513	305	1738	0	926
	YS2_GAGATTCC-ATAGAGGC_comp37475_seq2	79.321	619	113	1	910	1513	998	1616	4.44E-155	549
T16H1 (FJ647194.1)	OL3_CGCTCATT-GGCTCTGA_comp1489_seq0	68.279	1406	400	17	133	1512	943	2328	3.65E-137	489
	YS2_GAGATTCC-ATAGAGGC_comp528_seq2	78.335	1574	314	9	3	1559	2077	3640	0	1285

TableS1

	YL1_CGCTCATT-TATAGCCT_comp104_seq6	78.272	1574	315	9	3	1559	2244	3807	0	1281
	YS1_GAGATTCC-TATAGCCT_comp15820_seq1	74.054	1453	366	4	113	1558	295	1743	0	913
	YS2_GAGATTCC-ATAGAGGC_comp37475_seq2	81.28	625	117	0	935	1559	998	1622	3.13E-170	600
T16H2 (JF742645.1)	OL3_CGCTCATT-GGCTCTGA_comp1489_seq0	68.355	1441	410	21	143	1569	937	2345	4.08E-137	489
	AR2_GAGATTCC-GGCTCTGA_comp35_seq0	75.923	1084	225	6	60	1128	583	1645	0	783
	YS2_GAGATTCC-ATAGAGGC_comp61_seq0	75.668	1085	241	9	60	1128	2794	3871	0	747
	AR1_GAGATTCC-CCTATCCT_comp1185_seq0	72.913	1078	256	6	60	1128	581	1631	1.15E-180	634
16OMT (EF444544.1)	YS1_GAGATTCC-TATAGCCT_comp1836_seq0	72.146	1095	257	9	49	1128	126	1187	1.94E-171	603
	YS2_GAGATTCC-ATAGAGGC_comp8318_seq1	72.628	917	200	10	1	913	3643	2774	1.77E-147	524
	YS2_GAGATTCC-ATAGAGGC_comp8318_seq1	82.008	239	43	0	913	1151	2478	2240	3.66E-61	237
	YS2_GAGATTCC-ATAGAGGC_comp8318_seq1	90	110	11	0	1325	1434	2237	2128	1.46E-34	149
	YL3_CGCTCATT-ATAGAGGC_comp5333_seq0	75.795	723	163	4	192	913	3124	2413	1.36E-142	508
	YL3_CGCTCATT-ATAGAGGC_comp5333_seq0	79.949	394	63	4	1078	1464	2062	1678	2.31E-95	351
	YS2_GAGATTCC-ATAGAGGC_comp8318_seq2	74.412	723	158	5	192	913	2711	2015	9.79E-132	471
	YS2_GAGATTCC-ATAGAGGC_comp8318_seq2	86.765	204	20	1	1325	1521	1588	1385	2.02E-64	248
	YS2_GAGATTCC-ATAGAGGC_comp8318_seq2	79.73	74	15	0	1078	1151	1664	1591	1.37E-09	66.2
	AR1_GAGATTCC-CCTATCCT_comp629_seq5	67.134	1354	379	26	168	1488	192	1512	1.66E-103	378
T3O (LN831958.1)	AR1_GAGATTCC-CCTATCCT_comp5822_seq1	65.632	1385	433	16	112	1485	237	1589	1.36E-85	318
	AR1_GAGATTCC-CCTATCCT_comp3108_seq0	77.788	1049	226	6	22	1068	189	1232	0	821
	OL1_CGCTCATT-CCTATCCT_comp14114_seq0	74.009	681	158	6	396	1071	591	1257	1.37E-116	421
	AR1_GAGATTCC-CCTATCCT_comp80_seq0	67.191	1018	309	12	67	1067	335	1344	1.13E-79	298
	OL1_CGCTCATT-CCTATCCT_comp1134_seq1	66.437	1016	320	10	67	1067	211	1220	5.48E-71	269
	YL3_CGCTCATT-ATAGAGGC_comp42_seq0	78.71	310	63	1	7	313	160	469	2.84E-68	260
T3R (KP122966.1)	YL3_CGCTCATT-ATAGAGGC_comp42_seq0	66.914	538	170	6	515	1048	680	1213	1.29E-34	149
	YL1_CGCTCATT-TATAGCCT_comp302_seq2	77.549	971	192	15	25	978	211	1172	0	724
	YL1_CGCTCATT-TATAGCCT_comp302_seq2	75.8	781	178	7	25	798	1692	2468	7.06E-151	535
	AR2_GAGATTCC-GGCTCTGA_comp630_seq4	76.369	986	207	15	10	978	1276	2252	0	684
	YS1_GAGATTCC-TATAGCCT_comp198_seq4	75.628	796	183	7	10	798	2351	3142	1.66E-152	540
	OL3_CGCTCATT-GGCTCTGA_comp6929_seq0	73.598	856	217	6	20	869	213	1065	3.43E-142	506
NMT (HM584929.1)	YL1_CGCTCATT-TATAGCCT_comp938_seq4	75.173	721	170	4	153	867	47	764	1.12E-135	484
	AR1_GAGATTCC-CCTATCCT_comp12530_seq0	78.772	1140	229	7	68	1202	54	1185	0	946
	YL3_CGCTCATT-ATAGAGGC_comp5037_seq1	71.826	827	212	10	321	1141	2325	3136	5.21E-115	416
	YL3_CGCTCATT-ATAGAGGC_comp5037_seq1	82	50	9	0	139	188	2143	2192	8.30E-05	50
	YS1_GAGATTCC-TATAGCCT_comp5438_seq0	71.814	816	210	9	332	1141	2238	3039	6.34E-114	412
	YS1_GAGATTCC-TATAGCCT_comp5438_seq0	82	50	9	0	139	188	2046	2095	8.30E-05	50
	AR1_GAGATTCC-CCTATCCT_comp2987_seq0	69.652	804	222	9	332	1127	359	1148	5.95E-89	329
D4H (U71604.1)	OL1_CGCTCATT-CCTATCCT_comp614_seq1	69.406	791	227	7	332	1119	5056	5834	1.08E-85	318
	AR1_GAGATTCC-CCTATCCT_comp10689_seq2	69.337	1298	322	24	31	1314	19	1254	1.18E-142	508
	AR2_GAGATTCC-GGCTCTGA_comp333_seq6	66.023	1295	364	23	31	1314	142	1371	4.12E-85	316
	AR2_GAGATTCC-GGCTCTGA_comp333_seq4	66.023	1295	364	23	31	1314	142	1371	4.12E-85	316
	YS2_GAGATTCC-ATAGAGGC_comp13380_seq0	66.486	555	168	7	202	753	575	1114	4.41E-34	147

TableS1

	YS2_GAGATTCC-ATAGAGGC_comp13380_seq0	69.082	207	52	3	1108	1314	1415	1609	1.54E-14	82.4
	YS2_GAGATTCC-ATAGAGGC_comp13380_seq0	82.857	35	6	0	41	75	54	88	0.57	37.4
DAT (AF053307.1)	AR2_GAGATTCC-GGCTCTGA_comp1084_seq0	63.105	1301	353	34	31	1314	402	1592	5.37E-33	143
	YS2_GAGATTCC-ATAGAGGC_comp528_seq2	69.342	1383	373	21	92	1438	2198	3565	9.78E-151	535
	YS1_GAGATTCC-TATAGCCT_comp15820_seq1	69.136	1377	388	15	94	1438	298	1669	3.41E-150	533
	YL1_CGCTCATT-TATAGCCT_comp104_seq6	69.27	1383	374	21	92	1438	2365	3732	4.16E-149	529
	OL3_CGCTCATT-GGCTCTGA_comp1489_seq0	66.935	1367	413	18	103	1446	919	2269	1.36E-104	381
TEX1 (MG873080.1)	AR2_GAGATTCC-GGCTCTGA_comp863_seq0	66.012	1324	427	12	124	1431	314	1630	9.18E-88	325
	YS2_GAGATTCC-ATAGAGGC_comp528_seq2	69.192	1373	394	15	103	1462	2209	3565	1.48E-148	527
	YL1_CGCTCATT-TATAGCCT_comp104_seq6	69.119	1373	395	15	103	1462	2376	3732	1.80E-147	524
	YS1_GAGATTCC-TATAGCCT_comp15820_seq1	67.632	1347	409	15	124	1455	328	1662	1.58E-116	421
	OL3_CGCTCATT-GGCTCTGA_comp1489_seq0	65.852	1391	412	17	103	1470	919	2269	2.86E-94	347
TEX2 (MG873081.1)	AR2_GAGATTCC-GGCTCTGA_comp863_seq0	65.799	1345	429	9	124	1460	314	1635	4.24E-92	340
	OL3_CGCTCATT-GGCTCTGA_comp1418_seq0	72.105	1520	382	16	1	1505	452	1944	0	796
	OL3_CGCTCATT-GGCTCTGA_comp12875_seq0	65.649	1278	401	21	97	1361	301	1553	7.56E-70	266
	AR1_GAGATTCC-CCTATCCT_comp10373_seq0	68.027	294	92	2	1054	1346	1064	1356	6.64E-20	100
	AR2_GAGATTCC-GGCTCTGA_comp863_seq0	73.81	168	39	3	1207	1370	1388	1554	8.09E-19	96.9
T19H (HQ901597.1)	OL1_CGCTCATT-CCTATCCT_comp520_seq1	70.918	196	56	1	1162	1357	1433	1627	9.86E-18	93.3
	AR1_GAGATTCC-CCTATCCT_comp10689_seq2	69.219	1293	330	19	1	1259	4	1262	2.75E-144	513
	AR2_GAGATTCC-GGCTCTGA_comp333_seq6	69.438	1263	329	22	14	1249	137	1369	3.13E-137	489
	AR2_GAGATTCC-GGCTCTGA_comp333_seq4	69.438	1263	329	22	14	1249	137	1369	3.13E-137	489
	AR2_GAGATTCC-GGCTCTGA_comp1084_seq0	64.969	1276	351	31	13	1261	396	1602	3.83E-60	233
	YS2_GAGATTCC-ATAGAGGC_comp13380_seq0	65.961	1087	317	26	190	1263	575	1621	6.93E-57	223
TAT (KU821123.1)	YS2_GAGATTCC-ATAGAGGC_comp13380_seq0	96.154	26	1	0	418	443	464	489	0.013	42.8
	YS2_GAGATTCC-ATAGAGGC_comp8318_seq1	74.058	902	186	12	19	916	3631	2774	7.04E-159	562
	YS2_GAGATTCC-ATAGAGGC_comp8318_seq1	81.435	237	44	0	916	1152	2478	2242	5.43E-59	230
	YS2_GAGATTCC-ATAGAGGC_comp8318_seq1	88.889	108	12	0	1327	1434	2235	2128	2.17E-32	141
	YL3_CGCTCATT-ATAGAGGC_comp5333_seq0	77.317	723	152	4	195	916	3124	2413	8.57E-158	558
	YL3_CGCTCATT-ATAGAGGC_comp5333_seq0	79.032	372	65	3	1097	1461	2046	1681	1.36E-85	318
	YL3_CGCTCATT-ATAGAGGC_comp5333_seq0	81.356	59	11	0	916	974	2117	2059	7.09E-07	57.2
	YS2_GAGATTCC-ATAGAGGC_comp8318_seq2	75.657	723	149	5	195	916	2711	2015	3.20E-144	513
	YS2_GAGATTCC-ATAGAGGC_comp8318_seq2	85.149	202	23	1	1327	1521	1586	1385	1.56E-59	232
	YS2_GAGATTCC-ATAGAGGC_comp8318_seq2	81.356	59	11	0	916	974	1719	1661	7.09E-07	57.2
	YS2_GAGATTCC-ATAGAGGC_comp8318_seq2	78.571	56	12	0	1097	1152	1648	1593	0.000367	48.2
	AR1_GAGATTCC-CCTATCCT_comp5822_seq1	71.865	622	161	7	868	1485	978	1589	1.66E-84	315
V19H (MK050464.10	YS1_GAGATTCC-TATAGCCT_comp6764_seq2	69.771	698	183	10	805	1488	2776	3459	2.31E-76	288
	AR1_GAGATTCC-CCTATCCT_comp10689_seq2	68.261	1317	321	24	27	1326	18	1254	2.98E-131	470
	AR2_GAGATTCC-GGCTCTGA_comp333_seq6	66.412	1307	354	18	28	1326	142	1371	1.77E-102	374
	AR2_GAGATTCC-GGCTCTGA_comp333_seq4	66.412	1307	354	18	28	1326	142	1371	1.77E-102	374
	YS2_GAGATTCC-ATAGAGGC_comp13380_seq0	68.467	574	163	7	190	760	566	1124	1.89E-51	205
	YS2_GAGATTCC-ATAGAGGC_comp13380_seq0	72.596	208	43	6	1120	1326	1415	1609	4.75E-21	104

	YS2_GAGATTCC-ATAGAGGC_comp13380_seq0	82.857	35	6	0	38	72	54	88	0.58	37.4
	AR2_GAGATTCC-GGCTCTGA_comp1084_seq0	66.725	568	147	12	28	592	402	930	8.59E-37	156
MAT (AF253415.1)	AR2_GAGATTCC-GGCTCTGA_comp1084_seq0	68.722	227	58	4	1102	1326	1377	1592	4.45E-15	84.2
	YL1_CGCTCATT-TATAGCCT_comp302_seq2	98.046	921	18	0	49	969	157	1077	0	1581
	YL1_CGCTCATT-TATAGCCT_comp302_seq2	83.488	969	160	0	1	969	1590	2558	0	1027
	AR2_GAGATTCC-GGCTCTGA_comp630_seq4	97.001	867	26	0	103	969	1291	2157	0	1447
	YS1_GAGATTCC-TATAGCCT_comp198_seq4	80.997	863	161	1	107	969	2373	3232	0	816
	AR1_GAGATTCC-CCTATCCT_comp3145_seq1	84.248	711	97	3	159	866	1	699	0	778
	AR1_GAGATTCC-CCTATCCT_comp3145_seq1	79.048	105	22	0	865	969	815	919	2.17E-17	91.5
VmPiNMT (KC708450	OL3_CGCTCATT-GGCTCTGA_comp6929_seq0	77.855	867	192	0	103	969	218	1084	0	699
**Transporters (TPs):**											
**TP**	**transcript**	**% ID**	**Alignment length**	**Mismatch**	**Gapopen**	**TP start**	**TP end**	**Transcript start**	**Transcript end**	**E-value**	**Bitscore**
	OL3_CGCTCATT-GGCTCTGA_comp2285_seq1	77.333	1725	381	6	192	1914	713	2429	0	1330
	OL3_CGCTCATT-GGCTCTGA_comp2285_seq1	87.179	156	20	0	239	394	401	556	1.73E-47	192
	OL3_CGCTCATT-GGCTCTGA_comp2285_seq1	78.431	51	11	0	192	242	160	210	0.02	42.8
	OL1_CGCTCATT-CCTATCCT_comp26741_seq0	76.236	1456	337	3	459	1914	1472	2918	0	1061
	YL1_CGCTCATT-TATAGCCT_comp4178_seq2	75.326	1459	351	3	459	1914	1452	2904	0	1003
	YL1_CGCTCATT-TATAGCCT_comp3070_seq2	77.114	887	200	1	1031	1914	2041	2927	0	682
	YL1_CGCTCATT-TATAGCCT_comp3070_seq2	76.014	838	195	2	202	1039	288	1119	1.01E-170	601
NPF2.1 (KR054375.1)	YS2_GAGATTCC-ATAGAGGC_comp11749_seq1	75.369	881	214	1	1034	1914	1179	2056	6.83E-173	609
	OL3_CGCTCATT-GGCTCTGA_comp2285_seq1	77.493	1755	377	10	96	1844	683	2425	0	1355
	OL3_CGCTCATT-GGCTCTGA_comp2285_seq1	83.117	154	26	0	173	326	401	554	2.82E-38	161
	OL3_CGCTCATT-GGCTCTGA_comp2285_seq1	79.012	81	17	0	96	176	130	210	1.37E-10	69.8
	OL1_CGCTCATT-CCTATCCT_comp26741_seq0	76.823	1454	328	4	393	1846	1472	2916	0	1094
	YL1_CGCTCATT-TATAGCCT_comp4178_seq2	75.585	1454	352	2	393	1846	1452	2902	0	1016
	YL1_CGCTCATT-TATAGCCT_comp3070_seq2	75.312	960	214	7	18	973	179	1119	0	652
	YL1_CGCTCATT-TATAGCCT_comp3070_seq2	76.303	844	200	0	965	1808	2041	2884	1.04E-176	621
NPF2.2 (KR054376.1)	YS2_GAGATTCC-ATAGAGGC_comp11749_seq1	76.109	879	207	2	968	1846	1179	2054	5.77E-180	632
	OL3_CGCTCATT-GGCTCTGA_comp2285_seq1	77.803	1784	366	6	128	1908	673	2429	0	1433
	OL3_CGCTCATT-GGCTCTGA_comp2285_seq1	84.416	154	24	0	218	371	401	554	5.62E-41	170
	OL3_CGCTCATT-GGCTCTGA_comp2285_seq1	72.165	97	18	3	128	221	120	210	0.000462	48.2
	YL1_CGCTCATT-TATAGCCT_comp4178_seq2	79.691	1423	277	2	438	1854	1452	2868	0	1265
	OL1_CGCTCATT-CCTATCCT_comp26741_seq0	79.534	1417	284	1	438	1854	1472	2882	0	1249
	YL1_CGCTCATT-TATAGCCT_comp3070_seq2	81.758	899	152	3	1010	1908	2041	2927	0	879
	YL1_CGCTCATT-TATAGCCT_comp3070_seq2	81.229	911	156	4	111	1018	221	1119	0	868
NPF2.3 (KR054377.1)	YS2_GAGATTCC-ATAGAGGC_comp11749_seq1	80.048	842	168	0	1013	1854	1179	2020	0	762
	OL3_CGCTCATT-GGCTCTGA_comp8789_seq0	82.886	1788	291	6	1	1788	109	1881	0	1831
	YL3_CGCTCATT-ATAGAGGC_comp13973_seq0	69.693	1630	418	20	106	1717	519	2090	0	691
	YL3_CGCTCATT-ATAGAGGC_comp13973_seq0	78.218	303	66	0	79	381	185	487	6.84E-65	250
	YS2_GAGATTCC-ATAGAGGC_comp3442_seq2	68.611	1663	499	11	64	1716	335	1984	1.23E-175	618
	AR2_GAGATTCC-GGCTCTGA_comp6824_seq0	68.163	1671	492	11	55	1717	156	1794	9.46E-171	601

	YL3_CGCTCATT-ATAGAGGC_comp3976_seq4	71.411	829	225	5	58	880	289	1111	7.78E-115	416
NPF2.4 (KR054378.1)	YL3_CGCTCATT-ATAGAGGC_comp3976_seq4	66.026	833	277	4	887	1716	1636	2465	4.92E-54	214
	YL3_CGCTCATT-ATAGAGGC_comp13973_seq0	79.799	1688	286	9	86	1767	517	2155	0	1516
	YL3_CGCTCATT-ATAGAGGC_comp13973_seq0	87.047	386	47	1	6	391	133	515	3.98E-131	470
	OL3_CGCTCATT-GGCTCTGA_comp8789_seq0	70.662	1677	456	12	40	1701	163	1818	0	785
	YS2_GAGATTCC-ATAGAGGC_comp3442_seq2	69.952	1674	469	13	46	1705	335	1988	0	724
	YL3_CGCTCATT-ATAGAGGC_comp3976_seq4	76.761	809	183	3	6	813	258	1062	2.68E-171	603
	YL3_CGCTCATT-ATAGAGGC_comp3976_seq4	66.627	851	267	7	865	1705	1626	2469	1.22E-61	239
NPF2.5 (KR054379.1)	AR2_GAGATTCC-GGCTCTGA_comp6824_seq0	68.378	1714	487	22	40	1736	159	1834	1.14E-169	598
	AR2_GAGATTCC-GGCTCTGA_comp6824_seq0	86.18	1780	231	2	1	1780	93	1857	0	2105
	OL3_CGCTCATT-GGCTCTGA_comp8789_seq0	68.645	1668	503	14	64	1718	160	1820	7.74E-172	605
	YL3_CGCTCATT-ATAGAGGC_comp13973_seq0	68.741	1398	393	12	113	1499	517	1881	3.29E-151	536
	YL3_CGCTCATT-ATAGAGGC_comp13973_seq0	74.32	331	83	2	56	385	153	482	7.28E-52	206
	YS2_GAGATTCC-ATAGAGGC_comp3442_seq2	67.629	1662	511	16	72	1717	334	1984	2.08E-147	524
NPF2.6 (KR054380.1)	YL1_CGCTCATT-TATAGCCT_comp5423_seq0	85.583	326	47	0	1	326	74	399	6.80E-103	376
	YS2_GAGATTCC-ATAGAGGC_comp3442_seq2	86.403	1743	234	1	37	1779	308	2047	0	2073
	YL3_CGCTCATT-ATAGAGGC_comp3976_seq4	84.802	908	135	1	872	1779	1624	2528	0	1014
	YL3_CGCTCATT-ATAGAGGC_comp3976_seq4	84.54	815	123	2	64	875	295	1109	0	895
	OL3_CGCTCATT-GGCTCTGA_comp8789_seq0	69.424	1668	469	16	64	1712	169	1814	0	673
	YL3_CGCTCATT-ATAGAGGC_comp13973_seq0	68.54	1548	425	12	104	1640	517	2013	1.15E-169	598
	YL3_CGCTCATT-ATAGAGGC_comp13973_seq0	78.246	285	62	0	64	348	170	454	1.50E-60	235
NPF2.7 (KR054381.1)	AR2_GAGATTCC-GGCTCTGA_comp6824_seq0	67.351	1657	503	12	63	1712	164	1789	4.88E-149	529
	YS2_GAGATTCC-ATAGAGGC_comp3442_seq2	79.965	1727	330	6	76	1797	332	2047	0	1543
	YL3_CGCTCATT-ATAGAGGC_comp3976_seq4	77.133	914	197	4	887	1797	1624	2528	0	699
	YL3_CGCTCATT-ATAGAGGC_comp3976_seq4	79.075	822	161	5	79	893	295	1112	0	695
	OL3_CGCTCATT-GGCTCTGA_comp8789_seq0	66.707	1670	515	19	79	1729	169	1816	2.55E-127	457
	YL3_CGCTCATT-ATAGAGGC_comp13973_seq0	66.338	1625	479	18	119	1729	517	2087	1.61E-123	444
	YL3_CGCTCATT-ATAGAGGC_comp13973_seq0	75.09	277	67	2	88	363	179	454	2.93E-44	181
	AR2_GAGATTCC-GGCTCTGA_comp6824_seq0	69.159	963	265	19	78	1027	164	1107	1.42E-92	342
NPF2.8 (KR054382.1)	AR2_GAGATTCC-GGCTCTGA_comp6824_seq0	63.089	382	126	3	1387	1768	1464	1830	1.02E-05	53.6
	YL3_CGCTCATT-ATAGAGGC_comp2760_seq1	78.218	1717	351	5	1	1716	254	1948	0	1409
	YL1_CGCTCATT-TATAGCCT_comp789_seq5	74.941	850	198	2	867	1716	3127	3961	1.03E-163	578
	YL1_CGCTCATT-TATAGCCT_comp789_seq6	74.706	850	200	2	867	1716	3966	4800	5.36E-161	569
	YL1_CGCTCATT-TATAGCCT_comp789_seq0	74.706	850	200	2	867	1716	3922	4756	5.36E-161	569
	YL1_CGCTCATT-TATAGCCT_comp28142_seq0	74.441	313	80	0	1404	1716	158	470	2.45E-51	205
	YL1_CGCTCATT-TATAGCCT_comp28142_seq0	83.803	142	23	0	829	970	15	156	1.35E-35	152
NPF2.9 (KX372303.1)	YL1_CGCTCATT-TATAGCCT_comp28142_seq0	76	75	18	0	1642	1716	495	569	2.80E-06	55.4
	AR1_GAGATTCC-CCTATCCT_comp18788_seq0	82.497	1554	260	3	1	1554	192	1733	0	1573
	OL1_CGCTCATT-CCTATCCT_comp74_seq2	81.778	1575	275	3	1	1575	2257	3819	0	1544
	YS1_GAGATTCC-TATAGCCT_comp122_seq0	81.651	1575	277	3	1	1575	1509	3071	0	1535
	OL3_CGCTCATT-GGCTCTGA_comp1979_seq2	81.587	1575	278	3	1	1575	936	2498	0	1530

TableS1

MATE1 (KX372304.1)	OL1_CGCTCATT-CCTATCCT_comp7876_seq0	83.482	1235	204	0	320	1554	3127	4361	0	1308
	YS2_GAGATTCC-ATAGAGGC_comp3345_seq1	82.43	1383	237	3	112	1491	308	1687	0	1391
	YS2_GAGATTCC-ATAGAGGC_comp3345_seq1	85.714	35	5	0	9	43	229	263	0.053	41
	YS1_GAGATTCC-TATAGCCT_comp124_seq0	82.43	1383	237	3	112	1491	2469	3848	0	1391
	YS1_GAGATTCC-TATAGCCT_comp124_seq0	84.615	39	6	0	5	43	2386	2424	0.004	44.6
	YL3_CGCTCATT-ATAGAGGC_comp598_seq6	82.357	1383	238	3	112	1491	2895	4274	0	1386
	YL3_CGCTCATT-ATAGAGGC_comp598_seq6	84.615	39	6	0	5	43	2812	2850	0.004	44.6
	YS2_GAGATTCC-ATAGAGGC_comp3345_seq7	82.357	1383	238	3	112	1491	311	1690	0	1386
	YS2_GAGATTCC-ATAGAGGC_comp3345_seq7	85.714	35	5	0	9	43	229	263	0.053	41
	AR1_GAGATTCC-CCTATCCT_comp14782_seq0	76.637	672	154	1	820	1491	941	1609	5.66E-141	502
MATE2 (KX372305.1)	AR1_GAGATTCC-CCTATCCT_comp14782_seq0	80	40	8	0	320	359	441	480	0.65	37.4
	OL3_CGCTCATT-GGCTCTGA_comp2267_seq1	99.184	4044	32	1	1	4044	1547	5589	0	7142
	YL1_CGCTCATT-TATAGCCT_comp818_seq3	78.995	1771	372	0	2274	4044	2892	4662	0	1517
	YL1_CGCTCATT-TATAGCCT_comp818_seq3	70.379	2245	628	17	40	2270	593	2814	0	1009
	YS2_GAGATTCC-ATAGAGGC_comp1760_seq0	71.981	2873	768	17	40	2898	579	3428	0	1510
	YS2_GAGATTCC-ATAGAGGC_comp1760_seq0	75.32	859	212	0	2908	3766	3815	4673	3.20E-168	594
	YS2_GAGATTCC-ATAGAGGC_comp1760_seq0	82.927	82	14	0	3771	3852	4935	5016	3.93E-15	86
	YS1_GAGATTCC-TATAGCCT_comp1672_seq3	71.911	2873	770	17	40	2898	608	3457	0	1501
	YS1_GAGATTCC-TATAGCCT_comp1672_seq3	75.661	945	230	0	2908	3852	3901	4845	0	668
	OL1_CGCTCATT-CCTATCCT_comp1940_seq1	71.536	2252	613	12	655	2898	181	2412	0	1144
	OL1_CGCTCATT-CCTATCCT_comp1940_seq1	75.443	790	194	0	2977	3766	3027	3816	3.42E-155	551
	OL1_CGCTCATT-CCTATCCT_comp1940_seq1	81.707	82	15	0	3771	3852	4078	4159	1.67E-13	80.6
VmTPT2 (KC511773.1	OL1_CGCTCATT-CCTATCCT_comp1940_seq1	80.263	76	15	0	2908	2983	2853	2928	3.02E-10	69.8
	OL3_CGCTCATT-GGCTCTGA_comp2267_seq1	87.305	4230	534	1	37	4266	1363	5589	0	5205
	YS2_GAGATTCC-ATAGAGGC_comp1760_seq0	71.399	2867	780	17	268	3120	588	3428	0	1433
	YS2_GAGATTCC-ATAGAGGC_comp1760_seq0	75.086	871	215	2	3119	3988	3804	4673	1.75E-165	585
	YS2_GAGATTCC-ATAGAGGC_comp1760_seq0	81.053	95	18	0	3993	4087	4935	5029	9.75E-17	91.5
	YS1_GAGATTCC-TATAGCCT_comp1672_seq3	71.259	2867	784	17	268	3120	617	3457	0	1415
	YS1_GAGATTCC-TATAGCCT_comp1672_seq3	75.67	970	234	2	3119	4087	3890	4858	0	679
	YL1_CGCTCATT-TATAGCCT_comp818_seq3	77.314	1772	400	2	2496	4266	2892	4662	0	1377
	YL1_CGCTCATT-TATAGCCT_comp818_seq3	71.251	2014	562	9	268	2270	602	2609	0	996
	YL1_CGCTCATT-TATAGCCT_comp818_seq3	75	68	17	0	2425	2492	2747	2814	0.004	46.4
	OL1_CGCTCATT-CCTATCCT_comp1940_seq1	71.188	2256	617	15	874	3120	181	2412	0	1101
	OL1_CGCTCATT-CCTATCCT_comp1940_seq1	75.601	791	191	2	3199	3988	3027	3816	1.26E-154	549
	OL1_CGCTCATT-CCTATCCT_comp1940_seq1	88.06	67	8	0	3993	4059	4078	4144	4.14E-15	86
CrTPT2 (KC511771.1)	OL1_CGCTCATT-CCTATCCT_comp1940_seq1	77.011	87	20	0	3119	3205	2842	2928	1.11E-09	68
**Transcription Factors (TFs):**											
**TF**	**transcript**	**% ID**	**Alignment length**	**Mismatch**	**Gapopen**	**TF start**	**TF end**	**Transcript start**	**Transcript end**	**E-value**	**Bitscore**
	OL3_CGCTCATT-GGCTCTGA_comp9977_seq1	78.509	228	49	0	365	592	662	889	1.93E-47	190
	YL1_CGCTCATT-TATAGCCT_comp6621_seq0	74.308	253	59	2	365	611	688	940	3.27E-38	159

TableS1

	YS2_GAGATTCC-ATAGAGGC_comp27339_seq0	74.336	226	45	1	367	592	422	634	1.39E-36	154
	YS2_GAGATTCC-ATAGAGGC_comp27339_seq0	91.429	35	3	0	76	110	134	168	4.27E-05	50
	AR2_GAGATTCC-GGCTCTGA_comp3513_seq0	75.568	176	43	0	376	551	496	671	2.36E-27	123
ORCA2 (AJ238740.1)	YS1_GAGATTCC-TATAGCCT_comp5858_seq0	73.988	173	45	0	379	551	616	788	5.19E-23	109
	YL1_CGCTCATT-TATAGCCT_comp6621_seq0	80.669	269	49	1	1085	1353	666	931	6.95E-65	250
	YL1_CGCTCATT-TATAGCCT_comp6621_seq0	76.316	76	18	0	837	912	124	199	8.49E-07	57.2
	AR2_GAGATTCC-GGCTCTGA_comp3513_seq0	84.615	182	28	0	1122	1303	497	678	9.05E-51	203
	OL3_CGCTCATT-GGCTCTGA_comp9977_seq1	78.947	209	44	0	1104	1312	656	864	1.03E-43	179
	OL3_CGCTCATT-GGCTCTGA_comp9977_seq1	78.481	79	14	2	823	898	33	111	6.97E-08	60.8
	OL3_CGCTCATT-GGCTCTGA_comp9977_seq1	75	48	12	0	991	1038	345	392	9.7	33.7
	AR1_GAGATTCC-CCTATCCT_comp14477_seq0	83.333	138	23	0	1112	1249	458	595	2.13E-33	145
ORCA3 (AJ251250.1)	AR1_GAGATTCC-CCTATCCT_comp11153_seq4	73.14	242	62	1	1145	1386	435	673	2.59E-32	141
	YL1_CGCTCATT-TATAGCCT_comp6621_seq0	78.516	256	55	0	340	595	675	930	1.80E-54	214
	YL1_CGCTCATT-TATAGCCT_comp6621_seq0	81.928	83	10	3	16	95	126	206	3.98E-12	73.4
	AR2_GAGATTCC-GGCTCTGA_comp3513_seq0	84.066	182	29	0	365	546	497	678	1.39E-49	197
	AR1_GAGATTCC-CCTATCCT_comp11153_seq4	77.542	236	51	2	361	595	408	642	3.72E-44	179
	OL3_CGCTCATT-GGCTCTGA_comp9977_seq1	74.429	219	55	1	337	554	645	863	3.26E-32	140
	OL3_CGCTCATT-GGCTCTGA_comp9977_seq1	86.667	60	8	0	19	78	55	114	3.98E-12	73.4
ORCA4 (KR703577.1	AR1_GAGATTCC-CCTATCCT_comp14477_seq0	80.142	141	28	0	355	495	458	598	5.90E-29	129
	AR2_GAGATTCC-GGCTCTGA_comp3513_seq0	81.191	319	51	2	614	932	488	797	1.83E-81	304
	AR2_GAGATTCC-GGCTCTGA_comp3513_seq0	78.75	80	14	1	25	101	178	257	8.33E-10	66.2
	AR1_GAGATTCC-CCTATCCT_comp14477_seq0	81.503	173	29	1	584	753	426	598	3.31E-40	167
	AR1_GAGATTCC-CCTATCCT_comp14477_seq0	80.723	83	10	1	25	101	146	228	1.32E-13	78.8
	YL1_CGCTCATT-TATAGCCT_comp6621_seq0	81.437	167	31	0	631	797	708	874	1.41E-38	161
	OL3_CGCTCATT-GGCTCTGA_comp9977_seq1	81.679	131	24	0	631	761	682	812	8.32E-29	129
ORCA5 (KR703578.1)	YL1_CGCTCATT-TATAGCCT_comp10613_seq0	72.989	174	47	0	624	797	554	727	1.16E-20	102
	AR2_GAGATTCC-GGCTCTGA_comp3513_seq0	82.162	185	33	0	271	455	487	671	7.12E-46	185
	AR1_GAGATTCC-CCTATCCT_comp14477_seq0	80.952	147	28	0	271	417	458	604	2.66E-32	140
	OL3_CGCTCATT-GGCTCTGA_comp9977_seq1	74.869	191	48	0	281	471	674	864	4.80E-29	129
	YS1_GAGATTCC-TATAGCCT_comp5858_seq0	74.566	173	44	0	283	455	616	788	1.06E-24	114
	YS2_GAGATTCC-ATAGAGGC_comp27339_seq0	70.244	205	48	2	267	471	418	609	8.13E-20	98.7
ORCA6 (MN614455.1	YS2_GAGATTCC-ATAGAGGC_comp27339_seq0	85.714	28	4	0	126	153	235	262	2.8	33.7
	YL3_CGCTCATT-ATAGAGGC_comp4742_seq3	73.982	1130	199	22	3	1101	269	1334	0	699
	AR1_GAGATTCC-CCTATCCT_comp8525_seq2	74.291	564	133	4	511	1071	750	1304	3.90E-97	356
	AR1_GAGATTCC-CCTATCCT_comp8525_seq2	78.947	114	19	2	7	116	285	397	7.07E-18	93.3
	OL1_CGCTCATT-CCTATCCT_comp6055_seq0	65.82	433	128	7	622	1038	880	1308	4.76E-20	100
	AR2_GAGATTCC-GGCTCTGA_comp1597_seq3	78.947	114	19	2	7	116	300	412	7.07E-18	93.3
BIS1 (KM409646.2)	YL1_CGCTCATT-TATAGCCT_comp10816_seq0	93.548	31	1	1	859	888	1680	1650	0.003	44.6
	AR1_GAGATTCC-CCTATCCT_comp8525_seq2	70.934	867	152	15	372	1230	551	1325	2.60E-125	450
	AR1_GAGATTCC-CCTATCCT_comp8525_seq2	69.811	159	24	4	1	159	282	416	2.15E-12	75.2
	YL3_CGCTCATT-ATAGAGGC_comp4742_seq3	71.575	584	142	5	631	1214	750	1309	2.44E-81	304

	YL3_CGCTCATT-ATAGAGGC_comp4742_seq3	72.857	70	7	3	9	78	275	332	0.15	39.2
	OL1_CGCTCATT-CCTATCCT_comp6055_seq0	71.171	222	64	0	662	883	809	1030	8.54E-24	113
	OL1_CGCTCATT-CCTATCCT_comp6055_seq0	67.485	163	53	0	1013	1175	1145	1307	2.01E-06	55.4
	AR2_GAGATTCC-GGCTCTGA_comp1597_seq3	69.811	159	24	4	1	159	297	431	2.15E-12	75.2
	AR2_GAGATTCC-GGCTCTGA_comp1597_seq3	76	100	15	3	372	468	566	659	3.19E-10	68
	AR2_GAGATTCC-GGCTCTGA_comp1597_seq3	83.871	31	5	0	215	245	466	496	6.6	33.7
BIS2 (KM409645.1)	AR2_GAGATTCC-GGCTCTGA_comp1660_seq1	80.952	63	12	0	757	819	891	953	4.73E-08	60.8
	OL3_CGCTCATT-GGCTCTGA_comp2316_seq0	80	1415	181	27	1158	2552	1441	2773	0	1247
	OL1_CGCTCATT-CCTATCCT_comp4812_seq0	80.783	1353	191	24	1185	2501	1078	2397	0	1222
	OL1_CGCTCATT-CCTATCCT_comp4812_seq0	70.926	1080	191	21	77	1076	8	1044	7.71E-155	549
	OL1_CGCTCATT-CCTATCCT_comp2168_seq4	74.927	1029	181	18	77	1079	107	1084	0	681
	YS1_GAGATTCC-TATAGCCT_comp2461_seq6	74.636	1029	184	18	77	1079	204	1181	0	668
	YS1_GAGATTCC-TATAGCCT_comp14327_seq2	80.18	666	91	18	1895	2540	3	647	1.22E-158	562
	YS1_GAGATTCC-TATAGCCT_comp14327_seq2	76.087	92	8	5	2261	2350	655	734	1.23E-06	57.2
MYC2 (AF283507.2)	YS1_GAGATTCC-TATAGCCT_comp14327_seq2	73.529	102	16	5	2450	2547	735	829	0.000183	50
	AR1_GAGATTCC-CCTATCCT_comp24292_seq0	77.204	329	51	6	322	632	542	864	2.93E-65	250
	YS1_GAGATTCC-TATAGCCT_comp87_seq4	71.498	414	85	8	30	425	154	552	8.96E-53	208
	YS1_GAGATTCC-TATAGCCT_comp87_seq3	71.294	425	83	10	30	427	154	566	3.13E-52	206
	AR1_GAGATTCC-CCTATCCT_comp16215_seq2	77.533	227	30	7	424	632	808	1031	2.74E-40	167
WRKY1 (HQ646368.1	AR2_GAGATTCC-GGCTCTGA_comp1658_seq0	75.771	227	31	6	424	632	3831	4051	4.96E-37	156
	AR1_GAGATTCC-CCTATCCT_comp737_seq2	72.233	533	94	14	104	600	328	842	1.76E-74	280
	AR1_GAGATTCC-CCTATCCT_comp737_seq2	96.97	33	1	0	70	102	267	299	1.27E-06	55.4
	YL1_CGCTCATT-TATAGCCT_comp10081_seq1	69.458	203	56	2	206	405	179	378	2.62E-15	84.2
	AR2_GAGATTCC-GGCTCTGA_comp7502_seq0	70.408	196	53	4	203	397	261	452	1.11E-13	78.8
	AR2_GAGATTCC-GGCTCTGA_comp7502_seq2	68.837	215	52	5	194	396	240	451	3.89E-13	77
ZCT1 (AJ632082.1)	AR1_GAGATTCC-CCTATCCT_comp6246_seq5	78.75	80	15	2	200	278	527	605	2.99E-08	60.8
	AR2_GAGATTCC-GGCTCTGA_comp7502_seq0	69.737	608	124	15	80	657	90	667	3.09E-66	253
	AR2_GAGATTCC-GGCTCTGA_comp7502_seq0	73.75	160	29	9	805	958	840	992	2.09E-11	71.6
	AR2_GAGATTCC-GGCTCTGA_comp7502_seq2	66.026	624	111	19	64	657	76	628	6.82E-43	176
	AR2_GAGATTCC-GGCTCTGA_comp7502_seq2	71.519	158	27	9	808	958	802	948	1.32E-07	59
	AR1_GAGATTCC-CCTATCCT_comp737_seq2	71.689	219	51	5	236	447	409	623	3.54E-21	104
	AR2_GAGATTCC-GGCTCTGA_comp5665_seq0	80.556	72	14	0	379	450	1288	1359	2.55E-10	68
	AR2_GAGATTCC-GGCTCTGA_comp5665_seq0	71.053	76	22	0	238	313	1096	1171	0.12	39.2
ZCT2 (AJ632083.1)	YL1_CGCTCATT-TATAGCCT_comp10081_seq1	73.737	99	26	0	214	312	145	243	1.08E-08	62.6
	AR1_GAGATTCC-CCTATCCT_comp521_seq0	72.048	991	174	23	61	1023	171	1086	9.68E-149	527
	AR2_GAGATTCC-GGCTCTGA_comp1098_seq4	71.287	1010	166	28	61	1019	1556	2492	1.35E-140	500
	OL3_CGCTCATT-GGCTCTGA_comp7752_seq4	71.125	987	179	25	57	998	186	1111	4.40E-134	479
	OL1_CGCTCATT-CCTATCCT_comp2136_seq4	69.679	343	63	13	676	1005	926	1240	9.12E-29	129
ZCT3 (AJ632084.1)	AR2_GAGATTCC-GGCTCTGA_comp10369_seq0	75.862	87	15	1	679	765	481	401	3.19E-09	64.4
	YL1_CGCTCATT-TATAGCCT_comp1354_seq1	68.293	1066	143	29	1	1042	103	997	9.27E-124	444
	YS2_GAGATTCC-ATAGAGGC_comp2064_seq8	67.864	1058	146	26	1	1042	2965	3844	1.68E-120	434

TableS1

	OL3_CGCTCATT-GGCTCTGA_comp8246_seq1	67.628	1075	149	31	1	1044	49	955	1.91E-113	410
	YS1_GAGATTCC-TATAGCCT_comp2284_seq2	68.045	1064	149	35	1	1042	119	1013	9.91E-111	401
	YS2_GAGATTCC-ATAGAGGC_comp4735_seq0	73.786	515	81	9	539	1044	326	795	5.14E-89	329
GATA1 (MK801106.1)	YS2_GAGATTCC-ATAGAGGC_comp4735_seq0	91.304	23	2	0	83	105	181	203	5.5	33.7
	YS1_GAGATTCC-TATAGCCT_comp3725_seq0	77.524	1882	300	38	5	1858	186	1972	0	1427
	YS1_GAGATTCC-TATAGCCT_comp3725_seq0	100	19	0	0	1	19	168	186	2.9	35.6
	YL1_CGCTCATT-TATAGCCT_comp4560_seq1	80.074	1345	215	17	5	1320	130	1450	0	1189
	YL1_CGCTCATT-TATAGCCT_comp4560_seq1	72.176	478	66	20	1386	1858	1716	2131	5.51E-60	233
	YL1_CGCTCATT-TATAGCCT_comp4560_seq1	100	19	0	0	1	19	112	130	2.9	35.6
	YL1_CGCTCATT-TATAGCCT_comp4560_seq0	78.753	1219	199	12	6	1205	112	1289	0	1031
	YL1_CGCTCATT-TATAGCCT_comp4560_seq0	71.084	664	107	22	1203	1858	1393	1979	8.73E-83	309
	OL1_CGCTCATT-CCTATCCT_comp4376_seq4	81.124	694	116	2	621	1314	849	1527	0	666
	OL1_CGCTCATT-CCTATCCT_comp4376_seq4	77.671	627	98	10	6	613	125	728	3.70E-138	493
	OL1_CGCTCATT-CCTATCCT_comp4376_seq4	69.933	449	78	17	1380	1825	1668	2062	1.30E-42	176
GBF1 (AF084971.1)	YL1_CGCTCATT-TATAGCCT_comp4162_seq2	75.269	93	23	0	1118	1210	1082	1174	5.90E-09	64.4
	OL3_CGCTCATT-GGCTCTGA_comp5631_seq2	81.358	1797	232	28	22	1786	124	1849	0	1698
	AR1_GAGATTCC-CCTATCCT_comp6026_seq0	83.374	1215	146	18	574	1769	706	1883	0	1251
	AR1_GAGATTCC-CCTATCCT_comp6026_seq0	85.985	264	32	2	355	618	398	656	1.08E-81	306
	AR1_GAGATTCC-CCTATCCT_comp6026_seq0	69.424	278	48	10	22	289	138	388	5.61E-22	107
	AR2_GAGATTCC-GGCTCTGA_comp6592_seq4	82.512	1258	152	21	574	1803	761	1978	0	1243
	AR2_GAGATTCC-GGCTCTGA_comp6592_seq4	77.758	571	78	14	22	582	146	677	7.27E-122	439
	YS1_GAGATTCC-TATAGCCT_comp5460_seq5	84.724	995	119	8	188	1163	234	1214	0	1103
	YS1_GAGATTCC-TATAGCCT_comp5460_seq5	80.787	635	79	15	1160	1786	2377	2976	2.08E-160	567
	YS1_GAGATTCC-TATAGCCT_comp5460_seq5	73.196	97	23	2	5	100	83	177	3.79E-05	51.8
	YS1_GAGATTCC-TATAGCCT_comp5460_seq5	81.818	44	8	0	82	125	180	223	0.006	44.6
	AR1_GAGATTCC-CCTATCCT_comp11579_seq0	82.381	840	109	15	866	1686	1895	2714	0	816
	AR1_GAGATTCC-CCTATCCT_comp11579_seq0	92.069	290	23	0	574	863	738	1027	6.81E-116	419
GBF2 (AF084972.1)	AR1_GAGATTCC-CCTATCCT_comp11579_seq0	75.207	605	81	16	18	582	78	653	1.50E-111	405
	AR2_GAGATTCC-GGCTCTGA_comp630_seq3	78.341	651	78	11	385	1026	424	1020	3.62E-154	545
	AR2_GAGATTCC-GGCTCTGA_comp630_seq3	82.692	104	15	1	1	101	157	260	1.04E-21	105
	AR2_GAGATTCC-GGCTCTGA_comp630_seq3	82.051	78	14	0	236	313	323	400	1.45E-13	78.8
	AR2_GAGATTCC-GGCTCTGA_comp630_seq4	78.341	651	78	11	385	1026	424	1020	3.62E-154	545
	AR2_GAGATTCC-GGCTCTGA_comp630_seq4	82.692	104	15	1	1	101	157	260	1.04E-21	105
	AR2_GAGATTCC-GGCTCTGA_comp630_seq4	82.051	78	14	0	236	313	323	400	1.45E-13	78.8
	AR2_GAGATTCC-GGCTCTGA_comp630_seq1	78.221	652	79	11	384	1026	420	1017	1.26E-153	544
	AR2_GAGATTCC-GGCTCTGA_comp630_seq1	82.692	104	15	1	1	101	157	260	1.04E-21	105
	AR2_GAGATTCC-GGCTCTGA_comp630_seq1	80	95	19	0	236	330	323	417	9.77E-16	86
	OL1_CGCTCATT-CCTATCCT_comp1173_seq1	78.034	651	80	11	385	1026	483	1079	1.88E-151	536
	OL1_CGCTCATT-CCTATCCT_comp1173_seq1	82.692	104	15	1	1	101	219	322	1.04E-21	105
	OL1_CGCTCATT-CCTATCCT_comp1173_seq1	79.487	78	16	0	236	313	382	459	7.51E-11	69.8
	YL3_CGCTCATT-ATAGAGGC_comp915_seq2	77.914	652	81	11	384	1026	630	1227	6.55E-151	535

TableS1

CrERF5 (MK862158.1	YL3_CGCTCATT-ATAGAGGC_comp915_seq2	80.769	78	15	0	253	330	550	627	6.17E-12	73.4

TableS2

Transcript ID	MIA	Communities	sprot_Top_BLASTX_hit
YS2_GAGATTCC-ATAGAGGC_comp13351_seq1	NA	1	Y3037_ARATH^Y3037_ARATH^Q:412-2247,H:43-639^42.86%ID^E:4e-128^RecName: Full=Probable inactive leucine-rich repeat receptor-like protein kinase At3g03770
OL3_CGCTCATT-GGCTCTGA_comp10301_seq0	NA	1	.
YL1_CGCTCATT-TATAGCCT_comp2586_seq1	NA	1	.
YL3_CGCTCATT-ATAGAGGC_comp19793_seq1	NA	1	C86A2_ARATH^C86A2_ARATH^Q:2-346,H:409-523^75.65%ID^E:2e-53^RecName: Full=Cytochrome P450 86A2
YS2_GAGATTCC-ATAGAGGC_comp1135_seq0	NA	1	RL212_ARATH^RL212_ARATH^Q:27-518,H:1-164^84.76%ID^E:7e-100^RecName: Full=60S ribosomal protein L21-2
AR1_GAGATTCC-CCTATCCT_comp8017_seq0	NA	1	DST1_DICDI^DST1_DICDI^Q:863-1696,H:15-283^57.55%ID^E:2e-97^RecName: Full=Serine/threonine-protein kinase dst1 {ECO:0000250|UniProtKB:O61125}
YL3_CGCTCATT-ATAGAGGC_comp3883_seq1	NA	1	F135B_XENLA^F135B_XENLA^Q:2876-3694,H:1102-1362^41.09%ID^E:5e-51^RecName: Full=Protein FAM135B
OL1_CGCTCATT-CCTATCCT_comp1577_seq4	NA	1	GLGL1_SOLTU^GLGL1_SOLTU^Q:941-2191,H:2-417^81.29%ID^E:0^RecName: Full=Glucose-1-phosphate adenylyltransferase large subunit 1
YS2_GAGATTCC-ATAGAGGC_comp561_seq1	NA	1	C81F1_ARATH^C81F1_ARATH^Q:172-1560,H:39-499^40.88%ID^E:3e-118^RecName: Full=Cytochrome P450 81F1 {ECO:0000305}
YS1_GAGATTCC-TATAGCCT_comp8849_seq1	NA	1	FDL1_ARATH^FDL1_ARATH^Q:168-1520,H:3-415^28.14%ID^E:1e-29^RecName: Full=F-box/FBD/LRR-repeat protein At1g13570
YL3_CGCTCATT-ATAGAGGC_comp188_seq0	NA	1	BGL17_ARATH^BGL17_ARATH^Q:1932-3023,H:33-395^56.87%ID^E:4e-135^RecName: Full=Beta-glucosidase 17
YS1_GAGATTCC-TATAGCCT_comp2178_seq1	NA	1	.
OL1_CGCTCATT-CCTATCCT_comp2611_seq3	NA	1	.
YL3_CGCTCATT-ATAGAGGC_comp7990_seq0	NA	1	EDL17_ARATH^EDL17_ARATH^Q:114-1505,H:10-467^48.08%ID^E:1e-128^RecName: Full=Sugar transporter ERD6-like 17
YL1_CGCTCATT-TATAGCCT_comp6895_seq1	NA	1	EAAC_ARATH^EAAC_ARATH^Q:83-1255,H:1-379^69.57%ID^E:1e-172^RecName: Full=Probable envelope ADP,ATP carrier protein, chloroplastic
AR2_GAGATTCC-GGCTCTGA_comp2954_seq0	NA	1	C71DC_CATRO^C71DC_CATRO^Q:267-1745,H:2-492^50.2%ID^E:1e-166^RecName: Full=Tabersonine 16-hydroxylase
YL1_CGCTCATT-TATAGCCT_comp3031_seq4	NA	1	.
YL3_CGCTCATT-ATAGAGGC_comp22491_seq0	NA	1	.
YL1_CGCTCATT-TATAGCCT_comp15661_seq0	NA	1	.
YS1_GAGATTCC-TATAGCCT_comp3802_seq2	NA	1	.
YL1_CGCTCATT-TATAGCCT_comp5672_seq0	NA	1	BH048_ARATH^BH048_ARATH^Q:632-1372,H:27-270^50.2%ID^E:3e-51^RecName: Full=Transcription factor bHLH48
YL1_CGCTCATT-TATAGCCT_comp3021_seq0	NA	1	.
YS2_GAGATTCC-ATAGAGGC_comp776_seq0	PAS	1	THCAS_CANSA^THCAS_CANSA^Q:197-1681,H:32-540^44.92%ID^E:1e-133^RecName: Full=Tetrahydrocannabinolic acid synthase
YS2_GAGATTCC-ATAGAGGC_comp1760_seq0	CrTPT2	1	AB32G_ARATH^AB32G_ARATH^Q:318-3440,H:1-1043^75.84%ID^E:0^RecName: Full=ABC transporter G family member 32 {ECO:0000303|PubMed:18299247}
YL3_CGCTCATT-ATAGAGGC_comp16614_seq0	NA	1	.
OL1_CGCTCATT-CCTATCCT_comp3680_seq3	NA	1	GLGL2_SOLTU^GLGL2_SOLTU^Q:3403-5007,H:1-519^73.83%ID^E:0^RecName: Full=Glucose-1-phosphate adenylyltransferase large subunit 2, chloroplastic/amyloplastic
YL1_CGCTCATT-TATAGCCT_comp12228_seq3	NA	1	.
YS2_GAGATTCC-ATAGAGGC_comp16202_seq0	NA	1	.
YL1_CGCTCATT-TATAGCCT_comp2644_seq3	NA	1	GPDH2_ORYSJ^GPDH2_ORYSJ^Q:731-2029,H:16-382^68.13%ID^E:0^RecName: Full=Probable glycerol-3-phosphate dehydrogenase [NAD(+)] 2, cytosolic
YS2_GAGATTCC-ATAGAGGC_comp8501_seq0	NA	1	.
YL1_CGCTCATT-TATAGCCT_comp33140_seq0	NA	1	AAE13_ARATH^AAE13_ARATH^Q:2119-3087,H:222-540^73.37%ID^E:2e-159^RecName: Full=Malonate--CoA ligase
OL1_CGCTCATT-CCTATCCT_comp517_seq13	NA	1	ECR_ARATH^ECR_ARATH^Q:4949-5878,H:1-310^81.29%ID^E:7e-174^.^.
AR2_GAGATTCC-GGCTCTGA_comp2244_seq1	STR	1	STSY_RAUSE^STSY_RAUSE^Q:58-1059,H:1-333^77.61%ID^E:3e-171^RecName: Full=Strictosidine synthase
YL1_CGCTCATT-TATAGCCT_comp2609_seq5	NA	1	.
OL1_CGCTCATT-CCTATCCT_comp3318_seq1	NA	1	CER1_ARATH^CER1_ARATH^Q:892-2229,H:171-620^59.87%ID^E:3e-159^RecName: Full=Protein ECERIFERUM 1
YL3_CGCTCATT-ATAGAGGC_comp6907_seq0	NA	1	Y1491_ARATH^Y1491_ARATH^Q:219-1655,H:21-499^78.29%ID^E:0^RecName: Full=Uncharacterized protein At1g04910
YS1_GAGATTCC-TATAGCCT_comp65_seq1	NA	1	MER_ERYCB^MER_ERYCB^Q:109-663,H:4-194^56.25%ID^E:7e-61^RecName: Full=Methylecgonone reductase
YL3_CGCTCATT-ATAGAGGC_comp3908_seq3	NA	1	CSE_ARATH^CSE_ARATH^Q:258-1172,H:23-323^31.7%ID^E:1e-41^RecName: Full=Caffeoylshikimate esterase
YL3_CGCTCATT-ATAGAGGC_comp1258_seq0	SAT	1	VINSY_RAUSE^VINSY_RAUSE^Q:163-1392,H:1-408^65.06%ID^E:0^RecName: Full=Vinorine synthase
YS2_GAGATTCC-ATAGAGGC_comp32416_seq0	NA	1	CADH6_ORYSJ^CADH6_ORYSJ^Q:13-246,H:70-147^75.64%ID^E:5e-34^RecName: Full=Probable cinnamyl alcohol dehydrogenase 6
YL3_CGCTCATT-ATAGAGGC_comp13569_seq0	NA	1	.
YS2_GAGATTCC-ATAGAGGC_comp3900_seq3	NA	1	FH3_ORYSJ^FH3_ORYSJ^Q:2023-3180,H:809-1205^77.58%ID^E:0^RecName: Full=Formin-like protein 3
YL1_CGCTCATT-TATAGCCT_comp193_seq6	NA	1	RL362_ARATH^RL362_ARATH^Q:123-452,H:1-112^80.36%ID^E:9e-49^RecName: Full=60S ribosomal protein L36-2

TableS2

YS2_GAGATTCC-ATAGAGGC_comp2915_seq1	CMS	1	ISPD_ARATH^ISPD_ARATH^Q:1565-2311,H:56-299^77.51%ID^E:1e-121^RecName: Full=2-C-methyl-D-erythritol 4-phosphate cytidylyltransferase, chloroplastic
YS2_GAGATTCC-ATAGAGGC_comp5426_seq0	NA	1	TRY_ARATH^TRY_ARATH^Q:78-326,H:7-87^68.67%ID^E:1e-28^RecName: Full=Transcription factor TRY
YL1_CGCTCATT-TATAGCCT_comp6500_seq0	NA	1	SHN2_ARATH^SHN2_ARATH^Q:172-756,H:1-189^57.36%ID^E:2e-48^RecName: Full=Ethylene-responsive transcription factor SHINE 2
YL1_CGCTCATT-TATAGCCT_comp621_seq0	NA	1	.
YS1_GAGATTCC-TATAGCCT_comp445_seq2	DXR	1	DXR_MENPI^DXR_MENPI^Q:367-1776,H:1-470^86.86%ID^E:0^RecName: Full=1-deoxy-D-xylulose 5-phosphate reductoisomerase, chloroplastic
YS1_GAGATTCC-TATAGCCT_comp24972_seq0	SLS1	1	C72A1_CATRO^C72A1_CATRO^Q:1427-2701,H:93-517^91.29%ID^E:0^RecName: Full=Secologanin synthase
YL1_CGCTCATT-TATAGCCT_comp14134_seq1	NA	1	.
YL1_CGCTCATT-TATAGCCT_comp15133_seq0	NA	1	GDL17_ARATH^GDL17_ARATH^Q:182-1192,H:34-370^70.21%ID^E:4e-171^RecName: Full=GDSL esterase/lipase At1g33811
YL1_CGCTCATT-TATAGCCT_comp2275_seq4	NA	1	UBP1_NICPL^UBP1_NICPL^Q:1100-2194,H:21-384^86.89%ID^E:0^RecName: Full=Oligouridylate-binding protein 1
YS2_GAGATTCC-ATAGAGGC_comp2920_seq0	NA	1	PPR39_ARATH^PPR39_ARATH^Q:245-658,H:69-204^39.13%ID^E:1e-35^RecName: Full=Pentatricopeptide repeat-containing protein At1g12775, mitochondrial
YS2_GAGATTCC-ATAGAGGC_comp10625_seq1	NA	1	Y2921_ARATH^Y2921_ARATH^Q:31-1602,H:4-502^32%ID^E:9e-60^RecName: Full=Putative leucine-rich repeat receptor-like protein kinase At2g19210
YL1_CGCTCATT-TATAGCCT_comp29020_seq2	NA	1	.
YS1_GAGATTCC-TATAGCCT_comp22171_seq0	NA	1	C81F1_ARATH^C81F1_ARATH^Q:852-1457,H:300-499^50%ID^E:1e-43^RecName: Full=Cytochrome P450 81F1 {ECO:0000305}
YL1_CGCTCATT-TATAGCCT_comp4005_seq1	NA	1	WSD1_ARATH^WSD1_ARATH^Q:319-1278,H:61-386^27.03%ID^E:1e-26^RecName: Full=O-acyltransferase WSD1
YS2_GAGATTCC-ATAGAGGC_comp3651_seq3	NA	1	FHYRK_ARATH^FHYRK_ARATH^Q:1731-2336,H:178-379^81.19%ID^E:6e-110^RecName: Full=Bifunctional riboflavin kinase/FMN phosphatase
YS2_GAGATTCC-ATAGAGGC_comp3692_seq0	NA	1	SCRK6_ARATH^SCRK6_ARATH^Q:420-1502,H:28-384^81.16%ID^E:1e-177^RecName: Full=Probable fructokinase-6, chloroplastic
YS1_GAGATTCC-TATAGCCT_comp18331_seq0	NA	1	.
YL1_CGCTCATT-TATAGCCT_comp1075_seq1	NA	1	.
YL1_CGCTCATT-TATAGCCT_comp444_seq0	DL7H	1	C72A1_CATRO^C72A1_CATRO^Q:232-1896,H:1-522^51.17%ID^E:0^RecName: Full=Secologanin synthase
YL3_CGCTCATT-ATAGAGGC_comp9604_seq2	NA	1	KGLT_PETHY^KGLT_PETHY^Q:231-1583,H:5-448^64.46%ID^E:0^RecName: Full=Kaempferol 3-O-beta-D-galactosyltransferase
YL3_CGCTCATT-ATAGAGGC_comp1778_seq0	NA	1	BGAL5_ARATH^BGAL5_ARATH^Q:538-2550,H:61-731^71.83%ID^E:0^RecName: Full=Beta-galactosidase 5
YL3_CGCTCATT-ATAGAGGC_comp412_seq0	NA	1	RNFT2_MOUSE^RNFT2_MOUSE^Q:1847-2782,H:129-439^28.25%ID^E:1e-33^RecName: Full=RING finger and transmembrane domain-containing protein 2
YS2_GAGATTCC-ATAGAGGC_comp10747_seq0	NA	1	.
YL3_CGCTCATT-ATAGAGGC_comp10883_seq2	NA	1	DOF53_ARATH^DOF53_ARATH^Q:332-622,H:59-168^58.18%ID^E:6e-30^RecName: Full=Dof zinc finger protein DOF5.3
YL3_CGCTCATT-ATAGAGGC_comp22283_seq0	NA	1	SMT1_ARATH^SMT1_ARATH^Q:458-1018,H:149-335^78.07%ID^E:2e-106^RecName: Full=Cycloartenol-C-24-methyltransferase
OL1_CGCTCATT-CCTATCCT_comp35745_seq0	NA	1	.
YS2_GAGATTCC-ATAGAGGC_comp1818_seq4	NA	1	IDD9_ARATH^IDD9_ARATH^Q:401-1399,H:39-365^59%ID^E:2e-95^RecName: Full=Protein indeterminate-domain 9 {ECO:0000303|PubMed:16784536}
YL1_CGCTCATT-TATAGCCT_comp2414_seq3	NA	2	CCD53_ORYSJ^CCD53_ORYSJ^Q:2149-2952,H:59-328^33.09%ID^E:8e-34^RecName: Full=Cyclin-D5-3
YL3_CGCTCATT-ATAGAGGC_comp25903_seq0	NA	2	.
YL1_CGCTCATT-TATAGCCT_comp5995_seq1	NA	2	RH52A_ORYSJ^RH52A_ORYSJ^Q:509-1651,H:80-474^47.73%ID^E:1e-100^RecName: Full=DEAD-box ATP-dependent RNA helicase 52A
YS1_GAGATTCC-TATAGCCT_comp17005_seq0	NA	2	PID_ARATH^PID_ARATH^Q:220-1452,H:37-438^63.55%ID^E:3e-177^RecName: Full=Protein kinase PINOID
YS2_GAGATTCC-ATAGAGGC_comp4578_seq8	NA	2	DPOLA_ORYSJ^DPOLA_ORYSJ^Q:1194-2783,H:295-831^67.04%ID^E:0^RecName: Full=DNA polymerase alpha catalytic subunit
YS2_GAGATTCC-ATAGAGGC_comp7927_seq0	NA	2	.
YL3_CGCTCATT-ATAGAGGC_comp7058_seq2	NA	2	Y2182_ARATH^Y2182_ARATH^Q:931-3513,H:27-888^73.15%ID^E:0^.^.
YS1_GAGATTCC-TATAGCCT_comp204_seq5	NA	2	ABP20_PRUPE^ABP20_PRUPE^Q:2853-3449,H:4-202^66%ID^E:6e-77^RecName: Full=Auxin-binding protein ABP20
YS2_GAGATTCC-ATAGAGGC_comp11930_seq0	NA	2	H1_MAIZE^H1_MAIZE^Q:289-492,H:52-119^64.71%ID^E:5e-21^RecName: Full=Histone H1
YL3_CGCTCATT-ATAGAGGC_comp12994_seq0	NA	2	BGAL8_ARATH^BGAL8_ARATH^Q:171-2483,H:37-851^54.45%ID^E:0^RecName: Full=Beta-galactosidase 8
YS1_GAGATTCC-TATAGCCT_comp13633_seq0	NA	2	INVB_ARATH^INVB_ARATH^Q:188-1858,H:13-570^78.25%ID^E:0^RecName: Full=Probable alkaline/neutral invertase B {ECO:0000305}
YS1_GAGATTCC-TATAGCCT_comp1212_seq3	NA	2	GRF6_ORYSJ^GRF6_ORYSJ^Q:1117-1470,H:157-271^70.34%ID^E:1e-45^RecName: Full=Growth-regulating factor 6
YS1_GAGATTCC-TATAGCCT_comp767_seq6	NA	2	.
YL3_CGCTCATT-ATAGAGGC_comp3939_seq2	NA	2	Y4374_ARATH^Y4374_ARATH^Q:908-2677,H:29-622^61.27%ID^E:0^RecName: Full=Probable inactive receptor kinase At4g23740
OL3_CGCTCATT-GGCTCTGA_comp6787_seq2	redox1	2	NU155_ARATH^NU155_ARATH^Q:1019-5659,H:1-1464^65.74%ID^E:0^RecName: Full=Nuclear pore complex protein NUP155 {ECO:0000303|PubMed:12034489}
YS1_GAGATTCC-TATAGCCT_comp10834_seq2	NA	2	XYNF1_ASPOR^XYNF1_ASPOR^Q:1037-1792,H:42-293^29.77%ID^E:2e-28^RecName: Full=Endo-1,4-beta-xylanase F1
YS1_GAGATTCC-TATAGCCT_comp124_seq0	NA	2	LAL5_ARATH^LAL5_ARATH^Q:2436-3797,H:11-464^60.79%ID^E:8e-167^.^.

TableS2

YL1_CGCTCATT-TATAGCCT_comp14048_seq1	NA	2	DPB_ARATH^DPB_ARATH^Q:259-624,H:104-226^53.23%ID^E:2e-32^RecName: Full=Transcription factor-like protein DPB
YS2_GAGATTCC-ATAGAGGC_comp48_seq0	NA	2	CIPKC_ARATH^CIPKC_ARATH^Q:329-1636,H:20-459^78.64%ID^E:0^RecName: Full=CBL-interacting serine/threonine-protein kinase 12
YL3_CGCTCATT-ATAGAGGC_comp662_seq3	NA	2	CADH6_ORYSJ^CADH6_ORYSJ^Q:95-1162,H:2-358^72.83%ID^E:1e-177^RecName: Full=Probable cinnamyl alcohol dehydrogenase 6
YS2_GAGATTCC-ATAGAGGC_comp369_seq0	NA	2	RL74_ARATH^RL74_ARATH^Q:286-1023,H:1-244^79.27%ID^E:2e-130^RecName: Full=60S ribosomal protein L7-4
YS2_GAGATTCC-ATAGAGGC_comp705_seq0	NA	2	21KD_DAUCA^21KD_DAUCA^Q:736-1218,H:28-188^55.28%ID^E:1e-58^RecName: Full=21 kDa protein
YS1_GAGATTCC-TATAGCCT_comp11479_seq0	NA	2	CMT2_ARATH^CMT2_ARATH^Q:127-813,H:726-950^53.68%ID^E:7e-116^RecName: Full=DNA (cytosine-5)-methyltransferase CMT2
YL1_CGCTCATT-TATAGCCT_comp8260_seq1	NA	2	GTOMC_ORYSJ^GTOMC_ORYSJ^Q:1-264,H:136-223^55.68%ID^E:4e-24^RecName: Full=Probable tocopherol O-methyltransferase, chloroplastic
YS1_GAGATTCC-TATAGCCT_comp17_seq1	NA	2	TET3_ARATH^TET3_ARATH^Q:1322-2176,H:1-285^68.42%ID^E:5e-125^RecName: Full=Tetraspanin-3
YS2_GAGATTCC-ATAGAGGC_comp2803_seq0	NA	2	Y3228_ARATH^Y3228_ARATH^Q:1273-1671,H:13-147^52.59%ID^E:3e-39^RecName: Full=PHD finger protein At3g20280
YL1_CGCTCATT-TATAGCCT_comp616_seq1	NA	2	CADH6_ORYSJ^CADH6_ORYSJ^Q:536-1603,H:2-358^72.83%ID^E:0^RecName: Full=Probable cinnamyl alcohol dehydrogenase 6
YL1_CGCTCATT-TATAGCCT_comp11114_seq0	NA	2	IAA17_ARATH^IAA17_ARATH^Q:84-659,H:2-219^46.33%ID^E:3e-55^RecName: Full=Auxin-responsive protein IAA17
AR2_GAGATTCC-GGCTCTGA_comp969_seq0	NA	2	H4_SOYBN^H4_SOYBN^Q:220-453,H:22-99^100%ID^E:5e-45^RecName: Full=Histone H4
YL1_CGCTCATT-TATAGCCT_comp3917_seq0	NA	2	Y5720_ARATH^Y5720_ARATH^Q:381-2264,H:30-666^52.15%ID^E:0^RecName: Full=Probable inactive receptor kinase At5g67200
YL1_CGCTCATT-TATAGCCT_comp3285_seq0	NA	2	VIL2_ARATH^VIL2_ARATH^Q:460-2259,H:2-714^36.29%ID^E:1e-94^RecName: Full=VIN3-like protein 2
YL3_CGCTCATT-ATAGAGGC_comp15965_seq1	NA	2	.
YL1_CGCTCATT-TATAGCCT_comp302_seq2	NMT	2	GTOMC_ORYSJ^GTOMC_ORYSJ^Q:1698-2549,H:75-357^48.24%ID^E:3e-88^RecName: Full=Probable tocopherol O-methyltransferase, chloroplastic
YS2_GAGATTCC-ATAGAGGC_comp12704_seq0	NA	2	NAT1_ARATH^NAT1_ARATH^Q:327-1883,H:2-520^86.71%ID^E:0^RecName: Full=Nucleobase-ascorbate transporter 1
YS2_GAGATTCC-ATAGAGGC_comp61_seq0	Vm16OMT	2	HTOMT_CATRO^HTOMT_CATRO^Q:2794-3867,H:1-355^70.28%ID^E:8e-156^RecName: Full=Tabersonine 16-O-methyltransferase
YS1_GAGATTCC-TATAGCCT_comp8503_seq3	NA	2	ROGF1_ARATH^ROGF1_ARATH^Q:764-2506,H:1-548^66.55%ID^E:0^RecName: Full=Rop guanine nucleotide exchange factor 1
YL3_CGCTCATT-ATAGAGGC_comp9464_seq1	NA	2	.
YS2_GAGATTCC-ATAGAGGC_comp5812_seq2	NA	2	LTL1_ARATH^LTL1_ARATH^Q:1690-1920,H:289-365^76.62%ID^E:8e-35^RecName: Full=GDSL esterase/lipase LTL1
YS2_GAGATTCC-ATAGAGGC_comp21866_seq0	NA	2	.
AR1_GAGATTCC-CCTATCCT_comp3145_seq1	VmPiNMT	2	GTOMC_ORYSJ^GTOMC_ORYSJ^Q:2-916,H:92-359^41.23%ID^E:4e-80^RecName: Full=Probable tocopherol O-methyltransferase, chloroplastic
AR2_GAGATTCC-GGCTCTGA_comp2837_seq1	NA	2	PPO1_IPOBA^PPO1_IPOBA^Q:341-1708,H:1-457^63.83%ID^E:0^RecName: Full=Polyphenol oxidase I, chloroplastic
YS2_GAGATTCC-ATAGAGGC_comp8982_seq1	NA	2	.
YL3_CGCTCATT-ATAGAGGC_comp2768_seq1	NA	2	.
AR1_GAGATTCC-CCTATCCT_comp1782_seq4	NA	2	KC1D_PONAB^KC1D_PONAB^Q:1149-2012,H:3-278^38.51%ID^E:6e-55^RecName: Full=Casein kinase I isoform delta
YS1_GAGATTCC-TATAGCCT_comp10782_seq1	NA	2	SMC5_ARATH^SMC5_ARATH^Q:281-3400,H:3-1042^62.21%ID^E:0^RecName: Full=Structural maintenance of chromosomes protein 5
YL1_CGCTCATT-TATAGCCT_comp17245_seq0	NA	2	.
YS2_GAGATTCC-ATAGAGGC_comp324_seq0	TDC	2	DDC_CATRO^DDC_CATRO^Q:661-2163,H:1-500^88.45%ID^E:0^RecName: Full=Aromatic-L-amino-acid decarboxylase
YL3_CGCTCATT-ATAGAGGC_comp11681_seq0	NA	2	ANXD3_ARATH^ANXD3_ARATH^Q:22-975,H:1-321^57.63%ID^E:1e-129^RecName: Full=Annexin D3
AR1_GAGATTCC-CCTATCCT_comp386_seq2	NA	2	MLO6_ARATH^MLO6_ARATH^Q:748-2376,H:5-581^59.66%ID^E:0^RecName: Full=MLO-like protein 6
YL1_CGCTCATT-TATAGCCT_comp12249_seq2	NA	2	WAXS4_ARATH^WAXS4_ARATH^Q:215-1186,H:21-337^39.2%ID^E:6e-60^RecName: Full=Probable long-chain-alcohol O-fatty-acyltransferase 4
OL1_CGCTCATT-CCTATCCT_comp6039_seq0	NA	2	.
YL1_CGCTCATT-TATAGCCT_comp202_seq1	NA	3	RUBA_PEA^RUBA_PEA^Q:1424-3199,H:1-587^86.99%ID^E:0^RecName: Full=RuBisCO large subunit-binding protein subunit alpha, chloroplastic
YS2_GAGATTCC-ATAGAGGC_comp28794_seq0	DXS3	3	DXS2_ORYSJ^DXS2_ORYSJ^Q:101-811,H:12-247^75.21%ID^E:2e-115^RecName: Full=Probable 1-deoxy-D-xylulose-5-phosphate synthase 2, chloroplastic
YL3_CGCTCATT-ATAGAGGC_comp22045_seq0	NA	3	.
YS2_GAGATTCC-ATAGAGGC_comp2791_seq1	NA	3	FLS2_ARATH^FLS2_ARATH^Q:110-1351,H:8-477^28.54%ID^E:2e-29^RecName: Full=LRR receptor-like serine/threonine-protein kinase FLS2
YL3_CGCTCATT-ATAGAGGC_comp3100_seq5	NA	3	PII2_ARATH^PII2_ARATH^Q:2363-3541,H:30-423^61.06%ID^E:2e-132^RecName: Full=Piriformospora indica-insensitive protein 2
AR1_GAGATTCC-CCTATCCT_comp25230_seq0	NA	3	.
YL3_CGCTCATT-ATAGAGGC_comp2756_seq3	NA	3	CELF3_BOVIN^CELF3_BOVIN^Q:830-2026,H:5-439^36.61%ID^E:1e-68^RecName: Full=CUGBP Elav-like family member 3
YL3_CGCTCATT-ATAGAGGC_comp477_seq1	IS	3	IRIS_CATRO^IRIS_CATRO^Q:199-1458,H:1-388^83.33%ID^E:0^RecName: Full=Iridoid synthase
YS2_GAGATTCC-ATAGAGGC_comp37355_seq0	NA	3	RAC1_BETVU^RAC1_BETVU^Q:900-1328,H:54-197^92.36%ID^E:5e-75^RecName: Full=Rac-like GTP-binding protein RHO1
YL3_CGCTCATT-ATAGAGGC_comp10649_seq0	NA	3	ARAK_ARATH^ARAK_ARATH^Q:617-3511,H:66-1034^72.38%ID^E:0^RecName: Full=L-arabinokinase

TableS2

YS2_GAGATTCC-ATAGAGGC_comp2711_seq8	NA	3	LVSG_DICDI^LVSG_DICDI^Q:1856-2908,H:384-772^38.39%ID^E:4e-71^RecName: Full=Probable inactive serine/threonine-protein kinase lvsG
YL1_CGCTCATT-TATAGCCT_comp5166_seq0	NA	3	.
YL3_CGCTCATT-ATAGAGGC_comp3314_seq1	NA	3	Y3565_ARATH^Y3565_ARATH^Q:1642-2610,H:164-498^61.83%ID^E:1e-129^RecName: Full=Probable inactive receptor-like protein kinase At3g56050
YS1_GAGATTCC-TATAGCCT_comp1665_seq2	NA	3	BLH6_ARATH^BLH6_ARATH^Q:1000-2730,H:3-494^42.76%ID^E:3e-115^RecName: Full=BEL1-like homeodomain protein 6
YL3_CGCTCATT-ATAGAGGC_comp7757_seq0	NA	3	Y5656_ARATH^Y5656_ARATH^Q:312-2372,H:1-667^56.05%ID^E:0^RecName: Full=BTB/POZ domain-containing protein At5g66560
YL1_CGCTCATT-TATAGCCT_comp9867_seq0	NA	3	CIPK1_ORYSJ^CIPK1_ORYSJ^Q:1-276,H:148-239^76.09%ID^E:7e-75^RecName: Full=CBL-interacting protein kinase 1
AR1_GAGATTCC-CCTATCCT_comp22848_seq1	NA	3	MSI4_ARATH^MSI4_ARATH^Q:65-220,H:66-117^82.69%ID^E:3e-23^RecName: Full=WD-40 repeat-containing protein MSI4
YL1_CGCTCATT-TATAGCCT_comp7173_seq1	NA	3	RABA3_ARATH^RABA3_ARATH^Q:189-842,H:23-237^71.3%ID^E:5e-103^RecName: Full=Ras-related protein RABA3
YL1_CGCTCATT-TATAGCCT_comp6388_seq3	NA	3	PMA4_NICPL^PMA4_NICPL^Q:4708-3239,H:390-801^71.02%ID^E:0^RecName: Full=Plasma membrane ATPase 4
YL1_CGCTCATT-TATAGCCT_comp643_seq0	NA	3	RAN3_ARATH^RAN3_ARATH^Q:79-717,H:1-213^98.12%ID^E:7e-142^RecName: Full=GTP-binding nuclear protein Ran-3
YS1_GAGATTCC-TATAGCCT_comp264_seq2	NA	3	.
AR1_GAGATTCC-CCTATCCT_comp1758_seq2	NA	3	SYY_MIMIV^SYY_MIMIV^Q:868-1755,H:57-342^44.15%ID^E:1e-72^RecName: Full=Tyrosine--tRNA ligase
YS2_GAGATTCC-ATAGAGGC_comp6671_seq1	NA	3	NLTL2_ARATH^NLTL2_ARATH^Q:303-716,H:26-162^53.62%ID^E:2e-40^RecName: Full=Non-specific lipid-transfer protein-like protein At2g13820
YS1_GAGATTCC-TATAGCCT_comp14492_seq0	NA	3	RL15_PETHY^RL15_PETHY^Q:1380-1991,H:1-204^94.61%ID^E:2e-112^RecName: Full=60S ribosomal protein L15
AR2_GAGATTCC-GGCTCTGA_comp9401_seq0	NA	3	.
YL3_CGCTCATT-ATAGAGGC_comp4742_seq3	BIS1	3	BBR_ARATH^BBR_ARATH^Q:2045-2518,H:180-335^67.09%ID^E:3e-53^RecName: Full=E3 ubiquitin ligase BIG BROTHER-related
YS2_GAGATTCC-ATAGAGGC_comp5910_seq2	NA	3	VSR1_ARATH^VSR1_ARATH^Q:2339-4132,H:22-623^77.24%ID^E:0^RecName: Full=Vacuolar-sorting receptor 1
YL1_CGCTCATT-TATAGCCT_comp5093_seq2	NA	3	IAA14_ARATH^IAA14_ARATH^Q:352-843,H:4-151^48.8%ID^E:7e-32^RecName: Full=Auxin-responsive protein IAA14
AR1_GAGATTCC-CCTATCCT_comp589_seq7	G10H	3	C76B6_CATRO^C76B6_CATRO^Q:1306-2784,H:1-493^86%ID^E:0^RecName: Full=Geraniol 8-hydroxylase
AR1_GAGATTCC-CCTATCCT_comp20069_seq0	NA	3	CB4_SPIOL^CB4_SPIOL^Q:1590-1081,H:80-249^90%ID^E:1e-94^RecName: Full=Chlorophyll a-b binding protein CP24, chloroplastic
YS2_GAGATTCC-ATAGAGGC_comp10055_seq1	NA	3	.
OL3_CGCTCATT-GGCTCTGA_comp7833_seq1	NA	3	POL4_DROME^POL4_DROME^Q:611-1714,H:876-1234^25.88%ID^E:6e-29^RecName: Full=Retrovirus-related Pol polyprotein from transposon 412
YS2_GAGATTCC-ATAGAGGC_comp558_seq3	DL7GT	3	UGT8_CATRO^UGT8_CATRO^Q:834-2273,H:3-482^88.12%ID^E:0^RecName: Full=7-deoxyloganetic acid glucosyltransferase
YL1_CGCTCATT-TATAGCCT_comp20383_seq0	NA	3	.
YL1_CGCTCATT-TATAGCCT_comp5868_seq1	NA	3	VAP21_ARATH^VAP21_ARATH^Q:498-1160,H:1-220^64.29%ID^E:2e-81^RecName: Full=Vesicle-associated protein 2-1
YL1_CGCTCATT-TATAGCCT_comp7706_seq0	NA	3	.
YS1_GAGATTCC-TATAGCCT_comp5831_seq1	NA	3	.
YS1_GAGATTCC-TATAGCCT_comp10376_seq0	NA	3	.
AR1_GAGATTCC-CCTATCCT_comp4243_seq0	NA	3	.
YS2_GAGATTCC-ATAGAGGC_comp1901_seq0	NA	3	ASPG1_ARATH^ASPG1_ARATH^Q:217-1569,H:45-500^58.42%ID^E:2e-169^RecName: Full=Protein ASPARTIC PROTEASE IN GUARD CELL 1
AR1_GAGATTCC-CCTATCCT_comp3050_seq0	THAS1	4	10HGO_CATRO^10HGO_CATRO^Q:256-1335,H:1-360^86.94%ID^E:0^RecName: Full=8-hydroxygeraniol dehydrogenase
OL1_CGCTCATT-CCTATCCT_comp1163_seq6	NA	4	GATP3_SOLLC^GATP3_SOLLC^Q:1986-2429,H:370-516^80.41%ID^E:6e-74^RecName: Full=Gamma aminobutyrate transaminase 3, chloroplastic
AR1_GAGATTCC-CCTATCCT_comp8371_seq0	NA	4	HIS2_ARATH^HIS2_ARATH^Q:186-1004,H:23-279^68.5%ID^E:9e-121^RecName: Full=Histidine biosynthesis bifunctional protein hisIE, chloroplastic
AR1_GAGATTCC-CCTATCCT_comp28_seq3	NA	4	.
YL3_CGCTCATT-ATAGAGGC_comp3627_seq0	NA	4	CYC6_ARATH^CYC6_ARATH^Q:474-698,H:100-174^88%ID^E:7e-45^RecName: Full=Cytochrome c6, chloroplastic {ECO:0000303|PubMed:12037572}
YS1_GAGATTCC-TATAGCCT_comp8635_seq3	NA	4	.
YS2_GAGATTCC-ATAGAGGC_comp8318_seq1	T3O	4	C71D7_SOLCH^C71D7_SOLCH^Q:3390-3109,H:82-182^46.53%ID^E:2e-49^RecName: Full=Cytochrome P450 71D7
YS1_GAGATTCC-TATAGCCT_comp6764_seq2	V19H	4	HACL_ARATH^HACL_ARATH^Q:229-1950,H:1-572^79.62%ID^E:0^RecName: Full=2-hydroxyacyl-CoA lyase
YL3_CGCTCATT-ATAGAGGC_comp14054_seq2	NA	4	.
YS1_GAGATTCC-TATAGCCT_comp11712_seq2	NA	4	.
AR1_GAGATTCC-CCTATCCT_comp2271_seq1	NA	4	.
YL3_CGCTCATT-ATAGAGGC_comp70_seq0	8HGO	4	ADHX_ORYSI^ADHX_ORYSI^Q:149-1249,H:8-377^44.89%ID^E:1e-92^RecName: Full=Alcohol dehydrogenase class-3
YL3_CGCTCATT-ATAGAGGC_comp4424_seq2	NA	4	RGLG2_ARATH^RGLG2_ARATH^Q:675-1517,H:184-468^71.23%ID^E:5e-146^RecName: Full=E3 ubiquitin-protein ligase RGLG2
YS2_GAGATTCC-ATAGAGGC_comp6211_seq1	NA	4	KPRS4_SPIOL^KPRS4_SPIOL^Q:220-1173,H:1-318^87.74%ID^E:0^RecName: Full=Ribose-phosphate pyrophosphokinase 4

YL3_CGCTCATT-ATAGAGGC_comp25807_seq0	NA	4	.
YS2_GAGATTCC-ATAGAGGC_comp1127_seq1	NA	4	STIP1_ARATH^STIP1_ARATH^Q:1314-3857,H:1-841^53.51%ID^E:0^RecName: Full=Septin and tuftelin-interacting protein 1 homolog 1
YS1_GAGATTCC-TATAGCCT_comp72982_seq0	NA	4	.
YL1_CGCTCATT-TATAGCCT_comp13722_seq1	NA	4	PDPK2_ARATH^PDPK2_ARATH^Q:848-1456,H:32-234^91.13%ID^E:0^RecName: Full=3-phosphoinositide-dependent protein kinase 2
YS2_GAGATTCC-ATAGAGGC_comp13836_seq1	NA	4	MCM9_ARATH^MCM9_ARATH^Q:917-2809,H:12-640^75.63%ID^E:0^RecName: Full=Probable DNA helicase MCM9
YL1_CGCTCATT-TATAGCCT_comp1306_seq0	NA	4	THO4A_ARATH^THO4A_ARATH^Q:205-741,H:1-172^59.67%ID^E:5e-44^RecName: Full=THO complex subunit 4A
OL3_CGCTCATT-GGCTCTGA_comp9667_seq1	NA	4	CBSX3_ARATH^CBSX3_ARATH^Q:421-885,H:16-206^51.31%ID^E:1e-51^RecName: Full=CBS domain-containing protein CBSX3, mitochondrial
YL3_CGCTCATT-ATAGAGGC_comp14103_seq1	NA	4	.
AR1_GAGATTCC-CCTATCCT_comp4362_seq3	NA	4	RP8L2_ARATH^RP8L2_ARATH^Q:996-2741,H:345-901^31.18%ID^E:1e-68^RecName: Full=Probable disease resistance RPP8-like protein 2
OL1_CGCTCATT-CCTATCCT_comp5736_seq1	NA	5	2A5B_ARATH^2A5B_ARATH^Q:711-2147,H:1-485^70.43%ID^E:0^RecName: Full=Serine/threonine protein phosphatase 2A 57 kDa regulatory subunit B' beta isoform
OL3_CGCTCATT-GGCTCTGA_comp87_seq0	NA	5	GLNA2_VITVI^GLNA2_VITVI^Q:1-246,H:225-306^95.12%ID^E:2e-44^RecName: Full=Glutamine synthetase cytosolic isozyme 2
OL3_CGCTCATT-GGCTCTGA_comp166_seq3	NA	5	.
OL3_CGCTCATT-GGCTCTGA_comp8049_seq3	NA	5	.
OL3_CGCTCATT-GGCTCTGA_comp5964_seq3	NA	5	.
OL3_CGCTCATT-GGCTCTGA_comp3166_seq0	NA	5	C71DC_CATRO^C71DC_CATRO^Q:276-1646,H:38-492^59.04%ID^E:0^RecName: Full=Tabersonine 16-hydroxylase
OL3_CGCTCATT-GGCTCTGA_comp6046_seq2	NA	5	.
OL3_CGCTCATT-GGCTCTGA_comp3275_seq3	NA	5	LENG8_XENLA^LENG8_XENLA^Q:1359-2120,H:515-765^47.86%ID^E:4e-66^RecName: Full=Leukocyte receptor cluster member 8 homolog
OL1_CGCTCATT-CCTATCCT_comp1318_seq1	VS	5	HIDM_GLYEC^HIDM_GLYEC^Q:72-566,H:12-178^52.07%ID^E:2e-43^RecName: Full=2-hydroxyisoflavanone dehydratase
YS1_GAGATTCC-TATAGCCT_comp1103_seq2	NA	6	.
YS1_GAGATTCC-TATAGCCT_comp6148_seq1	NA	6	IDD2_ARATH^IDD2_ARATH^Q:253-624,H:1-122^76.61%ID^E:1e-62^RecName: Full=Protein indeterminate-domain 2 {ECO:0000303|PubMed:16784536}
YL1_CGCTCATT-TATAGCCT_comp20633_seq0	NA	6	Y1174_SYNP6^Y1174_SYNP6^Q:188-1219,H:2-339^55.36%ID^E:5e-100^RecName: Full=Uncharacterized lipoprotein syc1174_c
OL3_CGCTCATT-GGCTCTGA_comp4608_seq0	NA	6	ARID5_ARATH^ARID5_ARATH^Q:1224-2120,H:131-434^66.23%ID^E:2e-121^RecName: Full=AT-rich interactive domain-containing protein 5
AR2_GAGATTCC-GGCTCTGA_comp1085_seq1	NA	6	RAB7_VIGAC^RAB7_VIGAC^Q:2089-2649,H:19-206^90.96%ID^E:5e-117^RecName: Full=Ras-related protein Rab7
YS2_GAGATTCC-ATAGAGGC_comp11931_seq1	NA	6	YNB1_YEAST^YNB1_YEAST^Q:499-1446,H:51-417^31.84%ID^E:2e-47^RecName: Full=Uncharacterized protein YNL011C
OL3_CGCTCATT-GGCTCTGA_comp5315_seq0	NA	6	TOC34_PEA^TOC34_PEA^Q:691-1266,H:111-301^65.1%ID^E:6e-79^RecName: Full=Translocase of chloroplast 34
AR1_GAGATTCC-CCTATCCT_comp2374_seq0	NA	6	CLPP6_ARATH^CLPP6_ARATH^Q:485-1204,H:38-271^77.92%ID^E:1e-120^RecName: Full=ATP-dependent Clp protease proteolytic subunit 6, chloroplastic {ECO:0000303|PubMed:11299370}
OL3_CGCTCATT-GGCTCTGA_comp8459_seq0	NA	6	ARFH_ARATH^ARFH_ARATH^Q:582-2246,H:185-773^61.59%ID^E:0^RecName: Full=Auxin response factor 8
YS2_GAGATTCC-ATAGAGGC_comp3776_seq2	NA	6	CSP1_ARATH^CSP1_ARATH^Q:331-1200,H:6-299^43.56%ID^E:9e-32^RecName: Full=Cold shock protein 1
OL1_CGCTCATT-CCTATCCT_comp4311_seq2	NA	6	.
OL3_CGCTCATT-GGCTCTGA_comp9977_seq1	ORCA2	6	ERF99_ARATH^ERF99_ARATH^Q:673-903,H:91-168^73.08%ID^E:2e-33^RecName: Full=Ethylene-responsive transcription factor 13
AR1_GAGATTCC-CCTATCCT_comp3443_seq0	NA	7	F3PH_ARATH^F3PH_ARATH^Q:174-1706,H:1-508^39.96%ID^E:4e-121^RecName: Full=Flavonoid 3'-monooxygenase
AR1_GAGATTCC-CCTATCCT_comp76_seq2	NA	7	E13B_WHEAT^E13B_WHEAT^Q:367-1611,H:26-453^45.92%ID^E:9e-123^RecName: Full=Glucan endo-1,3-beta-glucosidase
AR2_GAGATTCC-GGCTCTGA_comp7052_seq0	NA	7	ANX4_FRAAN^ANX4_FRAAN^Q:82-1017,H:1-314^64.97%ID^E:9e-146^RecName: Full=Annexin-like protein RJ4
AR2_GAGATTCC-GGCTCTGA_comp35_seq9	NA	7	C72A1_CATRO^C72A1_CATRO^Q:667-2238,H:1-524^90.84%ID^E:0^RecName: Full=Secologanin synthase
YS1_GAGATTCC-TATAGCCT_comp9360_seq0	NA	7	LHTL6_ARATH^LHTL6_ARATH^Q:336-1577,H:19-432^72.95%ID^E:0^RecName: Full=Lysine histidine transporter-like 6
AR1_GAGATTCC-CCTATCCT_comp15336_seq0	NA	7	AKT1_ARATH^AKT1_ARATH^Q:195-2792,H:7-857^67.97%ID^E:0^RecName: Full=Potassium channel AKT1
AR1_GAGATTCC-CCTATCCT_comp902_seq1	NA	7	AL3H1_ARATH^AL3H1_ARATH^Q:3199-3702,H:191-358^72.02%ID^E:2e-74^RecName: Full=Aldehyde dehydrogenase family 3 member H1
AR1_GAGATTCC-CCTATCCT_comp7406_seq5	NA	7	IDD2_ARATH^IDD2_ARATH^Q:585-2090,H:1-447^52.38%ID^E:5e-124^RecName: Full=Protein indeterminate-domain 2 {ECO:0000303|PubMed:16784536}
YL1_CGCTCATT-TATAGCCT_comp695_seq0	GES	7	GERS_OCIBA^GERS_OCIBA^Q:288-1925,H:31-566^63.39%ID^E:0^RecName: Full=Geraniol synthase, chloroplastic
YL3_CGCTCATT-ATAGAGGC_comp24426_seq0	NA	8	NCPR_CATRO^NCPR_CATRO^Q:239-880,H:501-714^91.59%ID^E:2e-138^RecName: Full=NADPH--cytochrome P450 reductase {ECO:0000255|HAMAP-Rule:MF_03212}
OL3_CGCTCATT-GGCTCTGA_comp2316_seq0	MYC2	8	SYY_PARUW^SYY_PARUW^Q:1-1062,H:70-422^52.82%ID^E:1e-120^RecName: Full=Tyrosine--tRNA ligase {ECO:0000255|HAMAP-Rule:MF_02006}
YL1_CGCTCATT-TATAGCCT_comp28191_seq0	NA	8	.
YL1_CGCTCATT-TATAGCCT_comp5601_seq1	NA	8	.
YL1_CGCTCATT-TATAGCCT_comp19800_seq0	NA	8	.

YL1_CGCTCATT-TATAGCCT_comp1884_seq0	NA	8	RETOL_ARATH^RETOL_ARATH^Q:403-1920,H:32-536^59.33%ID^E:0^RecName: Full=Reticuline oxidase-like protein
AR1_GAGATTCC-CCTATCCT_comp34936_seq0	NA	8	.
YL1_CGCTCATT-TATAGCCT_comp10398_seq1	NA	8	.
OL1_CGCTCATT-CCTATCCT_comp10937_seq0	NA	8	CIPKB_ARATH^CIPKB_ARATH^Q:302-1267,H:95-421^57.88%ID^E:2e-118^RecName: Full=CBL-interacting serine/threonine-protein kinase 11
OL3_CGCTCATT-GGCTCTGA_comp34446_seq0	NA	8	.
YS2_GAGATTCC-ATAGAGGC_comp4455_seq0	NA	9	.
YS1_GAGATTCC-TATAGCCT_comp18356_seq0	NA	9	.
YL1_CGCTCATT-TATAGCCT_comp10613_seq0	ORCA5	9	EF106_ARATH^EF106_ARATH^Q:111-797,H:1-190^38.43%ID^E:8e-26^RecName: Full=Ethylene-responsive transcription factor ERF106
YS1_GAGATTCC-TATAGCCT_comp14430_seq1	NA	9	.
YS1_GAGATTCC-TATAGCCT_comp9517_seq0	NA	9	EB1C_ARATH^EB1C_ARATH^Q:515-898,H:215-328^49.62%ID^E:2e-26^RecName: Full=Microtubule-associated protein RP/EB family member 1C
YS1_GAGATTCC-TATAGCCT_comp9328_seq0	NA	9	TBL34_ARATH^TBL34_ARATH^Q:661-1365,H:171-405^53.62%ID^E:1e-107^RecName: Full=Protein trichome birefringence-like 34
YL1_CGCTCATT-TATAGCCT_comp6908_seq3	NA	9	.
YS2_GAGATTCC-ATAGAGGC_comp6201_seq1	NA	9	.
YS2_GAGATTCC-ATAGAGGC_comp92_seq2	NA	9	MYO2_ARATH^MYO2_ARATH^Q:4807-6198,H:647-1116^53.24%ID^E:5e-150^RecName: Full=Myosin-2
YS2_GAGATTCC-ATAGAGGC_comp1198_seq1	NA	9	.
YL1_CGCTCATT-TATAGCCT_comp9896_seq0	NA	9	.
YL3_CGCTCATT-ATAGAGGC_comp2341_seq2	NA	9	MTDH_FRAAN^MTDH_FRAAN^Q:2520-3485,H:35-356^67.7%ID^E:5e-139^RecName: Full=Probable mannitol dehydrogenase
YL3_CGCTCATT-ATAGAGGC_comp166_seq2	CS	9	HIDH_SOYBN^HIDH_SOYBN^Q:302-1240,H:4-318^48.91%ID^E:3e-93^RecName: Full=2-hydroxyisoflavanone dehydratase
YL1_CGCTCATT-TATAGCCT_comp27681_seq0	NA	9	.
AR1_GAGATTCC-CCTATCCT_comp1435_seq3	NA	9	RAC5_ORYSJ^RAC5_ORYSJ^Q:234-755,H:1-174^94.25%ID^E:5e-117^RecName: Full=Rac-like GTP-binding protein 5
YL1_CGCTCATT-TATAGCCT_comp11923_seq3	NA	9	ASPM_SHEEP^ASPM_SHEEP^Q:867-3644,H:702-1662^24.78%ID^E:7e-52^RecName: Full=Abnormal spindle-like microcephaly-associated protein homolog
YL3_CGCTCATT-ATAGAGGC_comp4884_seq0	NA	9	.
YS1_GAGATTCC-TATAGCCT_comp37971_seq0	NA	9	.
YS1_GAGATTCC-TATAGCCT_comp15581_seq0	NA	9	.
YL1_CGCTCATT-TATAGCCT_comp6685_seq0	NA	9	.
YL3_CGCTCATT-ATAGAGGC_comp55552_seq0	NA	9	.
OL1_CGCTCATT-CCTATCCT_comp11695_seq0	NA	10	.
YS2_GAGATTCC-ATAGAGGC_comp1556_seq7	NA	10	CASTO_LOTJA^CASTO_LOTJA^Q:660-2075,H:90-565^75.68%ID^E:0^RecName: Full=Ion channel CASTOR
AR2_GAGATTCC-GGCTCTGA_comp1876_seq1	NA	10	ARP9_ORYSI^ARP9_ORYSI^Q:6424-8193,H:1-586^62.86%ID^E:0^RecName: Full=Actin-related protein 9
OL1_CGCTCATT-CCTATCCT_comp55598_seq0	NA	10	PP2A2_ARATH^PP2A2_ARATH^Q:425-973,H:124-306^96.72%ID^E:3e-129^RecName: Full=Serine/threonine-protein phosphatase PP2A-2 catalytic subunit
OL1_CGCTCATT-CCTATCCT_comp58112_seq0	NA	10	.
YL1_CGCTCATT-TATAGCCT_comp32410_seq0	NA	10	.
YS2_GAGATTCC-ATAGAGGC_comp14565_seq0	NA	10	.
AR1_GAGATTCC-CCTATCCT_comp331_seq0	NA	10	.
YL3_CGCTCATT-ATAGAGGC_comp13576_seq1	NA	10	C3H3_ARATH^C3H3_ARATH^Q:242-1228,H:23-338^45.72%ID^E:1e-77^RecName: Full=Zinc finger CCCH domain-containing protein 3
YS2_GAGATTCC-ATAGAGGC_comp805_seq0	NA	10	GGAP1_ARATH^GGAP1_ARATH^Q:704-1879,H:1-387^76.53%ID^E:0^RecName: Full=GDP-L-galactose phosphorylase 1
YS1_GAGATTCC-TATAGCCT_comp7766_seq6	NA	10	CMBL_HUMAN^CMBL_HUMAN^Q:655-1227,H:28-217^30.96%ID^E:1e-22^RecName: Full=Carboxymethylenebutenolidase homolog
YS2_GAGATTCC-ATAGAGGC_comp38970_seq0	NA	10	.
AR1_GAGATTCC-CCTATCCT_comp1158_seq0	IDI1	10	IDI1_CAMAC^IDI1_CAMAC^Q:315-1016,H:1-234^89.32%ID^E:4e-153^RecName: Full=Isopentenyl-diphosphate Delta-isomerase I
YL3_CGCTCATT-ATAGAGGC_comp6028_seq1	NA	10	.
YL3_CGCTCATT-ATAGAGGC_comp4093_seq1	NA	10	.
YS2_GAGATTCC-ATAGAGGC_comp38763_seq0	NA	10	.
OL1_CGCTCATT-CCTATCCT_comp3640_seq0	THAS2	10	10HGO_CATRO^10HGO_CATRO^Q:196-1302,H:4-360^58.54%ID^E:8e-134^RecName: Full=8-hydroxygeraniol dehydrogenase
YS2_GAGATTCC-ATAGAGGC_comp48268_seq0	NA	10	.

YS2_GAGATTCC-ATAGAGGC_comp2162_seq0	NA	10	.
YS1_GAGATTCC-TATAGCCT_comp57747_seq0	NA	10	CID9_ARATH^CID9_ARATH^Q:5-406,H:118-251^79.85%ID^E:3e-79^RecName: Full=Polyadenylate-binding protein-interacting protein 9
YS2_GAGATTCC-ATAGAGGC_comp39036_seq0	NA	10	.
YS2_GAGATTCC-ATAGAGGC_comp12232_seq0	NA	10	.
YL3_CGCTCATT-ATAGAGGC_comp33457_seq0	NA	10	.
AR1_GAGATTCC-CCTATCCT_comp4203_seq9	NA	10	UGPA_HORVU^UGPA_HORVU^Q:2-268,H:104-192^87.64%ID^E:1e-44^RecName: Full=UTP--glucose-1-phosphate uridylyltransferase
YS2_GAGATTCC-ATAGAGGC_comp1795_seq5	NA	10	.
YS2_GAGATTCC-ATAGAGGC_comp33887_seq0	NA	10	.
YS2_GAGATTCC-ATAGAGGC_comp5239_seq2	NA	10	.
OL1_CGCTCATT-CCTATCCT_comp2934_seq3	NA	11	P2C52_ARATH^P2C52_ARATH^Q:1685-3094,H:1-467^73.68%ID^E:0^RecName: Full=Probable protein phosphatase 2C 52
YS2_GAGATTCC-ATAGAGGC_comp1245_seq0	NA	11	GAI_SOLLC^GAI_SOLLC^Q:423-2090,H:26-588^67.47%ID^E:0^RecName: Full=DELLA protein GAI
YL1_CGCTCATT-TATAGCCT_comp2035_seq0	NA	11	SDC1_ORYSJ^SDC1_ORYSJ^Q:1806-2429,H:274-481^62.02%ID^E:4e-88^RecName: Full=Serine decarboxylase 1
YS2_GAGATTCC-ATAGAGGC_comp6216_seq0	NA	11	PAS1_ARATH^PAS1_ARATH^Q:289-1812,H:1-460^65.94%ID^E:0^RecName: Full=Peptidyl-prolyl cis-trans isomerase PASTICCINO1
YS1_GAGATTCC-TATAGCCT_comp8724_seq0	NA	11	YQK1_SCHPO^YQK1_SCHPO^Q:82-999,H:3-303^33.23%ID^E:4e-35^RecName: Full=UPF0676 protein C1494.01
YS1_GAGATTCC-TATAGCCT_comp6147_seq0	NA	11	.
YL3_CGCTCATT-ATAGAGGC_comp3510_seq4	NA	11	Y1461_ARATH^Y1461_ARATH^Q:849-2270,H:88-565^69.85%ID^E:0^RecName: Full=Probable serine/threonine-protein kinase At1g54610
YL3_CGCTCATT-ATAGAGGC_comp5404_seq1	NA	11	BP73_ORYSJ^BP73_ORYSJ^Q:359-1318,H:30-372^40.23%ID^E:1e-50^RecName: Full=SAP-like protein BP-73
YL1_CGCTCATT-TATAGCCT_comp11035_seq1	NA	11	Y1491_ARATH^Y1491_ARATH^Q:480-1850,H:44-483^35.64%ID^E:6e-73^RecName: Full=Uncharacterized protein At1g04910
YL1_CGCTCATT-TATAGCCT_comp24563_seq0	NA	11	.
OL1_CGCTCATT-CCTATCCT_comp1051_seq1	NA	11	LGUL_CICAR^LGUL_CICAR^Q:878-1276,H:54-186^80.45%ID^E:3e-73^RecName: Full=Lactoylglutathione lyase
YL1_CGCTCATT-TATAGCCT_comp9848_seq1	NA	11	.
YL3_CGCTCATT-ATAGAGGC_comp2061_seq2	NA	11	ICE1_ARATH^ICE1_ARATH^Q:966-2189,H:115-494^51.91%ID^E:5e-102^RecName: Full=Transcription factor ICE1
YL3_CGCTCATT-ATAGAGGC_comp16753_seq0	NA	11	.
YS1_GAGATTCC-TATAGCCT_comp5053_seq1	NA	11	.
YL1_CGCTCATT-TATAGCCT_comp3439_seq2	NA	11	Y5133_ARATH^Y5133_ARATH^Q:1686-3032,H:10-458^60.84%ID^E:0^RecName: Full=BTB/POZ domain-containing protein At5g41330
OL1_CGCTCATT-CCTATCCT_comp2674_seq2	NA	11	SUBL_ARATH^SUBL_ARATH^Q:289-2580,H:22-755^52.8%ID^E:0^.^.
YL1_CGCTCATT-TATAGCCT_comp12940_seq0	NA	11	CDA7L_HUMAN^CDA7L_HUMAN^Q:639-935,H:343-443^50.5%ID^E:5e-26^RecName: Full=Cell division cycle-associated 7-like protein
YS1_GAGATTCC-TATAGCCT_comp15918_seq0	NA	11	.
OL3_CGCTCATT-GGCTCTGA_comp157_seq6	NA	11	COL2_ARATH^COL2_ARATH^Q:3619-4866,H:1-347^47.49%ID^E:2e-91^RecName: Full=Zinc finger protein CONSTANS-LIKE 2
YS2_GAGATTCC-ATAGAGGC_comp20_seq11	NA	11	URH2_ARATH^URH2_ARATH^Q:394-1278,H:6-282^72.88%ID^E:6e-143^RecName: Full=Probable uridine nucleosidase 2
YL1_CGCTCATT-TATAGCCT_comp255_seq0	SGD	11	SG1_RAUSE^SG1_RAUSE^Q:1583-3088,H:32-532^82.7%ID^E:0^RecName: Full=Strictosidine-O-beta-D-glucosidase
YS2_GAGATTCC-ATAGAGGC_comp528_seq2	T16H1	11	C71DC_CATRO^C71DC_CATRO^Q:2140-3633,H:1-493^74.8%ID^E:0^RecName: Full=Tabersonine 16-hydroxylase
YS1_GAGATTCC-TATAGCCT_comp5427_seq0	NA	11	.
OL3_CGCTCATT-GGCTCTGA_comp2293_seq2	NA	12	IPPK_ARATH^IPPK_ARATH^Q:1032-2057,H:104-441^56.43%ID^E:8e-113^RecName: Full=Inositol-pentakisphosphate 2-kinase
YS1_GAGATTCC-TATAGCCT_comp5896_seq1	NA	12	.
YL3_CGCTCATT-ATAGAGGC_comp3968_seq5	NA	12	PDS5B_MOUSE^PDS5B_MOUSE^Q:708-3425,H:161-1075^23.32%ID^E:1e-48^RecName: Full=Sister chromatid cohesion protein PDS5 homolog B
YL1_CGCTCATT-TATAGCCT_comp53601_seq0	NA	12	RUXG_ARATH^RUXG_ARATH^Q:150-386,H:1-79^94.94%ID^E:1e-45^RecName: Full=Probable small nuclear ribonucleoprotein G
YS2_GAGATTCC-ATAGAGGC_comp1805_seq0	NA	12	PLSP1_ARATH^PLSP1_ARATH^Q:457-1044,H:96-291^77.04%ID^E:4e-106^RecName: Full=Chloroplast processing peptidase
YL1_CGCTCATT-TATAGCCT_comp1491_seq4	DXS2	12	DXS2_ORYSJ^DXS2_ORYSJ^Q:147-2255,H:12-712^81.56%ID^E:0^RecName: Full=Probable 1-deoxy-D-xylulose-5-phosphate synthase 2, chloroplastic
YL1_CGCTCATT-TATAGCCT_comp20888_seq0	NA	12	.
YL1_CGCTCATT-TATAGCCT_comp3346_seq5	NA	12	PUB6_ARATH^PUB6_ARATH^Q:588-1658,H:7-381^38.32%ID^E:7e-71^RecName: Full=U-box domain-containing protein 6
YL1_CGCTCATT-TATAGCCT_comp13745_seq0	NA	12	.
YS2_GAGATTCC-ATAGAGGC_comp75153_seq0	NA	13	.
YL1_CGCTCATT-TATAGCCT_comp5567_seq1	NA	13	SIPL1_ARATH^SIPL1_ARATH^Q:240-1358,H:1-372^76.68%ID^E:0^RecName: Full=Signal peptide peptidase-like 1

TableS2

YL3_CGCTCATT-ATAGAGGC_comp2760_seq1	NPF2.9	13	PTR37_ARATH^PTR37_ARATH^Q:293-1912,H:22-548^43.38%ID^E:9e-167^RecName: Full=Protein NRT1/ PTR FAMILY 2.7
YS2_GAGATTCC-ATAGAGGC_comp150_seq7	NA	13	Y1745_ARATH^Y1745_ARATH^Q:750-1634,H:8-285^35.64%ID^E:7e-32^RecName: Full=Uncharacterized protein At1g51745
OL1_CGCTCATT-CCTATCCT_comp6197_seq0	NA	13	ARFA_ARATH^ARFA_ARATH^Q:2-556,H:308-497^48.24%ID^E:8e-37^RecName: Full=Auxin response factor 1
YS2_GAGATTCC-ATAGAGGC_comp6043_seq1	NA	13	SKIP5_ARATH^SKIP5_ARATH^Q:2142-2537,H:130-273^61.81%ID^E:2e-50^RecName: Full=F-box protein SKIP5
YS2_GAGATTCC-ATAGAGGC_comp9205_seq0	NA	13	.
YS2_GAGATTCC-ATAGAGGC_comp14379_seq0	NA	13	H2AV3_ORYSJ^H2AV3_ORYSJ^Q:123-533,H:1-137^83.21%ID^E:1e-60^RecName: Full=Probable histone H2A variant 3
YL1_CGCTCATT-TATAGCCT_comp60629_seq0	NA	13	.
YS2_GAGATTCC-ATAGAGGC_comp1021_seq4	NA	13	PAS2A_ORYSJ^PAS2A_ORYSJ^Q:2278-2910,H:1-211^73.93%ID^E:2e-95^.^.
YS1_GAGATTCC-TATAGCCT_comp17484_seq0	NA	13	SILD_FORIN^SILD_FORIN^Q:1010-1288,H:9-101^61.29%ID^E:8e-23^RecName: Full=Secoisolariciresinol dehydrogenase
OL3_CGCTCATT-GGCTCTGA_comp24855_seq0	NA	14	.
YS1_GAGATTCC-TATAGCCT_comp8170_seq2	NA	14	RSH3C_ARATH^RSH3C_ARATH^Q:1477-1905,H:215-345^47.55%ID^E:1e-24^RecName: Full=Probable GTP diphosphokinase RSH3, chloroplastic
OL1_CGCTCATT-CCTATCCT_comp31_seq2	NA	14	SFH6_ARATH^SFH6_ARATH^Q:1951-2721,H:37-295^62.5%ID^E:9e-115^RecName: Full=Phosphatidylinositol/phosphatidylcholine transfer protein SFH6
OL3_CGCTCATT-GGCTCTGA_comp21501_seq0	NA	14	.
YL1_CGCTCATT-TATAGCCT_comp1669_seq5	NA	14	INO1_TOBAC^INO1_TOBAC^Q:1075-2211,H:132-510^94.2%ID^E:0^RecName: Full=Inositol-3-phosphate synthase
YL1_CGCTCATT-TATAGCCT_comp18714_seq1	NA	14	.
YL1_CGCTCATT-TATAGCCT_comp1354_seq1	GATA1	14	P2C09_ORYSJ^P2C09_ORYSJ^Q:1416-1853,H:252-413^67.28%ID^E:5e-55^RecName: Full=Probable protein phosphatase 2C 9
YL1_CGCTCATT-TATAGCCT_comp20874_seq0	NA	14	.
OL3_CGCTCATT-GGCTCTGA_comp4703_seq4	NA	14	APG3_ARATH^APG3_ARATH^Q:408-1211,H:50-317^83.58%ID^E:3e-149^RecName: Full=Peptide chain release factor APG3, chloroplastic {ECO:0000305}
YL1_CGCTCATT-TATAGCCT_comp15844_seq2	NA	14	.
AR1_GAGATTCC-CCTATCCT_comp303_seq2	NA	15	.
AR1_GAGATTCC-CCTATCCT_comp521_seq0	ZCT3	15	ZAT10_ARATH^ZAT10_ARATH^Q:199-912,H:1-223^49.8%ID^E:8e-57^RecName: Full=Zinc finger protein ZAT10
AR1_GAGATTCC-CCTATCCT_comp814_seq11	NA	15	PGLR_VITVI^PGLR_VITVI^Q:2138-3325,H:59-455^51.39%ID^E:5e-124^RecName: Full=Probable polygalacturonase
AR1_GAGATTCC-CCTATCCT_comp1653_seq0	NA	15	PLA2A_ARATH^PLA2A_ARATH^Q:172-600,H:3-145^55.17%ID^E:3e-50^RecName: Full=Phospholipase A2-alpha {ECO:0000303|PubMed:16140037}
AR1_GAGATTCC-CCTATCCT_comp2186_seq5	NA	15	B3GT7_ARATH^B3GT7_ARATH^Q:740-1732,H:88-308^52.11%ID^E:1e-79^RecName: Full=Beta-1,3-galactosyltransferase 7
AR1_GAGATTCC-CCTATCCT_comp737_seq2	ZCT1	15	ZAT12_ARATH^ZAT12_ARATH^Q:298-639,H:5-109^57.39%ID^E:2e-29^RecName: Full=Zinc finger protein ZAT12
YL3_CGCTCATT-ATAGAGGC_comp1769_seq0	NA	15	PUMP5_ARATH^PUMP5_ARATH^Q:384-1355,H:1-311^73.39%ID^E:4e-146^RecName: Full=Mitochondrial uncoupling protein 5
AR1_GAGATTCC-CCTATCCT_comp3339_seq0	NA	15	.
AR1_GAGATTCC-CCTATCCT_comp20837_seq1	NA	15	WRK33_ARATH^WRK33_ARATH^Q:514-909,H:194-328^56.74%ID^E:8e-26^RecName: Full=Probable WRKY transcription factor 33
YS1_GAGATTCC-TATAGCCT_comp11393_seq0	NA	15	.
AR2_GAGATTCC-GGCTCTGA_comp5642_seq3	NA	15	E70A1_ARATH^E70A1_ARATH^Q:306-2003,H:66-627^29.11%ID^E:4e-77^RecName: Full=Exocyst complex component EXO70A1
AR1_GAGATTCC-CCTATCCT_comp19692_seq0	NA	15	.
YL1_CGCTCATT-TATAGCCT_comp1138_seq1	NA	15	XTH2_SOYBN^XTH2_SOYBN^Q:154-936,H:30-283^66.28%ID^E:6e-117^RecName: Full=Xyloglucan endotransglucosylase/hydrolase 2 {ECO:0000250|UniProtKB:Q38857}
YL1_CGCTCATT-TATAGCCT_comp2433_seq0	NA	15	CML31_ORYSJ^CML31_ORYSJ^Q:354-752,H:15-150^49.26%ID^E:5e-32^RecName: Full=Probable calcium-binding protein CML31
AR1_GAGATTCC-CCTATCCT_comp15923_seq0	NA	15	.
AR1_GAGATTCC-CCTATCCT_comp15349_seq0	NA	15	.
AR2_GAGATTCC-GGCTCTGA_comp25525_seq0	NA	15	.
YL1_CGCTCATT-TATAGCCT_comp1591_seq0	NA	15	RAP24_ARATH^RAP24_ARATH^Q:953-1651,H:76-329^56.03%ID^E:7e-60^RecName: Full=Ethylene-responsive transcription factor RAP2-4
AR1_GAGATTCC-CCTATCCT_comp3391_seq0	NA	15	.
AR1_GAGATTCC-CCTATCCT_comp19574_seq0	NA	15	.
YL1_CGCTCATT-TATAGCCT_comp8028_seq1	NA	15	NAC81_ARATH^NAC81_ARATH^Q:626-901,H:54-147^90.43%ID^E:2e-39^RecName: Full=Protein ATAF2
AR1_GAGATTCC-CCTATCCT_comp21765_seq0	NA	15	.
AR2_GAGATTCC-GGCTCTGA_comp3473_seq2	NA	15	WRK40_ARATH^WRK40_ARATH^Q:1211-1579,H:183-302^48.82%ID^E:3e-24^RecName: Full=Probable WRKY transcription factor 40
YL1_CGCTCATT-TATAGCCT_comp20940_seq0	NA	15	.
AR2_GAGATTCC-GGCTCTGA_comp1098_seq4	NA	15	RTNLQ_ARATH^RTNLQ_ARATH^Q:222-1385,H:4-373^50%ID^E:1e-91^RecName: Full=Reticulon-like protein B17

AR1_GAGATTCC-CCTATCCT_comp26846_seq1	NA	15	.
YL1_CGCTCATT-TATAGCCT_comp34042_seq0	NA	15	.
OL3_CGCTCATT-GGCTCTGA_comp30250_seq0	NA	15	RS13_SOYBN^RS13_SOYBN^Q:100-519,H:1-140^92.86%ID^E:5e-86^RecName: Full=40S ribosomal protein S13
AR2_GAGATTCC-GGCTCTGA_comp2596_seq1	NA	15	RING1_GOSHI^RING1_GOSHI^Q:682-1068,H:163-287^46.15%ID^E:1e-31^RecName: Full=E3 ubiquitin-protein ligase RING1
AR1_GAGATTCC-CCTATCCT_comp21983_seq0	NA	15	.
OL3_CGCTCATT-GGCTCTGA_comp62954_seq0	NA	15	ARFF_ORYSJ^ARFF_ORYSJ^Q:192-365,H:164-221^89.66%ID^E:3e-51^RecName: Full=Auxin response factor 6
YL1_CGCTCATT-TATAGCCT_comp2206_seq7	NA	15	WRK40_ARATH^WRK40_ARATH^Q:1321-1728,H:165-302^44.59%ID^E:1e-21^RecName: Full=Probable WRKY transcription factor 40
AR2_GAGATTCC-GGCTCTGA_comp656_seq7	NA	15	COBRA_ARATH^COBRA_ARATH^Q:469-1668,H:35-435^83.79%ID^E:0^RecName: Full=Protein COBRA
AR1_GAGATTCC-CCTATCCT_comp2897_seq1	NA	15	.
AR2_GAGATTCC-GGCTCTGA_comp630_seq3	CrERF5	15	ERF5_NICSY^ERF5_NICSY^Q:157-1017,H:1-282^46.28%ID^E:6e-47^RecName: Full=Ethylene-responsive transcription factor 5
YL1_CGCTCATT-TATAGCCT_comp1014_seq1	NA	16	QCR72_ARATH^QCR72_ARATH^Q:416-775,H:3-122^73.33%ID^E:2e-53^RecName: Full=Cytochrome b-c1 complex subunit 7-2
AR2_GAGATTCC-GGCTCTGA_comp36070_seq0	NA	16	VAP12_ARATH^VAP12_ARATH^Q:1450-2037,H:40-221^62.63%ID^E:1e-71^RecName: Full=Vesicle-associated protein 1-2
AR2_GAGATTCC-GGCTCTGA_comp10915_seq2	NA	16	.
AR1_GAGATTCC-CCTATCCT_comp4311_seq2	NA	16	.
YL1_CGCTCATT-TATAGCCT_comp32_seq1	GO	16	C7BL3_CICIN^C7BL3_CICIN^Q:361-1737,H:37-488^38.7%ID^E:9e-111^RecName: Full=Costunolide synthase
YS2_GAGATTCC-ATAGAGGC_comp7642_seq3	NA	16	HCS2_ARATH^HCS2_ARATH^Q:280-1245,H:9-329^65.02%ID^E:1e-143^RecName: Full=Biotin--protein ligase 2
OL1_CGCTCATT-CCTATCCT_comp6428_seq0	NA	16	M2K9_ARATH^M2K9_ARATH^Q:288-1262,H:1-308^60.43%ID^E:2e-114^RecName: Full=Mitogen-activated protein kinase kinase 9
YL1_CGCTCATT-TATAGCCT_comp32588_seq0	NA	16	.
OL3_CGCTCATT-GGCTCTGA_comp1418_seq0	T19H	17	C71A2_SOLME^C71A2_SOLME^Q:587-1945,H:46-503^50.33%ID^E:6e-151^RecName: Full=Cytochrome P450 71A2
OL3_CGCTCATT-GGCTCTGA_comp132_seq7	NA	17	FRO6_ARATH^FRO6_ARATH^Q:3030-4742,H:189-738^57.9%ID^E:0^RecName: Full=Ferric reduction oxidase 6
OL3_CGCTCATT-GGCTCTGA_comp15162_seq0	NA	17	.
YL1_CGCTCATT-TATAGCCT_comp12179_seq1	NA	17	BOR4_ARATH^BOR4_ARATH^Q:168-2138,H:6-669^69.66%ID^E:0^RecName: Full=Boron transporter 4
OL3_CGCTCATT-GGCTCTGA_comp7365_seq1	NA	17	RNHX1_ARATH^RNHX1_ARATH^Q:783-2540,H:1-584^25.62%ID^E:5e-41^RecName: Full=Putative ribonuclease H protein At1g65750
OL3_CGCTCATT-GGCTCTGA_comp1249_seq0	NA	17	.
OL1_CGCTCATT-CCTATCCT_comp119_seq1	NA	17	.
OL1_CGCTCATT-CCTATCCT_comp368_seq0	NA	18	METE_CATRO^METE_CATRO^Q:156-2450,H:1-765^95.16%ID^E:0^RecName: Full=5-methyltetrahydropteroyltriglutamate--homocysteine methyltransferase
AR2_GAGATTCC-GGCTCTGA_comp3587_seq0	NA	18	IDH1_ARATH^IDH1_ARATH^Q:613-1503,H:67-367^87.71%ID^E:8e-171^RecName: Full=Isocitrate dehydrogenase [NAD] regulatory subunit 1, mitochondrial
YL3_CGCTCATT-ATAGAGGC_comp5981_seq5	NA	18	APY2_ARATH^APY2_ARATH^Q:2126-2938,H:201-471^71.59%ID^E:1e-120^RecName: Full=Apyrase 2
YS2_GAGATTCC-ATAGAGGC_comp1097_seq5	NA	18	.
AR1_GAGATTCC-CCTATCCT_comp6659_seq0	NA	18	.
AR2_GAGATTCC-GGCTCTGA_comp7061_seq1	NA	18	PXL1_ARATH^PXL1_ARATH^Q:167-3247,H:1-1029^63.89%ID^E:0^RecName: Full=Leucine-rich repeat receptor-like protein kinase PXL1
YL1_CGCTCATT-TATAGCCT_comp13796_seq1	NA	18	S61G2_ARATH^S61G2_ARATH^Q:3-206,H:2-69^89.71%ID^E:9e-23^RecName: Full=Protein transport protein Sec61 subunit gamma-2
AR1_GAGATTCC-CCTATCCT_comp2488_seq0	NA	18	ACS_ARATH^ACS_ARATH^Q:1426-1989,H:540-727^87.77%ID^E:8e-94^RecName: Full=Acetyl-coenzyme A synthetase, chloroplastic/glyoxysomal
AR2_GAGATTCC-GGCTCTGA_comp5665_seq0	ZCT2	18	PI3K_ARATH^PI3K_ARATH^Q:1-309,H:712-814^95.15%ID^E:4e-57^RecName: Full=Phosphatidylinositol 3-kinase VPS34
AR1_GAGATTCC-CCTATCCT_comp7698_seq2	NA	18	BIG1_ARATH^BIG1_ARATH^Q:1905-7001,H:2-1685^66.3%ID^E:0^RecName: Full=Brefeldin A-inhibited guanine nucleotide-exchange protein 1
AR1_GAGATTCC-CCTATCCT_comp2717_seq1	NA	18	TMCO1_RAT^TMCO1_RAT^Q:195-743,H:5-184^44.15%ID^E:5e-40^RecName: Full=Transmembrane and coiled-coil domains protein 1
YL3_CGCTCATT-ATAGAGGC_comp3830_seq1	NA	18	.
AR1_GAGATTCC-CCTATCCT_comp8039_seq0	GPPS	18	SPS3_ARATH^SPS3_ARATH^Q:300-1559,H:1-422^66.59%ID^E:0^RecName: Full=Solanesyl diphosphate synthase 3, chloroplastic/mitochondrial
AR1_GAGATTCC-CCTATCCT_comp17606_seq0	NA	18	.
YS2_GAGATTCC-ATAGAGGC_comp287_seq1	HDS	18	ISPG_ARATH^ISPG_ARATH^Q:239-2449,H:1-737^86.02%ID^E:0^RecName: Full=4-hydroxy-3-methylbut-2-en-1-yl diphosphate synthase (ferredoxin), chloroplastic
AR2_GAGATTCC-GGCTCTGA_comp1042_seq4	NA	18	.
AR1_GAGATTCC-CCTATCCT_comp1722_seq0	NA	18	THCAS_CANSA^THCAS_CANSA^Q:162-1625,H:46-540^45.96%ID^E:2e-130^RecName: Full=Tetrahydrocannabinolic acid synthase
OL3_CGCTCATT-GGCTCTGA_comp4392_seq1	NA	18	CYC_ABUTH^CYC_ABUTH^Q:140-472,H:1-111^94.59%ID^E:2e-70^RecName: Full=Cytochrome c
YL1_CGCTCATT-TATAGCCT_comp6904_seq2	NA	18	INVA_VICFA^INVA_VICFA^Q:6713-8377,H:71-630^70%ID^E:0^RecName: Full=Acid beta-fructofuranosidase

TableS2

YL3_CGCTCATT-ATAGAGGC_comp826_seq0	NA	18	14334_SOLLC^14334_SOLLC^Q:652-1428,H:4-260^79.15%ID^E:9e-140^RecName: Full=14-3-3 protein 4
YL3_CGCTCATT-ATAGAGGC_comp116_seq0	NA	18	RL9_PEA^RL9_PEA^Q:447-815,H:1-123^82.11%ID^E:7e-67^RecName: Full=60S ribosomal protein L9
YL3_CGCTCATT-ATAGAGGC_comp3473_seq0	NA	18	METK1_CATRO^METK1_CATRO^Q:99-1277,H:1-393^96.95%ID^E:0^RecName: Full=S-adenosylmethionine synthase 1
AR1_GAGATTCC-CCTATCCT_comp1014_seq2	NA	18	NOTUM_MOUSE^NOTUM_MOUSE^Q:2970-3719,H:106-368^29.7%ID^E:3e-23^RecName: Full=Palmitoleoyl-protein carboxylesterase NOTUM {ECO:0000250|UniProtKB:Q6P988}
YL3_CGCTCATT-ATAGAGGC_comp56582_seq0	NA	18	PKHA8_RAT^PKHA8_RAT^Q:531-965,H:327-471^34.93%ID^E:6e-21^RecName: Full=Pleckstrin homology domain-containing family A member 8
AR1_GAGATTCC-CCTATCCT_comp46_seq0	NA	18	SAHH_CATRO^SAHH_CATRO^Q:259-1713,H:1-485^94.85%ID^E:0^RecName: Full=Adenosylhomocysteinase
AR1_GAGATTCC-CCTATCCT_comp12098_seq0	NA	18	UBP1_ARATH^UBP1_ARATH^Q:934-1974,H:785-1083^40.17%ID^E:2e-56^RecName: Full=Ubiquitin carboxyl-terminal hydrolase 1
OL1_CGCTCATT-CCTATCCT_comp1720_seq1	NA	19	FLS_PETHY^FLS_PETHY^Q:113-1117,H:13-348^75%ID^E:2e-157^RecName: Full=Flavonol synthase/flavanone 3-hydroxylase
YS2_GAGATTCC-ATAGAGGC_comp9582_seq0	NA	19	HYES_HUMAN^HYES_HUMAN^Q:110-1027,H:237-544^33.02%ID^E:4e-36^RecName: Full=Bifunctional epoxide hydrolase 2
OL1_CGCTCATT-CCTATCCT_comp520_seq1	IO	19	C76A2_SOLME^C76A2_SOLME^Q:383-1777,H:43-503^50.97%ID^E:6e-168^RecName: Full=Cytochrome P450 76A2
YS1_GAGATTCC-TATAGCCT_comp5894_seq4	NA	19	PERK1_ARATH^PERK1_ARATH^Q:1022-2218,H:252-651^79.25%ID^E:0^RecName: Full=Proline-rich receptor-like protein kinase PERK1
AR1_GAGATTCC-CCTATCCT_comp37384_seq0	NA	19	.
YL1_CGCTCATT-TATAGCCT_comp24385_seq0	NA	19	TRBP2_ARATH^TRBP2_ARATH^Q:3-227,H:165-239^98.67%ID^E:2e-39^RecName: Full=Tryptophan synthase beta chain 2, chloroplastic
YL3_CGCTCATT-ATAGAGGC_comp17087_seq0	NA	19	.
YS2_GAGATTCC-ATAGAGGC_comp5954_seq0	NA	20	E70B1_ARATH^E70B1_ARATH^Q:239-2152,H:1-620^64.75%ID^E:0^RecName: Full=Exocyst complex component EXO70B1
YL3_CGCTCATT-ATAGAGGC_comp11008_seq1	NA	20	.
OL3_CGCTCATT-GGCTCTGA_comp13259_seq1	NA	20	CLPX_SPHWW^CLPX_SPHWW^Q:642-1646,H:65-404^54.55%ID^E:4e-118^RecName: Full=ATP-dependent Clp protease ATP-binding subunit ClpX {ECO:0000255|HAMAP-Rule:MF_00175}
YL3_CGCTCATT-ATAGAGGC_comp32910_seq0	NA	20	.
YS2_GAGATTCC-ATAGAGGC_comp6479_seq1	NA	20	ADT1_GOSHI^ADT1_GOSHI^Q:155-1318,H:1-386^83.76%ID^E:0^RecName: Full=ADP,ATP carrier protein 1, mitochondrial
AR1_GAGATTCC-CCTATCCT_comp3108_seq0	T3R	20	10HGO_CATRO^10HGO_CATRO^Q:164-1228,H:2-360^63.51%ID^E:1e-145^RecName: Full=8-hydroxygeraniol dehydrogenase
OL1_CGCTCATT-CCTATCCT_comp209_seq10	NA	20	.
YS1_GAGATTCC-TATAGCCT_comp6840_seq0	NA	20	.
YL3_CGCTCATT-ATAGAGGC_comp30598_seq0	NA	20	.
AR2_GAGATTCC-GGCTCTGA_comp29260_seq0	NA	21	.
AR1_GAGATTCC-CCTATCCT_comp14477_seq0	ORCA3	21	ERF99_ARATH^ERF99_ARATH^Q:401-604,H:70-136^72.06%ID^E:6e-25^RecName: Full=Ethylene-responsive transcription factor 13
AR1_GAGATTCC-CCTATCCT_comp6627_seq1	NA	21	.
AR1_GAGATTCC-CCTATCCT_comp18379_seq1	NA	21	.
AR1_GAGATTCC-CCTATCCT_comp2945_seq0	NA	21	RA51B_ARATH^RA51B_ARATH^Q:6971-8011,H:26-370^66.57%ID^E:4e-122^RecName: Full=DNA repair protein RAD51 homolog 2
YS2_GAGATTCC-ATAGAGGC_comp9578_seq1	NA	21	Y4374_ARATH^Y4374_ARATH^Q:320-2167,H:18-635^56.05%ID^E:0^RecName: Full=Probable inactive receptor kinase At4g23740
OL3_CGCTCATT-GGCTCTGA_comp25748_seq0	NA	22	.
YS1_GAGATTCC-TATAGCCT_comp40843_seq0	NA	22	.
YS1_GAGATTCC-TATAGCCT_comp8051_seq0	NA	22	.
OL3_CGCTCATT-GGCTCTGA_comp21659_seq0	NA	22	.
AR2_GAGATTCC-GGCTCTGA_comp12782_seq0	NA	22	.
OL3_CGCTCATT-GGCTCTGA_comp34622_seq0	NA	22	.
OL3_CGCTCATT-GGCTCTGA_comp2285_seq1	NPF2.1	22	PTR21_ARATH^PTR21_ARATH^Q:684-2408,H:44-609^54.43%ID^E:0^RecName: Full=Protein NRT1/ PTR FAMILY 2.13
YS1_GAGATTCC-TATAGCCT_comp64350_seq0	NA	22	.
YL1_CGCTCATT-TATAGCCT_comp16103_seq0	NA	22	PEX13_ARATH^PEX13_ARATH^Q:451-735,H:202-304^66.02%ID^E:1e-27^RecName: Full=Peroxisomal membrane protein 13
YS2_GAGATTCC-ATAGAGGC_comp11235_seq0	NA	23	CX32_ARATH^CX32_ARATH^Q:640-1569,H:63-369^69.03%ID^E:2e-150^RecName: Full=Probable serine/threonine-protein kinase Cx32, chloroplastic
OL3_CGCTCATT-GGCTCTGA_comp18596_seq0	NA	23	.
OL1_CGCTCATT-CCTATCCT_comp22262_seq0	NA	23	BPS1_ARATH^BPS1_ARATH^Q:994-1986,H:1-326^56.12%ID^E:4e-123^RecName: Full=Protein BPS1, chloroplastic
OL3_CGCTCATT-GGCTCTGA_comp2267_seq1	VmTPT2	23	AB32G_ARATH^AB32G_ARATH^Q:1321-5580,H:1-1418^70.34%ID^E:0^RecName: Full=ABC transporter G family member 32 {ECO:0000303|PubMed:18299247}
YL1_CGCTCATT-TATAGCCT_comp12043_seq2	NA	23	BCS1_SCHPO^BCS1_SCHPO^Q:1059-1733,H:195-423^29.05%ID^E:1e-24^RecName: Full=Probable mitochondrial chaperone bcs1
OL1_CGCTCATT-CCTATCCT_comp671_seq2	NA	23	SEC8_ARATH^SEC8_ARATH^Q:2372-5161,H:143-1053^68.17%ID^E:0^RecName: Full=Exocyst complex component SEC8

OL1_CGCTCATT-CCTATCCT_comp13536_seq1	NA	23	.
YS1_GAGATTCC-TATAGCCT_comp17664_seq1	NA	23	P2C51_ARATH^P2C51_ARATH^Q:134-1405,H:30-447^54.55%ID^E:1e-139^RecName: Full=Probable protein phosphatase 2C 51
OL3_CGCTCATT-GGCTCTGA_comp2330_seq3	NA	24	.
YL3_CGCTCATT-ATAGAGGC_comp15500_seq0	NA	24	Y1680_ARATH^Y1680_ARATH^Q:2339-4210,H:30-648^42.17%ID^E:9e-86^RecName: Full=Probable leucine-rich repeat receptor-like protein kinase At1g68400
YL1_CGCTCATT-TATAGCCT_comp8450_seq0	NA	24	YERG_SCHPO^YERG_SCHPO^Q:37-378,H:204-320^42.74%ID^E:8e-23^RecName: Full=Uncharacterized RING finger protein C2F3.16
YS1_GAGATTCC-TATAGCCT_comp15900_seq1	NA	24	SIGD_ARATH^SIGD_ARATH^Q:957-1427,H:258-414^54.78%ID^E:9e-82^RecName: Full=RNA polymerase sigma factor sigD, chloroplastic
OL3_CGCTCATT-GGCTCTGA_comp3385_seq1	NA	24	.
YS2_GAGATTCC-ATAGAGGC_comp1361_seq4	NA	24	CYB5E_ARATH^CYB5E_ARATH^Q:2054-2449,H:2-133^81.82%ID^E:2e-70^RecName: Full=Cytochrome b5 isoform E {ECO:0000303|PubMed:19054355}
OL3_CGCTCATT-GGCTCTGA_comp8789_seq0	NPF2.4	24	PTR53_ARATH^PTR53_ARATH^Q:121-1830,H:27-597^57.62%ID^E:0^RecName: Full=Protein NRT1/ PTR FAMILY 2.11
OL3_CGCTCATT-GGCTCTGA_comp35783_seq0	NA	24	.
OL3_CGCTCATT-GGCTCTGA_comp19373_seq0	NA	24	.
AR2_GAGATTCC-GGCTCTGA_comp2182_seq0	NA	25	XCP1_ARATH^XCP1_ARATH^Q:155-1150,H:26-353^72.59%ID^E:0^RecName: Full=Xylem cysteine proteinase 1
YS1_GAGATTCC-TATAGCCT_comp17355_seq0	NA	25	.
YL3_CGCTCATT-ATAGAGGC_comp25012_seq1	NA	25	CSLCC_ARATH^CSLCC_ARATH^Q:1254-3374,H:1-699^78.22%ID^E:0^RecName: Full=Probable xyloglucan glycosyltransferase 12
YS2_GAGATTCC-ATAGAGGC_comp3345_seq1	MATE2	25	ALIS3_ARATH^ALIS3_ARATH^Q:2756-3742,H:22-349^75.99%ID^E:8e-171^RecName: Full=ALA-interacting subunit 3
AR2_GAGATTCC-GGCTCTGA_comp901_seq0	NA	25	.
AR1_GAGATTCC-CCTATCCT_comp2521_seq4	NA	25	UPL3_ARATH^UPL3_ARATH^Q:848-6109,H:151-1888^71.65%ID^E:0^RecName: Full=E3 ubiquitin-protein ligase UPL3
YS1_GAGATTCC-TATAGCCT_comp1496_seq2	CMK	26	ISPE_SOLLC^ISPE_SOLLC^Q:362-1471,H:31-399^75.95%ID^E:2e-175^RecName: Full=4-diphosphocytidyl-2-C-methyl-D-erythritol kinase, chloroplastic/chromoplastic
AR2_GAGATTCC-GGCTCTGA_comp7989_seq1	NA	26	.
AR1_GAGATTCC-CCTATCCT_comp4372_seq0	NA	26	.
YS2_GAGATTCC-ATAGAGGC_comp147_seq0	NA	26	RS33_ARATH^RS33_ARATH^Q:169-834,H:1-222^87.84%ID^E:3e-142^RecName: Full=40S ribosomal protein S3-3
YL1_CGCTCATT-TATAGCCT_comp29003_seq0	NA	26	.
AR1_GAGATTCC-CCTATCCT_comp1690_seq3	NA	26	PMT2_ARATH^PMT2_ARATH^Q:374-2215,H:1-616^76.45%ID^E:0^RecName: Full=Probable methyltransferase PMT2
YS2_GAGATTCC-ATAGAGGC_comp21925_seq0	NA	26	.
AR1_GAGATTCC-CCTATCCT_comp3686_seq1	NA	27	.
AR1_GAGATTCC-CCTATCCT_comp15754_seq0	NA	27	.
AR1_GAGATTCC-CCTATCCT_comp10689_seq2	DAT	27	DAT_CATRO^DAT_CATRO^Q:1-1251,H:5-437^54.71%ID^E:5e-150^RecName: Full=Deacetylvindoline O-acetyltransferase
AR2_GAGATTCC-GGCTCTGA_comp216_seq0	NA	27	.
AR2_GAGATTCC-GGCTCTGA_comp22743_seq0	NA	27	.
OL3_CGCTCATT-GGCTCTGA_comp5631_seq2	GBF2	28	HBP1A_WHEAT^HBP1A_WHEAT^Q:439-1467,H:18-319^45.3%ID^E:4e-54^RecName: Full=Transcription factor HBP-1a
AR2_GAGATTCC-GGCTCTGA_comp2924_seq0	NA	28	.
AR1_GAGATTCC-CCTATCCT_comp4693_seq1	NA	28	.
YL3_CGCTCATT-ATAGAGGC_comp15371_seq1	NA	28	RDR5_ARATH^RDR5_ARATH^Q:546-3176,H:114-975^48.45%ID^E:0^RecName: Full=Probable RNA-dependent RNA polymerase 5
YL3_CGCTCATT-ATAGAGGC_comp4673_seq1	NA	28	NSA2_HUMAN^NSA2_HUMAN^Q:2-265,H:149-236^78.41%ID^E:1e-42^RecName: Full=Ribosome biogenesis protein NSA2 homolog
AR2_GAGATTCC-GGCTCTGA_comp9703_seq0	NA	28	INVB_DAUCA^INVB_DAUCA^Q:1146-3059,H:4-656^67.48%ID^E:0^RecName: Full=Beta-fructofuranosidase, soluble isoenzyme I
YS2_GAGATTCC-ATAGAGGC_comp6465_seq1	NA	29	Y283_BACHD^Y283_BACHD^Q:829-1725,H:1-265^39.33%ID^E:1e-58^RecName: Full=Uncharacterized isomerase BH0283
YL3_CGCTCATT-ATAGAGGC_comp37282_seq0	NA	29	.
AR1_GAGATTCC-CCTATCCT_comp12730_seq0	NA	29	.
YL3_CGCTCATT-ATAGAGGC_comp1199_seq2	NA	29	GSTX4_TOBAC^GSTX4_TOBAC^Q:401-1045,H:5-220^68.52%ID^E:6e-101^RecName: Full=Probable glutathione S-transferase
YL3_CGCTCATT-ATAGAGGC_comp3815_seq0	NA	29	CSK2C_ARATH^CSK2C_ARATH^Q:57-521,H:96-250^80%ID^E:2e-89^RecName: Full=Casein kinase II subunit beta'
YS2_GAGATTCC-ATAGAGGC_comp88_seq3	LAMT	29	MT878_ARATH^MT878_ARATH^Q:389-1450,H:3-361^41.1%ID^E:4e-79^RecName: Full=Probable S-adenosylmethionine-dependent methyltransferase At5g38780
YS1_GAGATTCC-TATAGCCT_comp3264_seq4	NA	29	LOR12_ARATH^LOR12_ARATH^Q:1818-2432,H:9-205^50%ID^E:5e-54^RecName: Full=Protein LURP-one-related 12
AR2_GAGATTCC-GGCTCTGA_comp32417_seq0	NA	29	.
AR1_GAGATTCC-CCTATCCT_comp19025_seq0	NA	29	C2D61_ARATH^C2D61_ARATH^Q:2-235,H:285-362^79.49%ID^E:4e-34^RecName: Full=C2 domain-containing protein At1g53590

AR1_GAGATTCC-CCTATCCT_comp4621_seq0	NA	29	BB_ARATH^BB_ARATH^Q:1423-1734,H:138-241^66.67%ID^E:5e-39^RecName: Full=E3 ubiquitin ligase BIG BROTHER
AR1_GAGATTCC-CCTATCCT_comp2309_seq1	NA	29	HHP2_ARATH^HHP2_ARATH^Q:519-1337,H:75-356^60.07%ID^E:4e-79^RecName: Full=Heptahelical transmembrane protein 2
YS1_GAGATTCC-TATAGCCT_comp9272_seq0	NA	29	KPYC_SOLTU^KPYC_SOLTU^Q:840-1322,H:347-506^41.61%ID^E:6e-31^RecName: Full=Pyruvate kinase, cytosolic isozyme
YS1_GAGATTCC-TATAGCCT_comp439_seq2	HDR	30	ISPH_ARATH^ISPH_ARATH^Q:147-1547,H:1-466^78.09%ID^E:0^RecName: Full=4-hydroxy-3-methylbut-2-enyl diphosphate reductase, chloroplastic
OL1_CGCTCATT-CCTATCCT_comp161_seq5	NA	30	FRO7_ARATH^FRO7_ARATH^Q:3155-5278,H:32-747^59.58%ID^E:0^RecName: Full=Ferric reduction oxidase 7, chloroplastic
OL1_CGCTCATT-CCTATCCT_comp991_seq7	NA	30	.
OL1_CGCTCATT-CCTATCCT_comp5868_seq2	NA	30	MOC2B_ARATH^MOC2B_ARATH^Q:1951-2445,H:18-183^66.87%ID^E:7e-57^RecName: Full=Molybdopterin synthase catalytic subunit {ECO:0000255|HAMAP-Rule:MF_03052}
YS2_GAGATTCC-ATAGAGGC_comp6690_seq0	NA	30	PERR_RAUSE^PERR_RAUSE^Q:232-1107,H:1-289^74.66%ID^E:2e-154^RecName: Full=Perakine reductase
OL1_CGCTCATT-CCTATCCT_comp9509_seq1	NA	30	Y4885_ARATH^Y4885_ARATH^Q:309-2498,H:312-1041^42.57%ID^E:2e-172^RecName: Full=Probable LRR receptor-like serine/threonine-protein kinase At4g08850
YL1_CGCTCATT-TATAGCCT_comp615_seq1	NA	30	STR4_ARATH^STR4_ARATH^Q:734-1729,H:1-321^62.46%ID^E:5e-87^RecName: Full=Rhodanese-like domain-containing protein 4, chloroplastic
YS2_GAGATTCC-ATAGAGGC_comp9283_seq0	NA	30	Y1480_ARATH^Y1480_ARATH^Q:312-1265,H:55-357^39.69%ID^E:3e-59^RecName: Full=Uncharacterized protein At1g18480
YL1_CGCTCATT-TATAGCCT_comp4903_seq0	NA	30	NIFU1_ARATH^NIFU1_ARATH^Q:212-703,H:66-230^79.39%ID^E:3e-63^RecName: Full=NifU-like protein 1, chloroplastic
OL1_CGCTCATT-CCTATCCT_comp7103_seq2	NA	30	RH41_ARATH^RH41_ARATH^Q:768-2225,H:12-490^59.8%ID^E:0^RecName: Full=DEAD-box ATP-dependent RNA helicase 41
YL3_CGCTCATT-ATAGAGGC_comp1376_seq3	NA	30	RVE8_ARATH^RVE8_ARATH^Q:203-565,H:31-151^82.64%ID^E:6e-47^RecName: Full=Protein REVEILLE 8
YL1_CGCTCATT-TATAGCCT_comp3675_seq3	NA	30	SIGE_ARATH^SIGE_ARATH^Q:721-2280,H:1-516^62.93%ID^E:0^RecName: Full=RNA polymerase sigma factor sigE, chloroplastic/mitochondrial
YL1_CGCTCATT-TATAGCCT_comp12033_seq1	NA	31	C90A1_ARATH^C90A1_ARATH^Q:285-1586,H:41-468^75.86%ID^E:0^RecName: Full=Cytochrome P450 90A1
YS2_GAGATTCC-ATAGAGGC_comp2127_seq1	DXS1	31	PWD_ARATH^PWD_ARATH^Q:2912-6307,H:48-1196^63.9%ID^E:0^RecName: Full=Phosphoglucan, water dikinase, chloroplastic
YL3_CGCTCATT-ATAGAGGC_comp4506_seq1	NA	31	.
YS2_GAGATTCC-ATAGAGGC_comp11659_seq0	NA	31	.
YS2_GAGATTCC-ATAGAGGC_comp8026_seq0	NA	31	.
YL1_CGCTCATT-TATAGCCT_comp11163_seq2	NA	31	.
YL3_CGCTCATT-ATAGAGGC_comp38593_seq0	NA	31	SYE_ARATH^SYE_ARATH^Q:709-1746,H:223-568^76.37%ID^E:0^.^.
AR2_GAGATTCC-GGCTCTGA_comp894_seq1	NA	32	.
AR1_GAGATTCC-CCTATCCT_comp8273_seq2	NA	32	S38A7_DANRE^S38A7_DANRE^Q:251-1336,H:56-409^25.33%ID^E:2e-25^RecName: Full=Putative sodium-coupled neutral amino acid transporter 7
YS1_GAGATTCC-TATAGCCT_comp13751_seq2	TS	32	LAX3_MEDTR^LAX3_MEDTR^Q:2-1273,H:42-465^89.15%ID^E:0^RecName: Full=Auxin transporter-like protein 3
AR2_GAGATTCC-GGCTCTGA_comp3252_seq1	NA	32	CTR1_ARATH^CTR1_ARATH^Q:2705-3418,H:562-805^49.59%ID^E:2e-69^RecName: Full=Serine/threonine-protein kinase CTR1 {ECO:0000303|PubMed:8431946}
YS2_GAGATTCC-ATAGAGGC_comp2275_seq1	NA	32	PPME1_MOUSE^PPME1_MOUSE^Q:263-1162,H:13-372^43.61%ID^E:2e-93^RecName: Full=Protein phosphatase methylesterase 1
YL1_CGCTCATT-TATAGCCT_comp13794_seq0	NA	33	.
YS1_GAGATTCC-TATAGCCT_comp3725_seq0	GBF1	33	CPRF1_PETCR^CPRF1_PETCR^Q:479-1702,H:1-406^54.33%ID^E:8e-136^RecName: Full=Common plant regulatory factor 1
YS1_GAGATTCC-TATAGCCT_comp19403_seq0	NA	33	.
YS1_GAGATTCC-TATAGCCT_comp15574_seq0	NA	33	CFI_CAMSI^CFI_CAMSI^Q:122-673,H:37-219^70.65%ID^E:2e-89^RecName: Full=Chalcone--flavonone isomerase
YL1_CGCTCATT-TATAGCCT_comp11427_seq0	NA	33	DCP1_ARATH^DCP1_ARATH^Q:257-907,H:1-211^77.52%ID^E:2e-108^RecName: Full=mRNA-decapping enzyme-like protein
AR2_GAGATTCC-GGCTCTGA_comp24852_seq0	NA	34	.
AR1_GAGATTCC-CCTATCCT_comp13415_seq0	NA	34	HFA6B_ARATH^HFA6B_ARATH^Q:459-1457,H:47-402^50.68%ID^E:5e-106^RecName: Full=Heat stress transcription factor A-6b
AR1_GAGATTCC-CCTATCCT_comp31298_seq0	NA	34	.
AR1_GAGATTCC-CCTATCCT_comp42375_seq0	NA	34	.
AR1_GAGATTCC-CCTATCCT_comp25166_seq0	NA	34	.
AR1_GAGATTCC-CCTATCCT_comp821_seq3	NA	34	PER5_VITVI^PER5_VITVI^Q:296-1213,H:26-328^55.56%ID^E:8e-105^RecName: Full=Peroxidase 5 {ECO:0000250|UniProtKB:P22195}
AR2_GAGATTCC-GGCTCTGA_comp15931_seq0	NA	34	PHT17_ORYSJ^PHT17_ORYSJ^Q:3-218,H:211-284^67.57%ID^E:9e-24^RecName: Full=Probable inorganic phosphate transporter 1-7
AR2_GAGATTCC-GGCTCTGA_comp370_seq0	NA	34	14KD_DAUCA^14KD_DAUCA^Q:321-569,H:54-136^67.47%ID^E:2e-28^RecName: Full=14 kDa proline-rich protein DC2.15
YL3_CGCTCATT-ATAGAGGC_comp101_seq3	redox2	34	MER_ERYCB^MER_ERYCB^Q:148-1101,H:4-327^54.77%ID^E:6e-114^RecName: Full=Methylecgonone reductase
AR1_GAGATTCC-CCTATCCT_comp1106_seq0	NA	34	CERK1_ARATH^CERK1_ARATH^Q:244-2034,H:22-617^59.74%ID^E:0^RecName: Full=Chitin elicitor receptor kinase 1
AR2_GAGATTCC-GGCTCTGA_comp12553_seq0	NA	34	LORF2_MOUSE^LORF2_MOUSE^Q:1716-3227,H:397-895^27.41%ID^E:2e-42^RecName: Full=LINE-1 retrotransposable element ORF2 protein
AR1_GAGATTCC-CCTATCCT_comp16225_seq0	NA	34	ACCH1_ARATH^ACCH1_ARATH^Q:118-960,H:12-288^50.18%ID^E:4e-73^RecName: Full=1-aminocyclopropane-1-carboxylate oxidase homolog 1

TableS2

AR1_GAGATTCC-CCTATCCT_comp30027_seq0	NA	34	.
AR2_GAGATTCC-GGCTCTGA_comp22761_seq0	NA	34	.
YL3_CGCTCATT-ATAGAGGC_comp5165_seq0	NA	35	Y5102_ARATH^Y5102_ARATH^Q:1752-3065,H:1-410^75.8%ID^E:0^RecName: Full=Serine/threonine-protein kinase At5g01020
OL1_CGCTCATT-CCTATCCT_comp374_seq0	GS1	35	MTDH_FRAAN^MTDH_FRAAN^Q:212-1258,H:10-356^57.02%ID^E:1e-115^RecName: Full=Probable mannitol dehydrogenase
AR2_GAGATTCC-GGCTCTGA_comp10111_seq1	NA	35	PYRD2_RAT^PYRD2_RAT^Q:122-1753,H:34-581^51.72%ID^E:0^RecName: Full=Pyridine nucleotide-disulfide oxidoreductase domain-containing protein 2
AR1_GAGATTCC-CCTATCCT_comp696_seq16	NA	35	BGH3B_BACO1^BGH3B_BACO1^Q:1249-2835,H:107-658^31.05%ID^E:4e-72^RecName: Full=Beta-glucosidase BoGH3B
YS2_GAGATTCC-ATAGAGGC_comp6183_seq2	NA	35	YIF1B_MOUSE^YIF1B_MOUSE^Q:488-1138,H:83-305^32.61%ID^E:5e-28^RecName: Full=Protein YIF1B
YL1_CGCTCATT-TATAGCCT_comp10608_seq0	NA	35	SFT2B_HUMAN^SFT2B_HUMAN^Q:233-616,H:27-158^39.39%ID^E:4e-23^RecName: Full=Vesicle transport protein SFT2B
AR1_GAGATTCC-CCTATCCT_comp977_seq0	NA	35	SF3BA_ARATH^SF3BA_ARATH^Q:186-446,H:1-87^94.25%ID^E:8e-51^RecName: Full=Uncharacterized protein At3g23325
AR1_GAGATTCC-CCTATCCT_comp6_seq2	NA	36	.
AR1_GAGATTCC-CCTATCCT_comp24292_seq0	CrWRKY1	36	WRK70_ARATH^WRK70_ARATH^Q:551-766,H:109-180^62.5%ID^E:2e-21^RecName: Full=Probable WRKY transcription factor 70
AR1_GAGATTCC-CCTATCCT_comp3465_seq0	NA	36	Y3272_ARATH^Y3272_ARATH^Q:414-1277,H:85-371^69.79%ID^E:4e-151^RecName: Full=Kelch repeat-containing protein At3g27220
AR1_GAGATTCC-CCTATCCT_comp572_seq0	NA	36	.
AR2_GAGATTCC-GGCTCTGA_comp1660_seq1	BIS2	37	BH093_ARATH^BH093_ARATH^Q:717-1313,H:146-351^66.02%ID^E:2e-66^RecName: Full=Transcription factor bHLH93
YS2_GAGATTCC-ATAGAGGC_comp11524_seq0	NA	37	FBK9_ARATH^FBK9_ARATH^Q:114-1199,H:15-383^41.98%ID^E:4e-74^RecName: Full=F-box/kelch-repeat protein At1g23390
OL3_CGCTCATT-GGCTCTGA_comp23128_seq0	NA	37	.
OL3_CGCTCATT-GGCTCTGA_comp28247_seq0	NA	37	.
OL3_CGCTCATT-GGCTCTGA_comp55923_seq0	NA	37	.
OL3_CGCTCATT-GGCTCTGA_comp21836_seq0	NA	37	.
AR1_GAGATTCC-CCTATCCT_comp8890_seq1	NA	38	AGL8_SOLTU^AGL8_SOLTU^Q:160-879,H:1-250^68.9%ID^E:2e-113^RecName: Full=Agamous-like MADS-box protein AGL8 homolog
AR1_GAGATTCC-CCTATCCT_comp12530_seq0	D4H	38	DV4H_CATRO^DV4H_CATRO^Q:56-1183,H:24-400^67.72%ID^E:0^RecName: Full=Deacetoxyvindoline 4-hydroxylase
AR1_GAGATTCC-CCTATCCT_comp13728_seq0	NA	38	.
AR2_GAGATTCC-GGCTCTGA_comp26468_seq0	NA	39	.
AR1_GAGATTCC-CCTATCCT_comp18788_seq0	MATE1	39	TT12_ARATH^TT12_ARATH^Q:204-1712,H:3-492^47.34%ID^E:5e-141^.^.

Table S3

Primers	Sequence (5' - 3')	Application
vm16OMTlike-for	CTGAGAAGATCTATGGATGTGCAAAGTGATGAGTTTAG	Heterologous expression of tabersonine 16-O-methyltransferase candidates in yeast
vm16OMTlike-rev	CTGAGAAGATCTTTAAGGATAAACCTCAATGACACTTCTAATG	
yYS1_comp1836_seq0-for	CTGAGAGGATCCATGGATCTTGAAGCTTCTGAGATTCGT	
yYS1_comp1836_seq0-rev	CTGAGAGGATCCAGGATATACCTCAATAGGACACCTGAAATCTA	
vm16OMTYFP-for	CTGAGAACTAGTATGGATGTGCAAAGTGATGAGTTTAG	Subcellular localization studies
vm16OMTYFP-rev	CTGAGAACTAGTAGGATAAACCTCAATGACACTTCTAATG	
qVmActin-for	GGAGCTGAGAGATTCCGTTG	qPCR analysis
qVmActin-rev	GAATTCCTGCAGCTTCCATC	
qVm16OMT-for - (YS2_comp61_seq0)	CAGTTATCTATGGACATGGGCATGA	
qVm16OMT-rev - (YS2_comp61_seq0)	TTAGAAATTGAATTAGTACATTGTCCCT	
qVmYL1_comp255_seq0-for	CTACTACACTTCCTCTTAAGAGACGT	
qVmYL1_comp255_seq0-rev	ACTTTGGGGCAAAATATATTCCACTAA	
qVmOL3_comp1418_seq0-for	TGTAGAACTTGCATTGGCAAATTTGAT	
qVmOL3_comp1418_seq0-rev	TCTGTTCTCGTTTGTTGTTCCACTAT	
qVmOL3_comp1489_seq0-for	GATATGACAGAGAAATTTGGGTTGACA	
qVmOL3_comp1489_seq0-rev	ACGCTTTCGCCTGGAATAATAAGTG	
qVmAR1_comp3108_seq0-for	CAGAACACAATATAGTTGCAGACGTT	
qVAR1_comp3108_seq0-rev	ACATAGGAAACACTCTAAAATCTGCAT	
qVmYS2_comp528_seq2-for	TTCCATTTTGATTGGAAACTTTATGGT	
qVmYS2_comp528_seq2-rev	AGGCATGTGCAAAATGTTGCAATCC	
